# Hospital Addiction Consultation Service and Opioid Use Disorder Treatment: The START Randomized Clinical Trial

**DOI:** 10.1001/jamainternmed.2024.8586

**Published:** 2025-04-07

**Authors:** Allison J. Ober, Cristina Murray-Krezan, Kimberly Page, Peter D. Friedmann, Jess Anderson, Karen Chan Osilla, Stephen Ryzewicz, Sergio Huerta, Mia W. Mazer, Randall A. Hoskinson, Richard Garvey, Alexandra Peltz, Katherine E. Watkins, Teryl Nuckols, Waguih William IsHak, Louis T. Mariano, Itai Danovitch

**Affiliations:** 1 RAND Corporation, Santa Monica, California; 2 University of Pittsburgh School of Medicine, Pittsburgh, Pennsylvania; 3 University of New Mexico Health Sciences Center, Albuquerque; 4 University of Massachusetts Chan Medical School–Baystate, Springfield; 5 Stanford School of Medicine, Palo Alto, California; 6 Cedars-Sinai Medical Center, Los Angeles, California; 7 RAND Corporation, Arlington, Virginia

## Abstract

**Importance:**

Medications for opioid use disorder (MOUD) are effective, but hospitalized people with opioid use disorder (OUD) seldom receive MOUD while in the hospital or link with treatment after.

**Objective:**

To test whether an addiction-focused consultation service, the Substance Use Treatment and Recovery Team (START), increases MOUD initiation during hospitalization and linkage to follow-up care after discharge.

**Design, Setting, and Participants:**

This 1:1 randomized clinical trial grouped participants into the START intervention or usual care. The study was conducted between November 2021 and September 2023 at 3 hospitals in the following cities: Los Angeles, California; Albuquerque, New Mexico; and Springfield, Massachusetts. The last follow-up was in December 2023. Eligible individuals were 18 years and older and met criteria for OUD.

**Intervention:**

START consists of an addiction medicine specialist and a care manager delivering a motivational and addiction-focused discharge planning intervention and follow-up calls.

**Main Outcomes and Measures:**

Primary outcomes were the proportions of patients (1) initiating MOUD (naltrexone, buprenorphine, or methadone) during hospitalization (per electronic medical record data) and (2) successfully linking to OUD treatment within 30 days after discharge (per patient self-report).

**Results:**

A total of 325 were consented and randomized to START (n = 164) or usual care (n = 161). Median (IQR) age was 41.0 (32.0-50.0) years. A total of 213 participants (65.5%) were male at birth, 28 (8.6%) were American Indian or Alaska Native, 21 (6.5%) were Black, 156 (48.0%) were Hispanic, and 125 (38.5%) were White. More than half, 175 (53.8%), were unhoused in the past year, and 163 (50.2%) were unemployed. START participants were more likely than usual care participants to initiate MOUD during hospitalization (94/164 [57.3%] vs 43/161 [26.7%], respectively; adjusted risk ratio [aRR], 2.10 [97.5% CI, 1.51-2.91]) and to link to OUD care after discharge (90/125 [72.0%] vs 50/104 [48.1%], respectively; aRR, 1.49 [97.5% CI, 1.15-1.93]).

**Conclusions and Relevance:**

By addressing gaps in inpatient care, the hospital-based addiction-focused consultation service presented in this randomized clinical trial improved receipt of evidence-based treatment for people with OUD in the hospital and linkage to treatment after discharge.

**Trial registration:**

ClinicalTrials.gov Identifier: NCT05086796

## Key Points

**Question:**

For hospitalized patients with opioid use disorders (OUDs), can an intervention by a hospital-based addiction consultation service called the Substance Use Treatment and Recovery Team (START) increase initiation of medication for OUD in the hospital and improve linkage to OUD-focused follow-up care compared with usual care?

**Findings:**

In this randomized clinical trial that included 325 adults, START participants were more likely than usual care participants to initiate medication for OUD during hospitalization (57.3% vs 26.7%, respectively) and to link to OUD care after discharge (72.0% vs 48.1%, respectively).

**Meaning:**

START, a hospital-based addiction consultation service, can improve receipt of evidence-based treatment for people with OUD, potentially reducing overdose and mortality.

## Teaser

This randomized clinical trial compares usual care to an addiction-focused consultation service in hospitalized patients with opioid use disorder.

## Introduction

The US opioid epidemic continues to be an urgent public health crisis, with 81 083 opioid overdose deaths in 2023.^1^ Despite the availability of effective medication for opioid use disorder (MOUD), few hospitalized individuals initiate treatment^2^ or link to postdischarge services.^3,4,5,6^

Hospitalization represents a pivotal opportunity to engage patients with opioid use disorder (OUD) in evidence-based treatment. Hospital admissions for OUD have risen dramatically, from 301 707 in 2002 to an estimated 941 700 in 2018, with a corresponding surge in associated costs.^7^ These hospitalizations, whether primarily related to opioid use or other medical conditions, create a unique window for intervention, as hospitals possess the essential resources: physicians authorized to prescribe MOUD and support staff who can facilitate postdischarge care coordination. This opportunity is particularly important for patients who may face barriers to accessing MOUD in the health care system and other community access points due to stigma, unstable housing, costs, logistical issues, lack of knowledge among practitioners, racial and ethnic discrimination, and other social determinants of health. Hospitalization provides a structured, supportive environment where these barriers can be temporarily overcome, allowing for direct engagement with specialized addiction care, treatment initiation, and connection to ongoing support services.

Hospital-based addiction consultation services (ACS) demonstrate substantial potential to enhance the implementation of MOUD during acute hospitalization and for facilitating postdischarge treatment engagement.^8,9,10^ A range of ACS care models have been described, with varied roles comprising an interprofessional team capable of delivering evidenced-based interventions and supportive services.^11^ Among the ACS models described in the literature, the most common roles are that of a physician to medically manage substance use disorders and a care manager (CM) to deliver brief interventions and coordinate linkage to aftercare.^9,11^ Although several observational studies suggest potential impact of ACS on improving hospital-based treatment for people with substance use disorders^9^ and a recent study demonstrated that an ACS can improve postdischarge MOUD initiation and retention,^12^ no parallel-assignment randomized clinical trials have compared ACS effectiveness to usual care. Further, ACS research has not tested effects of an ACS with specified intervention components.

This article presents findings from a multisite randomized clinical trial conducted at 3 geographically distinct US hospitals, testing an inpatient ACS model called the Substance Use Treatment and Recovery Team (START). The START ACS model, composed of an addiction medicine specialist (AMS) and CM team and drawing from the Collaborative Care Model,^13,14^ Motivational Interviewing,^15^ Project Reengineered Discharge,^16,17^ and American Society of Addiction Medicine standards of care for managing OUD,^18^ delivers motivational interviewing and enhanced discharge planning, followed by 1 month of follow-up calls. This study tested the hypothesis that the START would improve in-hospital MOUD initiation and postdischarge care linkage compared to usual care.

## Methods

### Study Design and Setting

The START study was a 3-site pragmatic randomized clinical trial testing the effects of START vs usual care on MOUD initiation and postdischarge linkage. The trial was conducted at Cedars-Sinai Medical Center in Los Angeles, California; the University of New Mexico Hospital in Albuquerque, New Mexico; and Baystate Medical Center in Springfield, Massachusetts. Recruitment for the study began in November 2021 and ended in September 2023; the last follow-up interview was conducted in December 2023. The Cedars-Sinai Medical Center Institutional Review Board reviewed all study procedures under a single review model. All patients provided written informed consent. Full study details can be found in the trial protocol and statistical analysis plan (Supplement 1). We report this study in accordance with the Consolidated Standards of Reporting Trials (CONSORT) reporting guideline.

### Study Population

Eligibility criteria included the following: (1) current inpatient at 1 of the 3 hospitals; (2) 18 years or older; (3) probable OUD diagnosis, defined by scores of greater than 3 on the opioid section of the World Health Organization Alcohol, Smoking, and Substance Involvement Screening Test (ASSIST)^19^; (4) English or Spanish as a primary language; (5) life expectancy of greater than 6 months (ie, not in hospice); and (6) able to provide informed consent. Participants already receiving MOUD during their current hospitalization were not eligible. Approved study staff prescreened patients for potential enrollment through a daily electronic medical record report based on an algorithm of risk factors for OUD and diagnosis codes. On consent from the requesting medical team (required at 2 of the 3 hospitals for participation in a research study), study staff conducted eligibility screening. The screening was conducted by research staff in person or remotely using an approved and secure web-based data capture system (REDCap [Vanderbilt University])^20^ housed at the study statistics and data coordinating center at the University of New Mexico. The eligibility screener assessed presence of moderate to severe opioid use using ASSIST (indicated by a score of 4 or higher), as well as moderate to severe use of alcohol (11 or higher) and other substances (4 or higher), along with age, sex (assigned at birth), gender identity, Hispanic/Latino ethnicity, housing status, and self-reported prior diagnosis of bipolar disorder or schizophrenia.

### Baseline Assessment

After obtaining written consent to participate and enrolling participants, study staff conducted an in-person or virtual 30-minute to 40-minute baseline interview. This interview assessed employment status, criminal justice involvement, income in the prior year, marital status, education, past 30-day substance use,^21^ severity of substance use using the 7-item Patient-Reported Outcomes Measurement Information System (PROMIS) substance use severity measure,^22^ prior use of MOUD and treatment for OUD, overdose history, pain intensity using 1 question from the Pain, Enjoyment of Life and General Activity (PEG) scale,^23^ a locally developed question about pain duration, and current mental health symptoms, using the Patient Health Questionnaire–9 (PHQ-9)^24,25^ to assess depression and the Generalized Anxiety Disorder Assessment–7 (GAD-7)^26,27,28^ to assess anxiety. Patients received a $50 incentive for completing the baseline assessment.

### Randomization and Enrollment

Following the baseline interview, patients were randomized 1:1 to receive START or usual care using block randomization with block sizes of 2, 4, and 8 (all programmed into REDCap), stratified by site and prior MOUD exposure (any vs none). The allocation plan was programmed by coauthor C.M. in R software, version 4.0 (R Project for Statistical Computing), using the package Blockrand. There was no masking to the treatment group. All patients randomized were considered enrolled in the study.

### Follow-Up Assessment

Follow-up interviews were conducted by telephone by the RAND Survey Research Group 1 month after the patient was discharged from the hospital, with up to a 60-day follow-up window. Follow-up interviews assessed utilization of services related to OUD. Interviewers were not masked to study intervention.

### Descriptions of the START Intervention and Usual Care

START is an interprofessional ACS composed of an AMS and CM team that delivers a tailored intervention based on motivational interviewing^29^ and addiction-focused discharge planning.^16,17^ In this study, AMSs were board-certified physicians knowledgeable about the American Society for Addiction Medicine's standards of care for managing OUD and capable of initiating and managing MOUD. AMSs were already present at each hospital. CMs were social workers or case managers with knowledge about and experience with addiction treatment. The START provides diagnostic assessments, makes clinical recommendations, assists with the implementation of treatment plans, establishes OUD-focused discharge plans, facilitates linkage to treatment after discharge, and provides follow-up telephone calls for 1 month (Table 1). Of note, our study included a preimplementation organizational context assessment that helped us refine our intervention and its implementation to support the needs of each hospital.^30^

**Table 1.** Substance Use Treatment and Recovery Team (START) Components

Component	START addiction medicine specialist	START care manager
(1) Triage		
Address acute biomedical needs (eg, facilitate withdrawal management)	NA	Yes
Assess the patient for acute psychosocial needs related to the OUD	Yes	NA
(2) Engage, assess, plan		
Engage		
Engage with the patient and family	Yes	Yes
Assess		
Conduct diagnostic and psychosocial assessment	NA	Yes
Conduct biomedical assessment and address comorbidities	Yes	NA
Plan		
Assess and increase readiness using the Brief Negotiated Interview	NA	Yes
Develop plan for initiating evidence-based treatment/MOUD during and after the hospital stay (Brief Negotiated Interview plus Project Reengineered Discharge)	NA	Yes
(3) Treat		
Facilitate management of intoxication, withdrawal symptoms, comorbidities, and MOUD initiation	Yes	NA
Facilitate psychosocial treatment for OUD, if indicated and available in the hospital	NA	Yes
(4) Communicate and coordinate		
Team communication to continue care through patient stay and ≥1 mo after the patient is discharged	Yes	Yes
Communicate with patient and medical team, and, when appropriate, patient’s family and outpatient clinicians	Yes	Yes
(5) Follow-up		
Call patient once a week for 1 mo after patient is discharged from the hospital to assess whether patient is following through with discharge plan	NA	Yes
Call outpatient clinicians to determine if the patient is linked to care and has encountered barriers	Yes	Yes

Abbreviations: MOUD, medication for opioid use disorder; NA, not applicable; OUD, opioid use disorder.

Usual care consisted of each hospital’s respective practices for managing patients identified with OUD (Supplement 1). Cedars-Sinai Medical Center had an existing consultation liaison service with psychiatrists and social workers who could discuss opioid use with the patient and help the patient initiate MOUD if indicated. At Baystate Medical Center and the University of New Mexico, patients randomized to the usual care study condition could be treated directly with MOUD and provided discharge planning by the medical team. Although the START AMS at each hospital had previously served as each hospital’s AMS, they did not see patients randomized to the usual care group during the study.

### Training and Fidelity

For the START intervention, the AMS and CM from each site participated in an initial 4-hour training and monthly follow-up calls conducted by a licensed psychologist and experienced motivational interviewing trainer. Competency in using motivational interviewing was measured using the Motivational Interviewing Treatment Integrity Coding Manual 4.2.1,^31^ which has metrics for scoring fair and good proficiency in using motivational interviewing. To assess fidelity to the START intervention, we assessed completion of 4 intervention components: (1) entry into the patient registry; (2) team-based care; (3) evidence-based resources provided; (4) follow-up calls.

### Primary Outcomes

Primary outcome 1 was the proportion of patients in each group who initiated MOUD prior to discharge, defined as use of any US Food and Drug Administration–approved pharmacotherapy for OUD, including buprenorphine, naltrexone, and methadone. Primary outcome 2 was the proportion of patients in each group who attended at least 1 OUD-related care visit within 30 days of hospital discharge, including a visit to any prescriber for MOUD or a psychosocial treatment visit.

### Secondary Outcomes

Secondary outcomes 1 to 3 were the proportion of patients in each group who (1) had an addiction focused discharge plan in the electronic medical record, defined as having an appointment made at or referral to a treatment program or clinician; (2) initiated or continued MOUD after discharge; and (3) had a visit to a clinician for their OUD. Secondary outcome 4 was the number of opioid use days in the 30 days after discharge, with use days defined as the total number of days (0-30) that the patient used any opioids, which were asked about in the following 4 categories: pain medications excluding fentanyl, fentanyl, heroin/opium alone, and heroin/opium mixed with another drug. If a patient used opioids in 2 or more categories on the same day, each would count as a use day. Possible use days ranged from 0 to 120 days.

### Statistical Analysis

Analyses were performed in the intention-to-treat population, which consisted of all eligible and consented participants randomized to either START or usual care. We summarized baseline characteristics with descriptive statistics, including means and SDs or medians and IQRs for continuous variables and frequencies and percentages for categorical variables. Continuous baseline demographics and characteristics of START vs usual care participants were assessed for homogenous distributions with Kolmogorov-Smirnov tests. Categorical variables were compared with χ^2^ or Fisher exact tests. All analyses were performed in SAS statistical software, version 9.4 (SAS Institute).

We performed our analyses on the intention-to-treat population, which consisted of all randomized participants. Our original proposed analysis was to fit multivariable logistic regression models to the primary outcomes and report odds ratios for effects (Supplement 1); however, we found that the MOUD initiation rates and linkage-to-care rates were higher in both groups than originally hypothesized. Odds ratios are frequently used to report effect sizes for dichotomous outcomes, but when outcome rates are not relatively rare, odds ratios may be artificially inflated, leading to overinterpretation of results.^32,33^ Because our outcome rates were not relatively rare, we performed multivariable Poisson regression to estimate a more accurate measure, the risk ratio, to better represent the outcome rates and prevent misinterpretation of results.^32,33^ See the statistical analysis plan in Supplement 1 for additional details.

Primary outcomes were compared between groups by fitting a multivariable Poisson regression model with robust standard errors to each end point that included the following independent variables: intervention group, prior MOUD exposure and site, age, insurance status (as a marker for income), race, ethnicity, unstable housing vs stable, and length of hospital stay. Four participants were still hospitalized at the end of the study period, and their discharge dates were censored. Adjusted risk ratios (aRRs) and Bonferroni-adjusted 97.5% Wald CIs are reported for the 2 primary end points. Secondary outcomes 1 to 3 also were compared between groups by a multivariable Poisson regression model for each end point. To evaluate secondary outcome 4, we fit a negative binomial regression model with a logarithmic link function to opioid use days. We report aRRs (secondary outcomes 1-3) or adjusted incident rate ratio (secondary outcome 4) and their 95% CIs. All models included the independent variables described for the primary outcomes with the addition of baseline opioid use days for secondary outcome 4. Baseline covariates used in the models had no missing data except for insurance status (5 missing values), which was not associated with outcomes and so was excluded from final models, and baseline opioid use days (3 missing values). No methods for missing data were implemented (Supplement 1). We report 97.5% CIs for the primary outcomes and 95% CIs for all other outcomes.

## Results

### Enrollment and Follow-Up

Of 442 patients screened, 345 were eligible, and 325 were randomized: 164 to START and 161 to usual care (Figure). Of 325 randomized patients, 229 (70.5%) had a 30-day postdischarge follow-up interview. Participants in the START group were more likely to participate in a follow-up interview compared to patients receiving usual care (125/164 [76.2%] vs 104/161 [64.6%], respectively).

**Figure.**
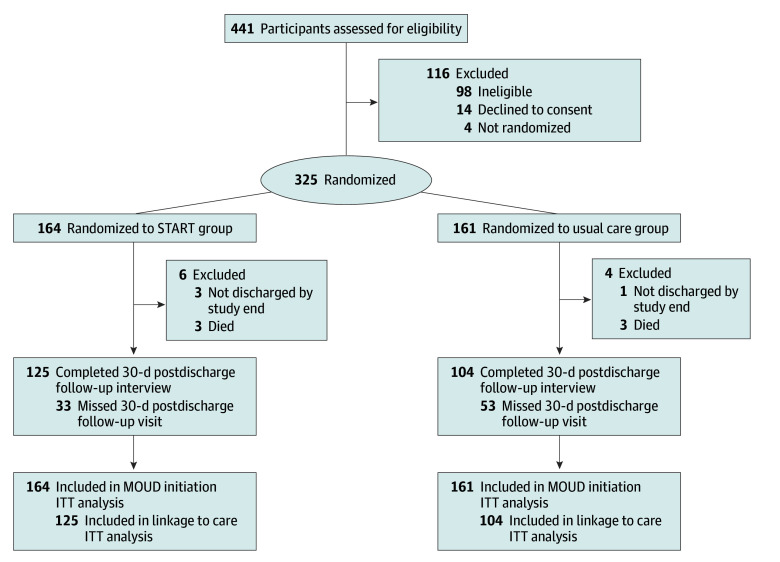
CONSORT Diagram ITT indicates intention-to-treat; MOUD, medication for opioid use disorder; START, Substance Use Treatment and Recovery Team.

### Baseline Characteristics

Median (IQR) age was 41.0 (32.0-50.0) years. A total of 213 participants (65.5%) were male at birth, 28 (8.6%) were American Indian or Alaska Native, 21 (6.5%) were Black, 156 (48.0%) were Hispanic, and 125 (38.5%) were White. More than half, 175 (53.8%), were unhoused in the past year, and 163 (50.2%) were unemployed. A total of 229 participants (70.5%) completed the follow-up interview. Most participants in both the START and usual care groups had previous exposure to MOUD (131 [79.9%] and 127 [78.9%], respectively; Table 2).

**Table 2.** Characteristics of Study Participants at Baseline

Characteristic	No. (%)	
	START (n = 164)	Usual care (n = 161)
Age, median (IQR), y	41.5 (34.0-50.0)	40.0 (31.0-51.0)
Sex		
Female	64 (39.0)	48 (29.8)
Male	100 (61.0)	113 (70.2)
Hispanic/Latino ethnicity	81 (49.4)	75 (46.6)
Race		
American Indian/Alaska Native	12 (7.3)	16 (9.9)
Asian/Pacific Islander	2 (1.2)	1 (0.6)
Black	9 (5.5)	12 (7.5)
White	66 (40.2)	59 (36.6)
Multiracial	10 (6.1)	13 (8.1)
Other^a^	65 (39.6)	60 (37.3)
Housing status		
Unhoused in past year	87 (53.0)	88 (54.7)
Employment status		
Working full-time or part-time	36 (22.0)	34 (21.1)
Unemployed, looking for work	22 (13.4)	15 (9.3)
Unemployed, not looking for work	56 (34.1)	70 (43.5)
Retired/disabled	50 (30.5)	42 (26.1)
Marital status		
Single (never married, not in a couple)	87 (53.0)	91 (56.5)
Widowed, divorced, or separated (not in a couple)	40 (24.4)	28 (17.4)
Married	27 (16.5)	29 (18.0)
Unmarried couple	10 (6.1)	11 (6.8)
Prefer not to answer	0 (0.0)	2 (1.2)
Highest level of education		
Less than high school	39 (23.8)	48 (29.8)
High school diploma or GED credential	59 (36.0)	57 (35.4)
Some college or more	66 (40.2)	55 (34.2)
Prefer not to answer	0 (0.0)	1 (0.6)
Public health insurance	141 (86.0)	134 (83.2)
Moderate to severe SUD (per ASSIST score)^b^		
Alcohol	48 (29.3)	45 (28.0)
Heroin or opium	118 (72.0)	104 (64.6)
Prescription opioids	121 (73.8)	126 (78.3)
Cannabis	96 (58.5)	95 (59.0)
Methamphetamine	90 (54.9)	91 (56.5)
Cocaine	56 (34.1)	52 (32.3)
Severity of OUD (per PROMIS), median (IQR)	59.5 (54.2-64.8)	60.3 (53.3-65.7)
Medication for OUD		
Ever	131 (79.9)	127 (78.9)
No. of times ever started, median (IQR)	3 (2-10)	3 (2-24)
OUD treatment other than medication		
Ever	90 (54.9)	88 (54.7)
No. of times ever started, median (IQR)	3 (1-5)	3 (2-6)
SUD treatment utilization		
Any SUD treatment in 90 d prior to hospitalization	78 (47.6)	84 (52.2)
Overdose history		
No. of times in life ever overdosed, median (IQR)	1 (0-3)	1 (0-4)
Pain (per PEG)		
Intensity (range, 0-10), median (IQR)	8 (7-10)	9 (7-10)
Duration (range, 1-4),^c^ median (IQR)	1 (1-3)	1 (1-3)
Depression (per PHQ-9 sum; range, 0-27)^d^		
Total score, median (IQR)	17 (10-24)	18 (10-23)
Anxiety (per GAD-7 sum; range, 0-21)		
Total score, median (IQR)	15 (7-20)	15 (9-20)
Mental illness history		
Ever diagnosed with bipolar disorder	62 (37.8)	65 (40.4)
Ever diagnosed with schizophrenia or schizoaffective disorder	25 (15.2)	33 (20.5)
Ever hospitalized for a psychiatric disorder	45 (27.4)	58 (36.0)
Elixhauser Comorbidity Index, median (IQR)	1.0 (0-1.5)	0 (0-1.0)

Abbreviations: ASSIST, Alcohol, Smoking, and Substance Involvement Screening Test; GAD-7, Generalized Anxiety Disorder Assessment–7; GED, General Educational Development; PROMIS, Patient-Reported Outcomes Measurement Information System; OUD, opioid use disorder; PEG, Pain, Enjoyment of Life and General Activity; PHQ-9, Patient Health Questionnaire–9; START, Substance Use Treatment and Recovery Team; SUD, substance use disorder.

^a^ Other indicates individuals who self-reported that they were a different race or ethnicity than the options presented (Alaska Native, American Indian, Asian, Black, Native Hawaiian, other Pacific Islander, or White).

^b^ Categories are not mutually exclusive.

^c^ Score of 1 indicates approximately 1 week; 4 indicates more than 6 months.

^d^ Missing responses: 5 for insurance; 2 for marital status; 1 for education, 1 for severity of OUD (PROMIS), 1 for PHQ-9, 1 for GAD-7.

### Randomized Clinical Trial Outcomes

START participants were more likely than usual care participants to initiate MOUD during hospitalization (94/164 [57.3%] vs 43/161 [26.7%], respectively; aRR, 2.10 [97.5% CI, 1.51-2.91]) and to link to OUD care after discharge (90/125 [72.0%] vs 50/104 [48.1%], respectively; aRR, 1.49 [97.5% CI, 1.15-1.93]) (Table 3). In both groups, 90 patients who received MOUD (66%) received methadone while in the hospital. A total of 36 START (38.3%) and 17 usual care (39.5%) patients, respectively, who received MOUD received buprenorphine, while 5 (5.3%) and 1 (2.3%) START and usual care patients, respectively, received both methadone and buprenorphine. Two START (1.0%) and no usual care patients received both methadone and oral naltrexone. Of additional independent variables tested, only length of stay was associated with MOUD initiation; however, there was no interaction between length of stay and treatment group meaning that the effects were the same in both groups (every 10 days of stay increased the likelihood of MOUD initiation by 3.8% (aRR, 1.04 [95% CI, 1.01-1.07]). The 2 groups were comparable with respect to demographics and characteristics at baseline.

**Table 3.** Primary Outcomes: Substance Use Treatment and Recovery Team (START) vs Usual Care

Primary outcome	No./total No. (%)		aRR (97.5% CI)
	START	Usual care	
Initiated MOUD treatment in hospital^a^	94/164 (57.3)	43/161 (26.7)	2.10 (1.51-2.91)^b^
Linked to care following discharge^c^	90/125 (72.0)	50/104 (48.1)	1.49 (1.15-1.93)^d^

Abbreviations: aRR, adjusted risk ratio; MOUD, medication for opioid use disorder.

^a^ The denominators were the total number of patients randomized.

^b^ aRR for stratification variables: site and prior MOUD exposure and length of hospital stay.

^c^ The denominators were the total number of patients who had a 1-month follow-up visit.

^d^ aRR for stratification variables: site and prior MOUD exposure (neither significant).

For the secondary outcomes, START patients were significantly more likely than usual care patients to (1) have an OUD-focused discharge plan in the medical record (81/164 [49.4%] vs 44/161 [27.3%], respectively; aRR, 1.8 [95% CI, 1.36-2.41]); (2) initiate or continue MOUD after hospital discharge (65/124 [52.4%] vs 32/104 [30.8%], respectively; aRR, 1.71 [95% CI, 1.23-2.39]); and (3) see a clinician for their OUD after discharge (42/122 [34.4%] vs 19/104 [18.3%], respectively; aRR, 1.89 [95% CI, 1.18-3.03]). The median (IQR) opioid use days at follow-up was 0 (0-10) for START patients and 0 (0-14) for usual care patients. There was no difference in opioid use days between the 2 groups following the intervention (adjusted incident rate ratio, 1.25 [95% CI, 0.64-2.43]; Table 4).

**Table 4.** Secondary Outcomes: Substance Use Treatment and Recovery Team (START) vs Usual Care

Secondary outcome^a^	START	Usual care	aRR (95% CI)^b^^,^^c^
(1) OUD-specific discharge plan in medical record			
No./total No. of participants (%)	81/164 (49.4)	44/161 (27.3)	1.80 (1.36 to 2.41)
(2) Initiated or continued MOUD after discharge			
No./total No. of participants (%)	65/124 (52.4)	32/104 (30.8)	1.71 (1.23 to 2.39)
(3) Obtained postdischarge outpatient medical care related to OUD			
No./total No. of participants (%)	42/122 (34.4)	19/104 (18.3)	1.89 (1.18 to 3.03)
(4A) Opioid use days in past 30 d at follow-up^d^			
Total No. of participants	122	103	1.25 (0.64 to 2.43)^b^^,^^e^
Median (IQR)	0 (0 to 10.0)	0 (0 to 14.0)	
(4B) Change in opioid use days in past 30 d (follow-up − baseline)^f^			
Total No. of participants	120	102	NA
Median (IQR)	−13.0 (−30.0 to 0)	−4.5 (−30.0 to 0)	

Abbreviations: aIRR, adjusted incident rate ratio; aRR, adjusted risk ratio; MOUD, medication for opioid use disorder; NA, not applicable; OUD, opioid use disorder.

^a^ Denominators for secondary outcome 1 are all randomized participants; for secondary outcomes 2 to 4 are those who had a 1-month follow-up visit and answered the question.

^b^ aRR or aIRR for stratification variables: site and prior MOUD exposure; secondary outcome 4 also includes baseline opioid use days.

^c^ Risk ratios calculated from Poisson regression models for secondary outcomes 1 to 3.

^d^ Opioid use days range from 0 to 120 days across 4 types of opioids.

^e^ This value represents the adjusted incident rate ratio calculated from negative binomial regression model for secondary outcome 4.

^f^ Change in opioid use days ranges from −120 to 120 days across 4 types of opioids.

### START Fidelity Assessment

Fidelity to the START intervention was high. The majority, 149 (91%), received all 4 components: (1) patient registry: 162 START patients (99%) had an entry in the patient registry; (2) team-based care: 156 patients (95%) were discussed between the CM and AMS, with 153 (93%) seen by the CM at least once, and 153 (93%) seen by the AMS at least once; (3) evidence-based resources: 144 patients (88%) received these (pros and cons worksheet, readiness rulers, and discharge plan review); and (4) follow-up: CMs attempted follow-up calls with 161 patients (98%); 100 patients (61%) received at least 1 follow-up call.

## Discussion

This multisite, parallel assignment randomized clinical trial demonstrated that the START ACS was superior to usual care. Patients who engaged with the START were more likely to begin MOUD while in the hospital and successfully transition to community-based treatment after discharge. The intervention’s benefits extended beyond just MOUD; START patients also received more comprehensive discharge planning focused on their OUD and were more likely to receive MOUD and to engage in medical care for their OUD after leaving the hospital.

Our study contributes to a growing body of literature suggesting that an inpatient ACS can have a positive effect on treatment initiation and linkage to postdischarge care.^12,34,35^ A recent pragmatic stepped-wedge study conducted by McNeely et al^12^ demonstrated that an ACS improves postdischarge MOUD initiation and retention. Of note, a high proportion of patients in both groups of our study (72.0% in START and 48.1% in usual care) linked to treatment for OUD after the hospital, findings that exceeded linkage outcomes in the study by McNeely et al.^12^ This may reflect our broader definition of postdischarge treatment, which included any type of treatment for OUD, not just MOUD. High linkage rates in START and usual care groups could also explain why we found no significant difference in opioid use between START and usual care patients in the 30 days following discharge.

Hospital admissions offer a crucial opportunity to engage patients in evidence-based treatments for OUD. Patients may be more receptive to care during hospitalization, particularly when their admission relates to opioid use complications. By offering motivational support and connections to community care, hospitals can help reduce stigma and create more accessible pathways to recovery. This patient-centered approach has the potential to reduce the consequences of opioid use, including overdose and mortality, as well as mitigate the numerous other adverse effects associated with ongoing opioid use.

The START ACS model addresses key barriers to treating substance use disorders in hospital settings, including insufficient expertise within inpatient teams, low patient readiness, and the need for transitional care and outreach. By tackling these challenges, the START ACS model bridges critical gaps and enhances the likelihood of successful treatment for individuals with OUD. With appropriate adaptations to various contexts and populations and supported by additional evidence, this approach is applicable to other substance use disorders. Furthermore, it can address the current shortage of hospital-based linkage strategies for people with substance use disorders, ultimately improving continuity of care and long-term outcomes.^6^

### Strengths and Limitations

Our study has several strengths. START was implemented in 3 diverse hospital settings, with no differences in findings among sites, suggesting the broad generalizability of the intervention to diverse patients receiving services in large, urban medical settings. The hospitals, which serve low-income individuals from a variety of racial and ethnic backgrounds, included a diverse sample of people with OUD, including a large proportion of American Indian and Hispanic individuals as well as a large proportion of unhoused people. Treatment outcomes (MOUD initiation or linkage) did not differ based on race, ethnicity, income or housing status, or other demographic covariates, suggesting that the START model can provide equitable treatment for underserved individuals with OUD. This finding is critical given the rapid acceleration of overdose mortality among people who are American Indian or Alaska Native, Black, or Hispanic,^36^ and because racial and ethnic minority individuals typically are less likely to receive MOUD (buprenorphine in particular) than those who are White and those living in high-income areas.^37,38,39^ Further, the START leveraged existing AMSs and care managers, which supports the feasibility of future implementation, dissemination, and sustainability.

We note a few limitations to our study. We assessed linkage outcomes by self-report, which could be influenced by social desirability bias; however, this would have had the same effect in each study condition. Additionally, several factors may limit the generalizability of study findings to other hospitals, including having large enough populations to warrant a dedicated addiction-focused consultation service, resources to implement an ACS, and sufficient community-based resources to link patients to care after discharge.

## Conclusions

The undertreatment of OUD continues to present a critical public health challenge. The START ACS model presented in this randomized clinical trial provides a promising solution by addressing key roadblocks that contribute to the undertreatment of OUD in hospital settings and poor linkage to postdischarge care and offers a practical approach to hospital-based care that can significantly improve treatment uptake for individuals with OUD. Moreover, the ACS is a translational model in that it has the potential to be adapted and applied to enhance treatment uptake for people with other substance use disorders and behavioral health problems, offering a comprehensive approach to addressing widespread health care gaps.

## Notes

Supplement 1. Trial Protocol and Statistical Analysis Plan

Supplement 2. Data Sharing Statement
