# Supplementary material for: Hospital Addiction Consultation Service and Opioid Use Disorder Treatment: The START Randomized Clinical Trial
Source: JAMA Intern Med. 2025 Apr 7;185(6):624–33. doi: 10.1001/jamainternmed.2024.8586 (PMC11976642; doi:10.1001/jamainternmed.2024.8586)
Supplement: Supplement 1. — Trial Protocol and Statistical Analysis Plan [file jamainternmed-e248586-s001.pdf]

# Official Grant Title: Collaborative Care Teams for Hospitalized Patients with Opioid Use Disorders

## Study/Intervention Title: Substance Use Treatment and Recovery Team (START)

**National Clinical Trial (NCT) Identified Number:**

**NCT05086796**

**Principal Investigators:**
Itai Danovitch, MD, Cedars-Sinai Medical Center (MPI)
Allison J. Ober, PhD, RAND (MPI)

Peter D. Friedmann, MD, MPH, Baystate Health (Site PI)
Kimberly Page, PhD, MPH, University of New Mexico (Site PI)

**Sponsor: Cedars-Sinai Medical Center**

**Grant Number: 1U01TR002756-01A1**

**Funded by: National Center for Advancing Translational Sciences, National Institute on Drug Abuse**

**Version Number: 1.7**

**08 AUGUST 2023**

**CONFIDENTIALITY STATEMENT**

This document is confidential communication. Acceptance of this document constitutes agreement by the recipient that no unpublished information contained herein will be published or disclosed without prior approval of the Principal Investigators or other participating study leadership and as consistent with the NIH terms of award.

# Table of Contents

STATEMENT OF COMPLIANCE ..... 6
INVESTIGATORS' SIGNATURES ..... 7
1 PROTOCOL SUMMARY ..... 9
1.1 Synopsis ..... 9
1.2 Schema ..... 12
1.3 Schedule of Activities ..... 13
2 INTRODUCTION ..... 14
2.1 Study Rationale ..... 14
2.2 Background ..... 14
2.3 Risk/Benefit Assessment ..... 18
2.3.1 Known Potential Risks ..... 18
2.3.2 Known Potential Benefits ..... 18
2.3.3 Assessment of Potential Risks and Benefits ..... 19
3 OBJECTIVES AND ENDPOINTS ..... 21
4 STUDY DESIGN ..... 22
4.1 Overall Design ..... 22
4.2 Scientific Rationale for Study Design ..... 24
4.3 Justification for Intervention ..... 25
4.4 End-of-Study Definition ..... 25
5 STUDY POPULATION ..... 25
5.1 Inclusion Criteria ..... 25
5.2 Exclusion Criteria ..... 26
5.3 Lifestyle Considerations ..... 26
5.4 Screen Failures ..... 26
5.5 Strategies for Recruitment and Retention ..... 26
5.5.1 Patient Identification and Recruitment ..... 26
5.5.2 Consent AND Enrollment ..... 27
5.5.3 Retention ..... 27
5.5.4 Incentives ..... 27
5.5.5 anticipated enrollment ..... 28
6 STUDY INTERVENTIONS ..... 30
6.1 Study Intervention Administration ..... 30
6.1.1 Study Intervention Description ..... 30
6.1.2 Usual Care Description ..... 31
6.1.3 Administration and/or Dosing ..... 31
6.2 Fidelity ..... 32
6.2.1 Interventionist Training ..... 32
6.2.2 Fidelity measures ..... 32
6.3 Measures to Minimize Bias: Randomization and Blinding ..... 33

6.4 Study Intervention/Experimental Manipulation Adherence ..... 34
6.5 Concomitant Therapy ..... 34
6.5.1 Rescue Therapy ..... 34
7 STUDY INTERVENTION/EXPERIMENTAL MANIPULATION DISCONTINUATION AND PARTICIPANT DISCONTINUATION/WITHDRAWAL ..... 34
7.1 Discontinuation of Study Intervention/Experimental Manipulation ..... 34
7.2 Participant Discontinuation/Withdrawal from the Study ..... 35
7.3 Lost to Follow-Up ..... 35
8 STUDY ASSESSMENTS AND PROCEDURES ..... 35
8.1 Endpoint and Other Non-Safety Assessments ..... 35
8.1.1 Eligibility Screening ..... 36
8.1.2 Baseline Interview ..... 36
8.1.3 1-Month Follow-Up Interview ..... 36
8.1.4 Procedural Flow ..... 36
8.1.5 Variables Collected ..... 37
8.2 Safety Assessments ..... 41
8.3 Adverse Events and Serious Adverse Events ..... 41
8.3.1 Definition of Adverse Events ..... 41
8.3.2 Definition of Serious Adverse Events ..... 41
8.3.3 Classification of an Adverse Event ..... 42
8.3.4 Time Period and Frequency for Event Assessment and Follow-Up ..... 43
8.3.5 Adverse Event Reporting ..... 44
8.3.6 Serious Adverse Event Reporting ..... 45
8.3.7 Reporting Events to Participants ..... 46
8.3.8 Events of Special Interest ..... 46
8.3.9 Reporting of Pregnancy ..... 46
8.4 Unanticipated Problems ..... 51
8.4.1 Definition of Unanticipated Problems ..... 51
8.4.2 Unanticipated Problems Reporting ..... 51
8.4.3 Reporting Unanticipated Problems to Participants ..... 52
9 STATISTICAL CONSIDERATIONS ..... 52
9.1 Statistical Hypotheses ..... 52
9.2 Sample Size Determination ..... 53
9.3 Populations for Analyses ..... 54
9.4 Statistical Analyses ..... 54
9.4.1 General Approach ..... 54
9.4.2 Analysis of Primary and Secondary Endpoints ..... 54
9.4.3 Safety Analyses ..... 55
9.4.4 Baseline Descriptive Statistics ..... 55
9.4.5 Planned Interim Analyses ..... 56

9.4.6 Sub-Group Analyses ..... 56
9.4.7 Tabulation of Individual Participant Data ..... 56
9.4.8 Exploratory Analyses ..... 56
10 SUPPORTING DOCUMENTATION AND OPERATIONAL CONSIDERATIONS ..... 57
10.1 Regulatory, Ethical, and Study Oversight Considerations ..... 57
10.1.1 Informed Consent Process ..... 57
10.1.2 Study Discontinuation and Closure ..... 57
10.1.3 Confidentiality and Privacy ..... 58
10.1.4 Future Use of Stored Specimens and Data ..... 59
10.1.5 Key Roles and Study Governance ..... 59
10.1.6 Safety Oversight ..... 60
10.1.7 Clinical Monitoring ..... 61
10.1.8 Quality Assurance and Quality Control ..... 61
10.1.9 Data Handling and Record Keeping ..... 61
10.1.10 Protocol Deviations ..... 64
10.1.11 Publication and Data Sharing Policy ..... 65
10.1.12 Conflict of Interest Policy ..... 65
10.2 Additional Considerations ..... 66
10.3 Abbreviations and Special Terms ..... 66
10.4 Protocol Amendment History ..... 67
11 APPENDICES ..... 67
11.1 Recruitment and Enrollment ..... 67
11.2 Evaluation ..... 68
11.3 Data Safety And Monitoring ..... 68
12 REFERENCES ..... 69

## STATEMENT OF COMPLIANCE

The trial will be conducted in accordance with International Council on Harmonisation Good Clinical Practice (ICH GCP), applicable United States (US) Code of Federal Regulations (CFR), and the National Center for Advancing Translational Sciences Terms and Conditions of Award. The Principal Investigators will assure that no deviation from, or changes to the protocol will take place without prior agreement from the funding agency and documented approval from the Institutional Review Board (IRB), except where necessary to eliminate an immediate hazard(s) to the trial participants. All personnel involved in the conduct of this study have completed Human Subjects Protection and ICH GCP Training.

The protocol, informed consent form(s), recruitment materials, and all participant materials will be submitted to the IRB for review and approval. Approval of both the protocol and the consent form(s) will be obtained before any participant is consented. Any amendment to the protocol will require review and approval by the IRB before the changes are implemented to the study. All changes to the consent form(s) will be IRB approved; a determination will be made regarding whether a new consent needs to be obtained from participants who provided consent, using a previously approved consent form.

## INVESTIGATORS' SIGNATURES

The signatures below constitute the approval of this protocol and provides the necessary assurances that this study will be conducted according to all stipulations of the protocol, including all statements regarding confidentiality, and according to local legal and regulatory requirements and applicable US federal regulations and ICH guidelines, as described in the *Statement of Compliance* above.

Principal Investigator or Clinical Site Investigator:

Signed: ______________________________ Date: 1 April 2021

Name: Itai Danovitch, MD

Title: MPI

Investigator Contact Information
Affiliation: Cedars-Sinai Medical Center
Address: 8730 Alden Drive, Suite E-135, Los Angeles, CA 90048
Telephone: 310.423.2600
Email: itai.danovitch@cshs.org

Principal Investigator or Clinical Site Investigator:

Signed: ______________________________ Date: 4/5/21

Name: Allison Ober, MSW, PhD

Title: MPI

Investigator Contact Information
Affiliation: RAND Corporation
Address: 1776 Main Street, Santa Monica, CA 90407
Telephone: 310-393-0411 ext. 6639
Email: ober@rand.org

*Signature of the clinical site investigators who are responsible for the day-to-day study implementation at the specific clinical sites.*

Signed: Date: 1 April 2021

Name: Peter Friedmann, MD

Title: Co-I

Affiliation: Baystate Health

Signed: Date: 29 March 2021

Name: Kimberly Page, MPH, PhD

Title: Co-I

Affiliation: University of New Mexico

# 1 PROTOCOL SUMMARY

## 1.1 SYNOPSIS

| | |
|---|---|
| **Title:** | Collaborative care teams for hospitalized patients with opioid use disorders: Translating evidence into practice |
| **Grant Number:** | 1U01TR002756-01A1 |
| **Study Description:** | The purpose of this study is to evaluate whether an intervention by an interdisciplinary collaborative care team compared with usual care for hospitalized patients with opioid use disorders (OUD) can increase initiation of medication for opioid use disorder (MOUD) and improve linkage to OUD-focused follow-up care. If the aims of the research are achieved, we hope to improve MOUD initiation and linkage to follow-up care as well as clinical outcomes, and, ultimately, create a generalizable, sustainable model of care to increase OUD treatment delivery and decrease the downstream effects of untreated OUD. If effective, this translational model also can be used to increase uptake of evidence-based practices for other substance use and associated behavioral health disorders. |
| **Objectives:** | **Primary Objectives**:<br>1. To test the effectiveness of the START intervention on MOUD initiation relative to usual care.<br>2. To test the effectiveness of the START intervention on linkage with post-discharge OUD treatment relative to usual care.<br>**Secondary Objectives**:<br>1. To test the effectiveness of the START intervention on addiction-focused discharge planning.<br>2. To test the effectiveness of the START intervention on MOUD engagement relative to usual care.<br>3. To test the effectiveness of the START intervention on linkage to medical care relative to usual care.<br>4. To test the effectiveness of the START intervention on self-reported days of opioid use relative to usual care. |
| **Endpoints:** | **Primary Endpoints**:<br>1. Proportion of patients in each arm who initiate MOUD prior to discharge, defined as use of any FDA-approved pharmacotherapy for OUD, including buprenorphine, naltrexone and methadone.<br>2. Proportion of patients in each arm who attend at least one OUD-related care visit within 30 days of hospital discharge.<br>**Secondary Endpoints**:<br>1. Proportion of patients in each arm with an after-hospital care plan that specifies a date and time for a post-discharge addiction care appointment.<br>2. Proportion of patients in each arm who initiate MOUD or continue MOUD treatment within 30 days following hospital discharge. |

| | |
|---|---|
| | 3. Proportion of patients in each arm who complete at least one visit to an outpatient medical provider within 30 days of hospital discharge.<br>4. Days of opioid use in the past 30 days. |
| **Study Population:** | Inpatients* at three medical hospitals in California, Massachusetts, and New Mexico who are 18 or older, admitted for any reason, and screen positive for moderate to severe OUD using the ASSIST |
| **Phase or Stage:** | Pragmatic Clinical Trial |
| **Description of Sites/Facilities Enrolling Participants:** | **Cedars-Sinai Medical Center**: Cedars-Sinai is a nonprofit academic healthcare organization serving the diverse Los Angeles community and beyond. Cedars-Sinai is one of the largest nonprofit academic medical centers in the U.S., with 886 licensed beds, 2,100 physicians, 2,800 nurses and thousands of other healthcare professionals and staff. Cedars-Sinai serves more than 1 million people each year in over 40 locations, with more than 4,500 physicians and nurses and 1,500 research projects in motion.<br><br>**Baystate Medical Center**: Baystate Health (BH) is a not-for-profit, integrated healthcare system serving over 800,000 people in Western New England. Headquartered in Springfield, MA, Baystate Health is comprised of the flagship Baystate Medical Center (713 beds) Baystate Children's Hospital, and three community hospitals: Baystate Franklin Medical Center (89 beds), Baystate Noble Hospital (97 beds), and Baystate Wing Hospital (74 beds). Baystate Health is also comprised of Baystate home care and hospice, Health New England (a local health insurer with approximately 150,000 members), Baystate Reference Laboratories, and Baystate Medical Practices which includes more than 950 primary and specialty care providers spread across more than 85 practice locations throughout Western and Central Massachusetts, including three community health centers. Baystate provides care for approximately 45,000 inpatients, 197,000 emergency care patients, and 1.8 million outpatient visits annually. Participants for this study will be primarily recruited from the inpatient units at Baystate Medical Center. Recruitment may expand to the three community hospitals if needed.<br><br>**University of New Mexico Hospital**: The University of New Mexico (UNM) Health system provides the highest quality of care for more than 200,000 New Mexicans each year. UNM Health providers specialize in over 150 areas of medicine and employ over 7,000 professionals. UNM Health operates more than 30 clinics around the state of New Mexico through the UNM Medical Group and encompasses six hospitals and medical centers. Together, these facilities receive 900,000 outpatient visits, 22,000 surgical cases and 100,0000 emergency room visits each year. Participants in this study |

| | |
|---|---|
| | will be recruited at UNM Hospital, a 550 inpatient, urban, safety net hospital and New Mexico's only Level 1 Trauma Center. |
| **Description of Study Intervention/Experimental Manipulation:** | The intervention is a collaborative care team called the Substance Use Treatment and Recovery Team (START). START is comprised of an Addiction Medicine Specialist (AMS) and a Care Manager (CM). The AMS and CM work closely together to treat patients. The CM assesses readiness, discusses treatment options, assists with linkage to follow-up care, and tracks patient progress in a registry. The AMS assists patients with initiating MOUD, when indicated. The study intervention is initiated in the hospital and lasts a total of 4-6 hours, with various components delivered at different time points during the hospital stay. (Intervention periods must be flexible to work around medical care and patient wellbeing.) The intervention will be conducted in-person or via telehealth (telephone or televideo) depending on COVID-19 protocols. After discharge, the CM will continue the intervention with follow-up calls with patients once a week for one month, or more often if needed, to provide ongoing support and continue to facilitate treatment. Follow-up calls will last approximately 15-20 minutes each. |
| **Study Duration:** | 11 months |
| **Participant Duration:** | Participant will complete study-related tasks over 4-6 hours during the course of their hospitalization. Weekly phone calls of approximately 15-20 minutes each will be conducted for up to one month after discharge. A 30-40 minute 1-month follow-up interview will be conducted. |
| **Research Procedures** | The primary research visits are as follows:<br>• Eligibility screening: A member of the research team conducts eligibility screening with the patient. Responses are entered into the REDCap database.<br>• Consent: A member of the research team provides the consent form to the patient, explains it, and ensures the study is understood.<br>• Baseline interview (30-40 minutes): A member of the research team conducts the baseline interview either in-person, by telephone, or by televideo. Responses are entered into the REDCap database.<br>• 1-month follow-up interview (30-40 minutes): A member of the research team will conduct the follow-up interview by telephone Responses are entered into the REDCap database. |

*The term ”inpatient” throughout this document refers to those either admitted to an inpatient bed or for observation.

## 1.2 SCHEMA

**Figure 1. Flow Diagram**

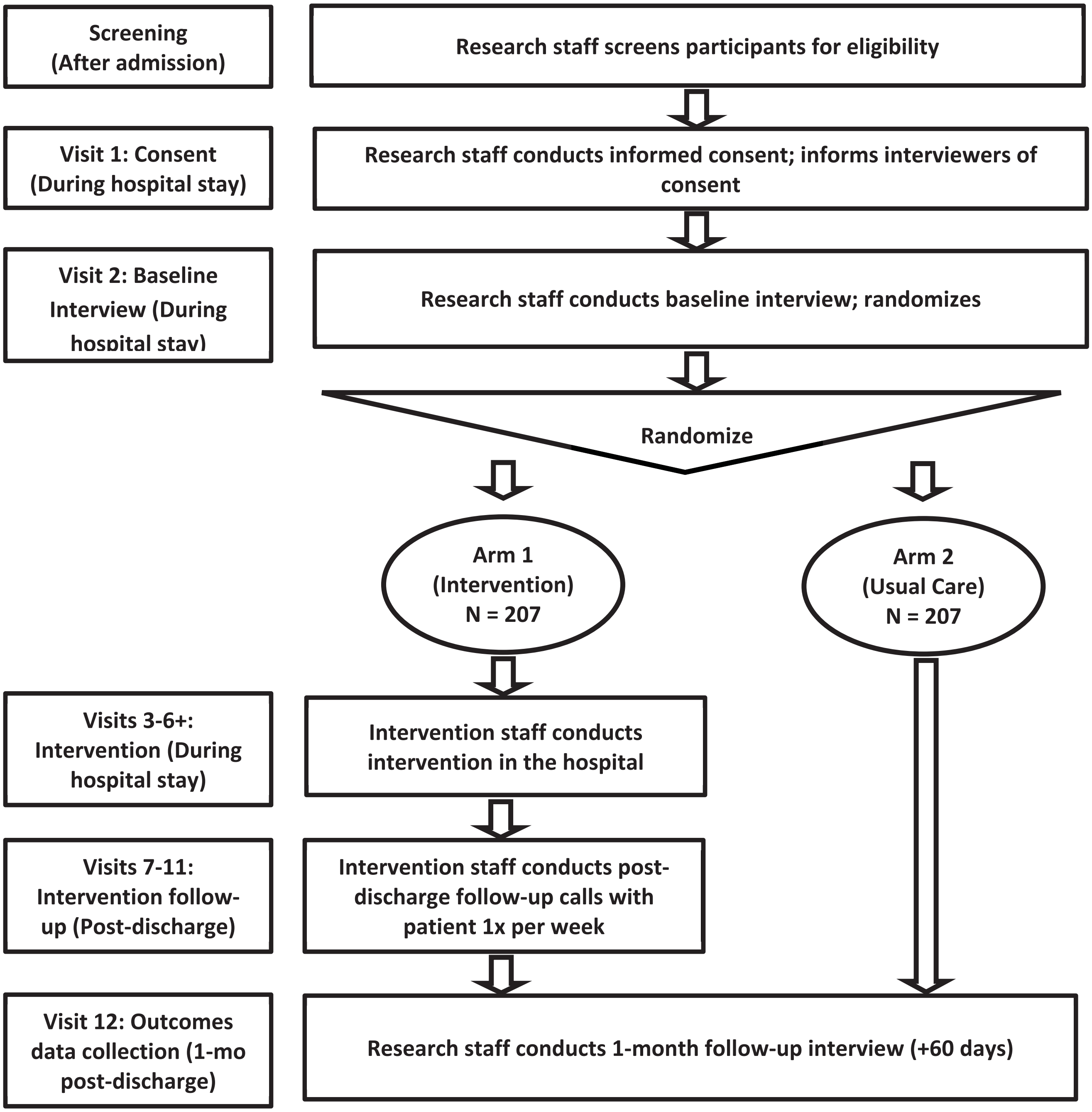

## 1.3 SCHEDULE OF ACTIVITIES

**Table 1. Schedule of Activities**

| | Screening (Pre-consent) | Visit 1: Consent (During hospital stay) | Visit 2: Baseline (During hospital stay) | Visit 3-6+: Intervention (During hospital stay) | Visit 7-11: Intervention Follow-Up (Post-discharge) | Visit 12: Follow-up Interview (30-90 days post- discharge) |
|---|---|---|---|---|---|---|
| ASSIST | X | | | | | |
| Current MAT Utilization | X | | | | | |
| Demographics | X | | | | | |
| Informed Consent | | X | | | | |
| Randomization | | X | | | | |
| Intervention Visits | | | | X | | |
| Post-Discharge Follow-Up Calls | | | | | X | |
| Adverse Events Reporting | | X | X | X | X | X |
| **Outcome Evaluation** | | | | | | |
| Pain Intensity & Frequency (PEG) | | | X | | | X |
| Depression (PHQ-9) | | | X | | | X |
| Anxiety (GAD-7) | | | X | | | X |
| 30-Day Opioid (and other substance) Use (adapted from NSDUH) | | | X | | | X |
| SUD Treatment Utilization (adapted from NSDUH) | | | X | | | X |
| SUD Healthcare and Mental Health Utilization (adapted from GAIN) | | | X | | | X |
| Opinions about MAT | | | X | | | |
| Employment | | | X | | | X |
| Severity of Substance Use (PROMIS) | | | X | | | X |
| Overdoses | | | X | | | X |
| Patient Experience of Stigma | | | X | | | |
| Social Support (MSPSS) | | | X | | | |
| Significant Other with OUD | | | X | | | |
| Criminal Justice Involvement | | | X | | | |
| Demographics | | | X | | | |
| MAT Utilization | | | | | | X |
| Patient Experience of Chronic Illness Care (PACIC) | | | | | | X |
| Therapeutic Alliance (CAHPS)* | | | | | | X |
| Satisfaction with START* | | | | | | X |

*START group only

# 2 INTRODUCTION

## 2.1 STUDY RATIONALE

***Problem/question.*** In the past decade, hospitalizations for OUD nearly doubled, from 301,707 in 2002 to 520,275 in 2012, with inpatient charges for these hospitalizations nearly quadrupling.[1] Patients admitted to the hospital with an underlying OUD rarely receive treatment for the OUD while hospitalized.[2,3] Medication for OUD (MOUD), is rarely initiated in the hospital, and patients are seldom linked to outpatient treatment after discharge.[4] Hospitalized patients with OUD who do not initiate MOUD or receive linkage to post-discharge treatment are at high-risk of continued misuse, delays in care, future overdose and costly readmission.[4-9] This study identifies the inpatient hospital stay as a new opportunity to initiate MOUD and link patients with follow-up care for their OUD. Hospitalization is an opportune time to initiate MOUD and provide linkage to follow-up care for patients with an OUD.[10]

***Rationale for the clinical trial and intervention.*** Collaborative care is a team-based treatment approach that uses multi-faceted interventions (care management, pharmacotherapy and psychotherapy), measurement-based care, and patient registries to increase delivery of evidence-based care. A collaborative care team (CCT) in the hospital offers specialist expertise, an organized system of care, and opportunities for patient education. A hospital-based CCT has the potential to facilitate initiation of MOUD during the inpatient stay and linkage to post-discharge care.[11] Prior studies have demonstrated the effectiveness of collaborative care in outpatient settings for patients with opioid and alcohol use disorders.[12-16] To our knowledge, however, no experimental studies to date have focused on testing a hospital-based collaborative care team to help patients initiate MOUD and to provide focused discharge planning and follow-up monitoring for hospitalized patients with an OUD. Our study is a multi-site, randomized trial at three diverse sites to evaluate whether a CCT, called the Substance Use Treatment and Recovery Team (START), increases the use of evidence-based interventions for OUD (i.e., MOUD and OUD-focused discharge planning) and improves linkage to follow-up care among hospitalized patients with OUD.

## 2.2 BACKGROUND

***The underuse of effective treatments for behavioral health conditions in the hospital setting is an important translational problem.***[17] Patients with behavioral health conditions are often hospitalized for emergent medical and surgical treatment,[18-23] and the clinical conditions that prompt hospitalization often are related to undertreatment of the behavioral health condition.[24] However, despite the prevalence of co-morbidity, few patients receive evidence-based treatments, or are successfully linked to treatment after discharge.[2-4,25] This has critical consequences: patients with mental health and substance use comorbidities have longer length of stay, higher readmission rates, and worse

outcomes,[4,6,8,26] and the inpatient hospitalization becomes a missed opportunity to address suffering and long-term health care costs.[20,27]

***There are multiple scientific and operational reasons for this translational science problem.*** Inpatient physicians frequently treat acute overdose, withdrawal, and suicidality, but they lack knowledge and skills for addressing behavioral health disorders, such as how to choose and adjust medication therapy, or when to refer for psychotherapy.[28] Given pressures to minimize length of stay, the inpatient team is primarily focused on addressing the acute reason for admission and may defer addressing chronic conditions to outpatient providers. Moreover, few hospitals have the organizational infrastructure needed to treat behavioral health conditions effectively, such as dedicated teams, evidence-based protocols, or the ability to coordinate care transitions, such that patients can be linked to community resources.[29] Separate funding streams, stringent federal privacy regulations, and long waiting lists for publicly-funded treatment, create additional barriers.[30,31] Finally, patients with behavioral health conditions often experience stigma, even from health care professionals, a known barrier to treatment.[32,33]

***Opioid use disorders (OUD) are an exemplar of this problem.***[31,34] Patients with OUD experience high burden of disease from medical comorbidities,[35] and are increasingly hospitalized with medical complications related to OUD.[1,7] In the past decade, hospitalizations for OUD nearly doubled, from 301,707 in 2002 to 520,275 in 2012, with inpatient charges for these hospitalizations nearly quadrupling.[1] Although treatment for OUD is highly effective,[36-38] patients admitted to the hospital with an underlying OUD rarely receive treatment for the root cause of their hospitalization—the OUD[2,3] – or are linked with outpatient treatment.[4,39] High rates of discharges against medical advice (~15%) suggest failed care transitions.[9] While the acute manifestations of an OUD (such as opioid intoxication and withdrawal) may be addressed, effective treatment for the underlying disease itself, medication for OUD (MOUD), is rarely initiated during the inpatient stay, and patients are seldom linked to post-discharge treatment.[4] This missed opportunity leaves patients at high-risk of continued misuse, delays in care, future overdose and costly readmission.[4-9]

***Starting treatment in the hospital and linking patients with post-discharge care addresses the treatment gap and could lower readmissions and costs.*** Despite being in the midst of an opioid epidemic of unprecedented scale, in 2015 only 20% of those with a drug use problem received any treatment.[40,41] And, among Medicaid enrollees, only 25% of those hospitalized with a substance use disorder were linked with post-discharge treatment.[4] MOUD (buprenorphine/naloxone, methadone and injectable naltrexone) cuts overdose rates, reduces criminal behavior and infectious disease, and lowers mortality.[36,37,42] When started in the hospital, MOUD increases outpatient treatment and reduces opioid use. Among hospitalized Medicaid enrollees with substance use disorders, patients who were linked to care within 14 days were less likely to be readmitted.[4] Studies suggest that the inpatient hospitalization is a teachable moment, and that patients are willing to engage with treatment, if barriers can be reduced.[43-47] Rapid-access pathways to treatment after discharge address systemic problems with care transitions and low community capacity.

***Inpatient health care providers face structural and functional barriers to the delivery of coordinated, evidence-based treatment for OUD.*** Within the hospital, efficiency pressures to reduce costs drive hospitalist services to stabilize acute symptoms and defer treatment of OUD to the ambulatory setting. Most providers in hospital settings are not trained to assess or manage patients with OUD, contributing to low rates of OUD identification or treatment initiation.[28] Patient ambivalence requires targeted motivational interventions, which hospitalists are not typically trained to provide. Stigmatization of OUD and untreated withdrawal may contribute to high rates of discharge against medical advice (~15%), which can truncate treatment for the condition that prompted the hospitalization.[7,48,49] Barriers impeding linkage to post-discharge OUD care include the lack of outpatient providers (which requires persistence to find an opening for treatment) and federal privacy regulations that effectively segregate medical and substance use care.[30,31] Insurance coverage for addiction aftercare services is frequently inadequate, and even insurers with benefits may not have an easily accessible network of community providers. Further, medical comorbidities may limit patient aftercare options.

***There is a debate about how best to increase access to behavioral health treatment in the hospital.***[29] There are three service delivery models that have been used to address the behavioral health conditions of hospitalized patients; all are limited in that all of them are provided by a single individual and are focused on addressing the acute problem. In the traditional consultation-liaison psychiatry model, a primary medical or surgical team requests a consultation for a recognized problem and is responsible for implementing the consultant's recommendations. In the proactive model,[50,51] case-finding strategies identify patients early in their hospitalization and the consultant provides recommendations to the primary team. The co-management model[52] is characterized by an embedded behavioral health provider on a medical or surgical service, who has a direct role in case finding and management, rather than standard consultative recommendation. We chose to test a collaborative care model because it addresses the limitations of the three previous models. Most service delivery models[51,52] used to address behavioral health conditions among hospitalized patients are limited by being implemented by a single individual. A team-based approach can capitalize on the different skills available from team members, execute tasks more efficiently, and address a range of patient needs.[53] Recently, a team-based model was experimentally tested for patients with co-morbid trauma and alcohol abuse, with the goal of preventing PTSD,[54-58] but treatment was not provided until 3 months after discharge. While hospitals have started to develop addiction consultation services—suggesting feasibility and readiness—research on these services has been observational rather than experimental.[5,11,43,46,59,60] The single study with a randomized design occurred at one site and was a buprenorphine effectiveness study rather than a translational study.[61] Given ongoing pressures to demonstrate the value of service delivery interventions, there is a pressing need to experimentally test whether an inpatient collaborative care team (CCT) approach to increasing treatment delivery can improve outcomes.

***There is a strong scientific premise for testing the effectiveness of an interdisciplinary, collaborative care team (CCT) as a translational approach to the problem of OUD treatment underuse.*** Substantial evidence supports the use of collaborative care for behavioral health disorders,[62] and for behavioral health conditions co-morbid with medical illnesses.[11,63-65] Significantly, collaborative care programs are

highly effective for safety-net patients and can reduce health disparities in access.[66-71] Work by our team demonstrated the effectiveness of collaborative care in outpatient settings for patients with opioid and alcohol use disorders.[12-16] Collaborative care is a service delivery approach that uses multi-faceted interventions (care management, pharmacotherapy and psychotherapy), clinical measures, systematic patient registries, and a team orientation to increase delivery of evidence-based care. By offering expertise that most hospital-based physicians lack, creating an organized system of care, providing patient education with a focus on self-management and harm reduction (e.g., giving patients prescriptions for naloxone), and addressing barriers to follow-up care, a hospital-based CCT could overcome the translational roadblocks to initiating MOUD during the inpatient stay and linking patients with post-discharge care.[11] If CCTs work, they could be a new and highly significant translational approach to addressing the problem of OUD treatment underuse.

***The study offers a new model—a consultation service-based collaborative care team—for improving care processes for hospitalized patients with OUD.*** Hospitals have extensive experience using care managers to improve in-hospital and follow-up care for several patient populations at high risk of readmission,[72,73] including acute medical patients,[74] and many have a consultation service to support the medical team with patients in need of behavioral health care. However, to date, we have not seen any cross-site experimental studies of collaborative care models to improve outcomes for inpatients with OUD. Further, leveraging the existing consultation service is an innovative and generalizable approach to managing the large number of hospital inpatients with untreated OUD without burdening inpatient physicians and unit case managers who may not have the expertise or time to prescribe medications or resolve barriers to OUD-focused discharge and follow-up. The consultation service-based CCT is a novel, comprehensive program for facilitating MOUD initiation in the hospital and linking patients to follow-up care for one of the most common SUDs among inpatients.

Our study is a multi-site, randomized pragmatic trial in being conducted in three diverse sites. The study, called the Substance Use Treatment and Recovery Team (START), will evaluate whether a CCT increases the use of two interventions—MOUD with buprenorphine, methadone or injectable naltrexone, and OUD-focused discharge planning—among hospitalized patients with OUD and improves linkage to follow-up care relative to usual care. The START consists of an addiction medicine specialist and a care manager who will use evidence-based tools to decrease barriers to MOUD and engage patients with post-discharge OUD care. We will randomize 414 patients total from Cedars-Sinai Medical Center in Los Angeles, the University of New Mexico Hospital in Albuquerque, and Baystate Health in Springfield, Massachusetts to receive either START or usual care, stratifying by prior MOUD exposure.

***This project has potential for high impact because it both improves public health and advances translational science.*** Our study simultaneously addresses a critical public health problem – the opioid crisis – and advances translational science. The undertreatment of OUD is arguably the most important translational science problem related to the opioid crisis. In 2015, 11.5 million individuals reported misusing opioids and 1.9 million reported being addicted to opioids,[75] yet fewer than 20% receive any treatment.[40,41] By experimentally testing a new translational approach – the CCT on treatment translation – and assessing implementation factors such as context and cost, this study could both

improve public health by identifying an efficient and generalizable model to increase OUD treatment delivery and decrease the downstream effects of untreated OUD, and it could also advance translational science by identifying an effective and generalizable approach to address translational roadblocks that result in the undertreatment of behavioral health conditions. Our findings on cost and sustainability will be highly relevant to hospital administrators and policy makers considering operational and financial implications of adopting and implementing similar services.

The study builds from a small pilot RCT (N=80) now being conducted at Cedars-Sinai by testing the intervention at three diverse locations, thus increasing generalizability. We also examine whether and how context influences outcomes. By addressing context, cost and sustainability along with a diverse range of traditional patient-level outcomes, we go beyond the usual scope of clinical trials. Diffusion of innovation theory emphasizes that factors related to context influence implementation.[76] This innovative approach to study design can generate more rapid translational gains, more effective downstream implementation and dissemination strategies, and more practical information for decision-makers.

## 2.3 RISK/BENEFIT ASSESSMENT

### 2.3.1 KNOWN POTENTIAL RISKS

The study procedures involve the following potential risks: (1) unauthorized disclosures of sensitive information, (2) psychological distress, and (3) financial costs associated with treatment. See Protection of Human Subjects.

1. Disclosures of Sensitive Information: Potential risks include transmission of protected health information without using encryption, without password protection, or to an unintended member of the research team, and transmission of protected health information outside of the study team. A breach of confidentiality could cause psychological or other harm to patients due to disclosure of sensitive study information.
2. Psychological Distress: Patients could experience psychological distress from discussing their substance use and mental health during data collection or the intervention.
3. Financial Costs of Treatment: Patients could experience distress over costs of treatment.

***Note that patients could experience some expected side effects of OUD medication, however, this study is not testing the efficacy of medications. Medications, which are optional, are FDA-approved and considered the standard of care for treatment of OUD.***

### 2.3.2 KNOWN POTENTIAL BENEFITS

The study may benefit individual participants by helping participants receive evidence-based FDA approved OUD medication as well as behavioral treatment while hospitalized and after discharge. The study will also have long-term benefits for society by improving public health and advancing translational science. This study will provide several types of new knowledge:

1. Whether START compared with usual care improves care for hospitalized patients with an OUD. That is, whether START leads to improved initiation of medication and linkage to follow-up care for patients who are admitted to the hospital, either for a problem related to their OUD or for another medical issue.
2. Whether START compared with usual care reduces substance use.
3. Whether START, implemented in hospitals with different contexts, is cost-effective and is sustainable.

These knowledge gains will provide invaluable, preliminary information on how to improve the quality of care for hospitalized patients with OUD and on how to address an unmet need that has severe individual and societal consequences.

### 2.3.3 ASSESSMENT OF POTENTIAL RISKS AND BENEFITS

Because the purpose of this study is to increase the use of FDA-approved, evidence-based treatments for OUD, for each individual patient enrolled, the potential benefits of participation are likely to outweigh risks. Untreated OUD puts patients at risk for fatal and non-fatal overdose as well as other acute and chronic harms. In addition, the study is intended to inform hospitals' and national policymakers' responses to the current crisis in the overuse of opioid medications, with its attendant high fatality rates. Thus, the research offers substantial potential benefit to both subjects and society with little risk to patients.

We will minimize risks in the following ways:

*Disclosure of Sensitive Information*: We will protect against unauthorized disclosures of information through a robust data safeguarding plan. Site PIs will be responsible for serving as local data safeguarding officers, assuring adherence to the study data safeguarding plan and training in human subjects protections. Standard procedures will include storing data on secure institutional password-protected servers, using password-protection and encryption when transmitting any data (including within a hospital), limiting access to patient identifiers to the smallest number of individuals possible, ensuring that datasets do not have sensitive information in them unless necessary, assigning patients study ID numbers at random to enable removal of other direct HIPAA identifiers from datasets, and ensuring that all individuals who handle study data and study staff are trained in human subjects protections, HIPAA, and study procedures. Individuals who handle data on potential or actual study subjects will be required to avoid any unplanned disclosures of information beyond the study team, and will be required to report any unplanned disclosures.

Per Section 2012 of the 21st Century Cures Act as implemented in the 2017 NIH Certificates of Confidentiality Policy, all ongoing or new research funded by NIH as of December 13, 2016 that is collecting or using identifiable, sensitive information is automatically issued a Certificate of Confidentiality. Researchers with a Certificate of Confidentiality may ONLY disclose identifiable, sensitive information in the following circumstances: if required by other Federal, State, or local laws, such as for

reporting of communicable diseases; if the subject consents; or for the purposes of scientific research that is compliant with human subjects regulations.

*Psychological Distress*: To reduce the potential for psychological risks, study coordinators and members of the START will undergo training to ensure that they approach patients in a manner that is empathetic and non-judgmental, based on principles of motivational interviewing. If patients exhibit mild to moderate discomfort with study eligibility or survey questions, the study coordinator will ask the patient whether he/she prefers to complete the activity later. If patients exhibit severe distress, this will be interpreted as an inability to consent. If a patient volunteers that they are having thoughts about harming themselves or others, they will be referred to a psychiatrist immediately. If the participant reports any thoughts of suicide during the baseline interview (i.e. scored anything other than never—1,2, 3, DK, REF—on MH1i), upon completion of the interview the interviewer will inform the site PI or Co-I, who will ensure that a physician on the primary team is aware and can address in accordance with their standard of care as indicated. If the participant reports an thoughts of suicide during the follow-up interview (i.e. scored anything other than never—1,2, 3, DK, REF—on MH1i), the CSSRS (see below) will be administered in REDCap. If the patient is at high-risk of suicide per the CSSRS, the interviewer will address this upon completion of the interview. The interviewer will refer the patient to a suicide hotline and will inform the study clinician (site PI/Co-I or other). If the participant appears to be experiencing emotional distress during the baseline interview, the interviewer will ask the participant if they would like the interviewer to make the primary team aware. If the participant appears to be experiencing emotional distress during the follow-up interview, the interviewer will ask the participant if they would like to receive support after the call. If so, upon completion of the interview the interviewer will provide the number of local mental health resources. Study hospitals have on-staff psychiatrists available at all times in case of psychiatric emergencies.

*Adverse Effects of Treatment*: The Addiction Medicine Specialist on the START will adhere to clinical standards of care for the field. S/he will also adhere to the following the clinical protocols used in the study related to initiating, adjusting, discontinuing, or otherwise changing therapies including medication. Medications will be monitored clinically, particularly during dose adjustments. If patients report any adverse effects of treatment to study staff, these will be immediately reported to the treating providers who ordered the treatment. For example, if a patient tells a care manager about medication side effects, then the care manager would immediately refer this information to the treating physician. The risks of treatment with MOUD are similar to but lower than those associated with untreated OUD.

*Financial Costs to Patients*: The study aims to test whether the START intervention facilitates linkage to treatment. This could result in financial cost to patients who engage in treatment. As part of the intervention group (START), the care manager will work with patients to try to minimize the costs of any medications and follow-up care by helping the patients to understand any insurance benefits that they may have and to access available community resources. Usual care patients could receive assistance in minimizing the costs of medications and follow-up care as part of regular discharge planning by the medical team.

# 3 OBJECTIVES AND ENDPOINTS

**Table 2. Objectives and Endpoints**

| OBJECTIVES | ENDPOINTS | JUSTIFICATION FOR ENDPOINTS | PUTATIVE MECHANISMS OF ACTION |
|---|---|---|---|
| Primary | | | |
| To test the effectiveness of the START intervention on MOUD initiation relative to usual care | Proportion of patients in each arm who initiate MOUD prior to discharge, defined as use of any FDA-approved pharmacotherapy for OUD, including buprenorphine, naltrexone and methadone. | Compared to usual care, START will result in a higher proportion of patients initiating MOUD for OUD in the hospital. | Improved understanding of and motivation for MOUD through addiction focused motivational interviewing and discharge planning |
| To test the effectiveness of the START intervention on linkage with post-discharge OUD treatment relative to usual care | Proportion of patients in each arm who attend at least one OUD-related care visit within 30 days of hospital discharge. | Compared to usual care, patients in the START arm will have increased linkage to post-discharge OUD treatment. | Improved understanding of and motivation to seek post-discharge care |
| Secondary | | | |
| To test the effectiveness of the START intervention on addiction-focused discharge planning | Proportion of patients in each arm with an after-hospital care plan that specifies a date and time for a post-discharge addiction care appointment. | Compared to usual care, START will result in a higher proportion of patients having a post-discharge care appointment. | Facilitated discharge planning |
| To test the effectiveness of the START intervention on any post-discharge MOUD utilization relative to usual care | Proportion of patients in each arm who initiate MOUD or continue MOUD treatment within 30 days of hospital discharge. | Compared to usual care, START will result in a higher proportion of patients engaging in MOUD treatment after the hospital. | Improved understanding of and motivation to seek post-discharge care |
| To test the effectiveness of the START intervention on linkage to medical care relative to usual care | Proportion of patients in each arm who complete at least one visit to an outpatient medical provider within 30 days of hospital discharge. | Compared to usual care, START will result in a higher proportion of patients linking to medical care after the hospital. | Improved understanding of and motivation to seek post-discharge care |
| To test the effectiveness of the START intervention on self- | Days of opioid use in the past 30 days | Compared to usual care, START will result in a higher | Reduced cravings for opioids |

| OBJECTIVES | ENDPOINTS | JUSTIFICATION FOR ENDPOINTS | PUTATIVE MECHANISMS OF ACTION |
|---|---|---|---|
| reported days of opioid use relative to usual care | | proportion of patients with reduced opioid use. | |

# 4 STUDY DESIGN

## 4.1 OVERALL DESIGN

**Design**

This study is a pragmatic randomized controlled trial (RCT) to evaluate primarily whether the START intervention compared to usual care increases initiation of medication for OUD in the hospital—MOUD with buprenorphine, methadone or injectable naltrexone, linkage to OUD care post-discharge—among hospitalized patients with OUD. The study also will evaluate the effect of the START intervention on post-discharge planning, linkage with or continuation of MOUD, utilization of medical care and reduction in opioid use after discharge.

The START intervention consists of an addiction medicine specialist and a care manager who will use evidence-based tools such as motivational interviewing and addiction-focused discharge planning to decrease barriers to MOUD and engage patients with post-discharge OUD care.

The trial will be implemented at Cedars-Sinai Medical Center (CSMC) in Los Angeles, the University of New Mexico Hospital in Albuquerque, and Baystate Health in Springfield, Massachusetts. Patients will be randomly assigned within each hospital to receive either START or usual care, stratifying by prior MOUD exposure.

**Figure 2. Consort Diagram (Anticipated)**

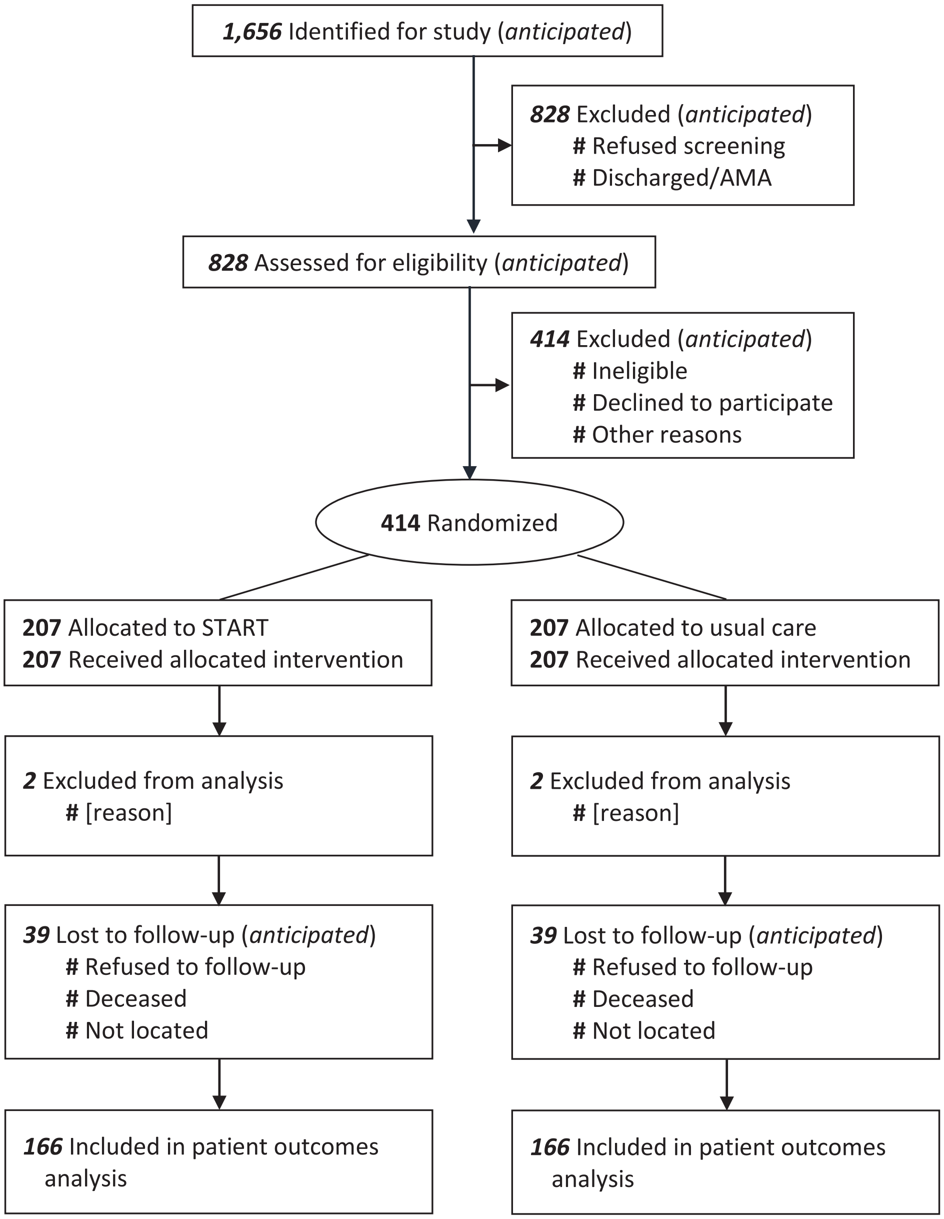


**Hypotheses**

The primary objectives/hypotheses are:

1. To test the effectiveness of the START intervention on MOUD initiation relative to usual care.
    a. Hypothesis: Compared to usual care, START will result in a higher proportion of patients initiating MOUD in the hospital.

2. To test the effectiveness of the START intervention on linkage with post-discharge OUD treatment relative to usual care.

    a. Hypothesis: Compared to usual care, a higher proportion of patients in the START arm will have increased linkage to post-discharge OUD treatment.

The secondary objectives/hypotheses are:

1. To test the effectiveness of the START intervention on addiction-focused discharge planning
    a. Hypothesis: Compared to usual care, START will result in a higher proportion of patients having a post-discharge care appointment.

2. To test the effectiveness of the START intervention on any post-discharge MOUD utilization relative to usual care.

    a. Hypothesis: Compared to usual care, START will result in a higher proportion of patients initiating MOUD or continuing MOUD treatment.

3. To test the effectiveness of the START intervention on linkage to medical care relative to usual care.

    a. Hypothesis: Compared to usual care, START will result in a higher proportion of patients linking to medical care after the hospital.

4. To test the effectiveness of the START intervention on self-reported days of opioid use relative to usual care.

    a. Hypothesis: Compared to usual care, START will result in a higher proportion of patients with reduced opioid use.

**Phase**

N/A - This is a pragmatic clinical trial.

## 4.2 SCIENTIFIC RATIONALE FOR STUDY DESIGN

The study is a randomized controlled trial testing the START intervention compared with usual care. Usual care at each hospital consists of the medical team referring patients suspected of having an OUD to a psychiatric consultation service with an AMS or directly to an AMS not affiliated with a consultation service. None of the hospitals is currently employing as usual care a collaborative care team that consists of an AMS – CM team that uses a set of principles based on collaborative care along with evidence-based tools to support the medical team in intervening with patients with OUD and delivering for OUD treatment in the hospital and after discharge. This pragmatic trial intends to measure the START intervention's effectiveness compared with usual care in diverse hospital settings. The RCT design allows for this comparison.

## 4.3 JUSTIFICATION FOR INTERVENTION

Prior research has shown collaborative care to be an effective model to treat behavioral health disorders,[62] behavioral health conditions co-morbid with medical illnesses,[11,63-65] and substance use disorders.[12-16] Collaborative care can also reduce health disparities in access.[66-71] However, this model has not been tested experimentally for hospitalized inpatients with OUD. Core components of collaborative care consist of a collaboration between a behavioral health specialist (care manager) and AMS team, the team using evidence-based practices and tools, close collaboration between the AMS and CM, and follow-up with the patient.[77] Literature on dosage and core components of collaborative care in the hospital setting is not yet available, but we have defined fidelity as incorporating elements of each core component. Thus, we do not set values on the dosage of the intervention. Rather, we define the intervention as interacting with the AMS and CM team, the AMS and CM incorporating evidence-based tools (brief negotiated interview and addiction-focused discharge planning) and the patient receiving follow-up care. Common in pragmatic trials, the study uses an "intent-to-treat" design, thus all participants randomized to receive the START intervention will be included in the evaluation regardless of whether they receive the intervention. We anticipate it will be rare for participants to receive none of the intervention components. As an exploratory analysis, we will incorporate dosage (number of visits with the START) into our statistical models to understand the relationship between amount of intervention received and intervention effects.

## 4.4 END-OF-STUDY DEFINITION

A participant is considered to have completed the study if he or she has completed the baseline and 1-month follow-up assessments. Ideally, the participant will also receive some or all of the intervention but due to the dynamic nature of the hospital stay (unpredictable medical treatments and discharge, leaving against medical advice) and post-discharge complexities, participants may not be able to receive the whole intervention. Nevertheless, the study is "intent to treat," so all participants in the intervention condition will be included in the analysis.

# 5 STUDY POPULATION

## 5.1 INCLUSION CRITERIA

In order to be eligible to participate in this study, an individual must meet all of the following criteria:

1. Admitted to an inpatient bed or for observation at Cedars-Sinai Medical Center (CSMC), University of New Mexico Hospital (UNM), or Baystate Health (BH)
2. Age 18 and older
3. Have a probable OUD diagnosis, defined by scores of >3 on the opioid section of the Alcohol, Smoking, and Substance Involvement Screening test (ASSIST)
4. Speaks English or Spanish as primary language
5. Able to provide informed consent

## 5.2 EXCLUSION CRITERIA

An individual who meets any of the following criteria will be excluded from participation in this study:

1. Currently receiving FDA-approved medication treatment for an opioid use disorder*
2. <6 months life expectancy

*"Currently receiving medication" is defined as medications on order at the hospital or soon to be ordered while in the hospital, as indicated by medication orders in the EMR, or by patient self-report on the eligibility screener of taking the medication since their admission or being told by their medical team that they will be receiving the medication while in the hospital.

## 5.3 LIFESTYLE CONSIDERATIONS

N/A

## 5.4 SCREEN FAILURES

Screen failures are defined as participants who do not meet eligibility criteria for participation in this trial or who consent to participate in this study but are not subsequently assigned to the study intervention (i.e., they are discharged from the hospital or leave against medical advice before completing the baseline interview and/or randomization).

Individuals who do not meet the criteria for participation in this trial (screen failure) because of meeting one or more exclusion criteria that are likely to change over time may be rescreened. Examples include if someone reenters the hospital and now meets criteria for an opioid use disorder or is no longer on an FDA-approved medication for opioid use disorder, or was discharged or left AMA before being randomized. Rescreened participants will be a new participant number as for the initial screening.

## 5.5 STRATEGIES FOR RECRUITMENT AND RETENTION

### 5.5.1 PATIENT IDENTIFICATION AND RECRUITMENT

1. Approved study staff will prescreen patients for screening and potential enrollment in two ways:
   - A daily electronic medical record (EMR) report of opioid misuse ("Daily Report") that lists potentially eligible subjects (variables include demographics, opioid history, diagnoses, and screenings)
   - Clinician referral
2. Approved study staff will approach patients in two ways:
   - CSMC Only: Potential subjects will be contacted directly by approved study staff for this research through the Direct-to-Patient Recruitment Letter. A courtesy notification will

  - be sent to subjects' treating physicians to notify them of the invitation to participate whenever feasible.
  - Baystate and UNM: If the attending physician agrees, the physician, someone from the medical team or designated study staff will explain the study to the patient.
3. The study team will use the following advertising and recruitment materials (Appendix 10.1):
   For physicians:
   - Study Physician Flyer
   - Physician Screening Card
   - Physician Recruitment Letter (Dear Doctor Letter), accompanied by FAQs about START

   For patients:
   - Baystate and UNM: Physician to Patient Letter (Dear Patient Letter) to be provided to patients deemed eligible for the study.
   - CSMC: Direct-to-Patient Recruitment Letter (Dear Patient Letter) to be provided by study staff to patients deemed eligible for the study.

### 5.5.2 CONSENT AND ENROLLMENT

1. Approved study staff will provide the full consent form and review the consent summary (Appendix 10.1) with the patient.
2. Consent will be obtained in-person either on paper or electronically using an approved platform or remotely using an approved platform.
3. When signing electronically, patients will be offered the option of receiving a copy of the signed and dated consent form by encrypted email. Otherwise, they will be given a paper copy.
4. Approved study staff will enroll consented, eligible patients by assigning a study ID in the REDCap project.
5. All patients will be given patient education materials on opioid use disorder, as well as harm reduction materials (Appendix 10.1). If the visit is remote, the research team will give these materials to a member of the medical team to deliver to the patient or place them in the patient's room.

*5.5.1 – 5.5.2 are shown in Figure 3.*

### 5.5.3 RETENTION

Retention during the hospital stay is not expected to be problematic. To improve post-discharge retention, at the time of enrollment, study staff will collect multiple forms of contact information from participants to increase the chances that we will be able to reach participants after discharge even if there are changes in contact information (e.g., new phone number). Participants without a means of being contacted during the follow-up period will be provided a pre-paid phone with minutes by the study.

### 5.5.4 INCENTIVES

Participants will receive a $50 incentive after completing the baseline interview and a $50 incentive after completing the follow-up interview. Participants who initiate contact with the study team either to schedule a follow-up interview or to provide updated contact information, will receive an additional $5 incentive after completing the follow-up interview. Incentive type (i.e., gift/merchandise card, cash) and delivery method will follow standard site-specific procedures.

### 5.5.5 ANTICIPATED ENROLLMENT

Enrollment will be conducted at three hospitals: Cedars-Sinai Medical Center in Los Angeles, CA; Baystate Health in Springfield, MA; University of New Mexico Hospital, Albuquerque, NM.

**Table 3. Screening and Potential Enrollment**

| | |
|---|---|
| Average Monthly OUD pts to be prescreened<br>(CSMC 1023; Bay State 1433; UNM 2766) | 5222 |
| Total possible per month to screen | 435 |
| Total possible per month to be screened if recruiting 4.5 days/week | 261 |
| Potential enrollment per month assuming 25% eligible/consenting | 65 (~41.4 needed) |
| **Total to be enrolled (41.4 X 10 months)** | **414** |

**Table 4. Projected Enrollment by Site, 10-months**

| | | | |
|---|---|---|---|
| CSMC | CC | 69 | 138 |
| | UC | 69 | |
| Baystate | CC | 69 | 138 |
| | UC | 69 | |
| UNM | CC | 69 | 138 |
| | UC | 69 | |
| | | **Total to be enrolled** | **414** |

**Figure 3. RCT Recruitment, Enrollment, Randomization**

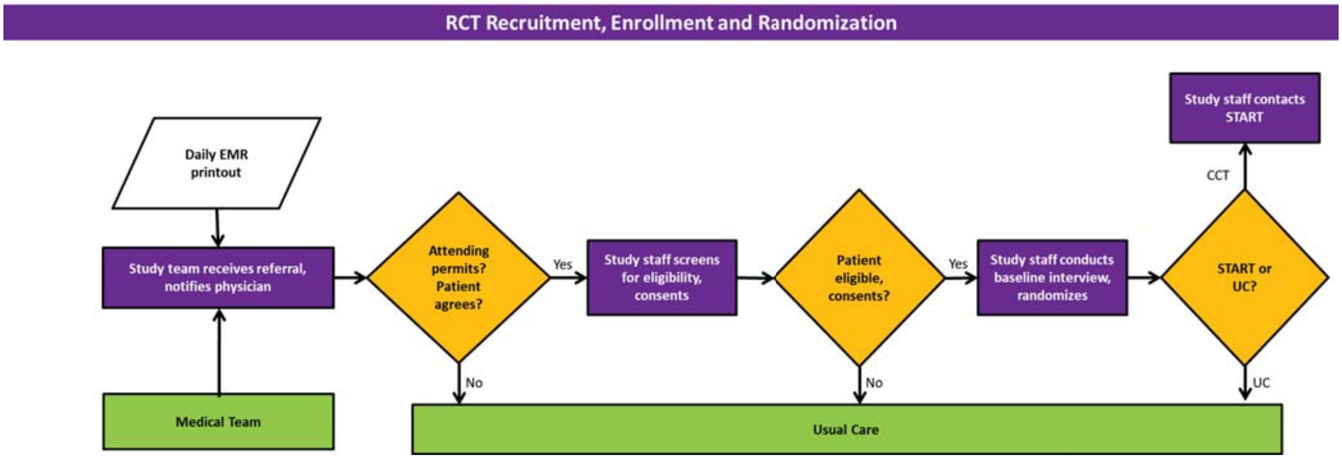

# 6 STUDY INTERVENTIONS

## 6.1 STUDY INTERVENTION ADMINISTRATION

### 6.1.1 STUDY INTERVENTION DESCRIPTION

The components of the START intervention are as follows (see the START Intervention Manual for details):

1. Triage (Care Manager [CM] and Addiction Medicine Specialist [AMS]):
   - The CM assesses the patient for acute biomedical needs related to the OUD. The AMS addresses acute biomedical needs (e.g. facilitates withdrawal management).
2. Engage, assess, plan (CM and AMS) (conducted in person or remotely based on circumstances):
3. If there is not an urgent need for medical intervention or after the urgent medical need is addressed, the CM and/or AMS:
   - engages with the patient (CM and AMS)
   - conducts a diagnostic and biopsychosocial assessment (CM)
   - conducts a complete biomedical assessment and addresses comorbidities (AMS)
   - delivers the START intervention, consisting of elements of the brief negotiated interview (BNI) and re-engineered discharge (RED) – two evidence-based interventions to assess and increase readiness – and develops a plan for initiating evidence-based treatment for OUD (MOUD and/or psychosocial treatment) during and after the hospital stay (CM)
   - ensures the patient understands the follow-up plan and addresses barriers (CM).
4. Treat (AMS):
   - The AMS facilitates MOUD initiation.
   - The AMS facilitates psychosocial treatment for the substance use disorder, if indicated and available.
5. Communicate and Coordinate (CM, AMS)
   - The CM and AMS communicate with each other to continue care through one-month (or more) after the patient is discharged.
   - The CM and AMS communicate with the patient and medical team, and, when appropriate, the patient's family and outpatient providers.
6. Follow-up (CM):
   - The CM calls the patient at least once a week for 1 month after the patient is discharged from the hospital to assess whether the patient is following through with the discharge plan.
   - The CM may also call outpatient providers to determine if the patient linked to care and has encountered barriers.
7. Monitor (AMS):

- The AMS continues to monitor the patient after discharge through the CM's follow-up work.

### 6.1.2 USUAL CARE DESCRIPTION

Usual care consists of each hospital's current practices for managing patients identified with OUD along with each patient enrolled in the study receiving MOUD education and referral information. None of the hospitals is currently employing a collaborative care team that consists of an AMS – CM team that uses a set of principles based on collaborative care along with evidence-based tools to support the medical team in intervening with patients with OUD and delivering OUD treatment in the hospital and after discharge. The AMS at all three hospitals will serve as the AMS for the START study and will not see usual care patients. Usual care at each hospital is described below.

At CSMC, patients randomized to the UC study condition may receive a referral to the existing consultation liaison (CL) service if the patient's medical team determines the need for a consult, or they will be treated and provided discharge planning directly by the medical team. The CSMC CL service has several psychiatrists and social workers who can discuss opioid use with the patient and help the patient initiate medication, if indicated. These providers can also provide consultation to the medical team on whether medication initiation in the hospital and treatment after discharge are indicated. If the START AMS or CM receive a referral from a medical team, they will check the electronic medical record to see if the patient is in the study and their study condition. If the patient is in the UC condition, they will pass the referral to another psychiatrist and/or CM. The study AMS and CM will not take any referrals for UC patients for the duration of the study. If directly approached by a member of the medical team for consultation, the study AMS or CM will refer them to **California Bridge Program resources.**

At Baystate, patients randomized to the UC study condition can be treated directly with MOUD and provided discharge planning by the medical team. If the START AMS or CM receives a referral from a medical team, they will check the electronic medical record to see if the patient is in the study and their study condition. If the patient is in the UC condition, they will refer the referring physician to **California Bridge Program resources.** For patients in the UC study condition, the referring physician will have the option to contact the standard Psychiatric Liaison Consult Service, which does not include an AMS or CM.

At UNM, patients randomized to the UC study condition can be treated directly with MOUD and provided discharge planning by the medical team. If the AMS is contacted for a consult about a patient in the UC condition, the AMS will provide the medical team with an existing wiki page link and/or **California Bridge Program resources**. When the AMS receives a referral, they will check a study-staff maintained registry to determine if referred patients are in the study and their study condition.

### 6.1.3 ADMINISTRATION AND/OR DOSING

The CM and AMS may meet with the participant as frequently as necessary to provide OUD treatment while the patient is hospitalized. The anticipated in-hospital duration of the intervention is 4-6 hours. Every effort will be made to initiate the intervention during the hospital stay for a participant in the START condition. If a participant in the START condition leaves the hospital before the intervention can be delivered, the intervention will be delivered by phone and the CM will continue to help link the participant with outpatient care in accordance with the care manager follow-up call procedures. After discharge, the CM will call each participant at least four times to provide follow-up care.

## 6.2 FIDELITY

### 6.2.1 INTERVENTIONIST TRAINING

The AMS and CM will participate in training on the START intervention as outlined in Table 5:

**Table 5: Intervention Training Modules**

| Training Module | Duration | Participants |
|---|---|---|
| Motivational Interviewing Basics | Two hours | CM, AMS |
| START Intervention Overview | Two hours | CM, AMS |
| START Intervention: Care Manager Role | Two hours | CMS |
| START Intervention: AMS Role | Two hours | AMS |
| Role Plays | Four to six hours | CM, AMS |

### 6.2.2 FIDELITY MEASURES

Fidelity to the intervention key components (collaborative care, the brief negotiated interview and addiction-focused discharged planning) as well as fidelity to the spirit of MI will be measured. Fidelity measures are described in Table 6.

**Table 6. Fidelity Measures**

| Domain | Measure | Source |
|---|---|---|
| **Collaborative Care Processes**[77] | | |
| **CM Visit** | Proportion of patients who saw CM at least one time | Registry |
| **AMS Visit** | Proportion of patients who saw AMS at least one time | Registry |
| **CM/AMS Consultation** | Proportion of patients who were discussed at least one time by CM and AMS | Registry |
| **Follow-up** | Proportion of patients who got at least 1 follow-up w/in 4 weeks after discharge | Registry |
| **Evidence-based Care: BNI**[78] | | |
| | • Proportion of patients who got pros and cons | Registry |
| | • Proportion of patients who got the readiness ruler | Registry |
| | • Proportion of patients who got an OUD-focused Action Plan | Registry |
| **Evidence-based Care: RED**[79,80] | | |

| Domain | Measure | Source |
|---|---|---|
| | • Proportion of patients for whom CM reviewed Action Plan prior to discharge | Registry |
| | • Proportion of patients who reported that CM reviewed Action Plan in a way that they understood | Follow-up survey |
| | • Proportion of patients for whom a follow-up appt was made prior to discharge | Registry |
| | • Proportion of patients who received a follow-up call within one-week of discharge | Registry |
| **Competency/Fidelity to Spirit of MI** | | |
| **Baseline** | • CM & AMS reached "good" fidelity at baseline | MITI (In-person) |
| **Midpoint (~5 months)** | • CM & AMS reached "good" fidelity at midpoint | MITI (Recorded) |

## 6.3 MEASURES TO MINIMIZE BIAS: RANDOMIZATION AND BLINDING

***Randomization***

1. Approved study staff will randomize the patient to the START or UC arm in the stratified block design using REDCap.
2. Subjects will be stratified according to prior MOUD exposure (yes/no) and randomized into START or UC.
3. A stratified, block randomization design will be used stratified by site and prior MOUD exposure, and using randomly permuted block sizes of 2, 4, and 8.
4. Research staff will access their site-specific randomization module in REDCap. Staff will enter which MOUD stratum the patient is in and the intervention arm assignment will be generated.
5. Enrollment will be continuous with the goal of reaching the desired sample size, and some sites may enroll more or less than the targeted 69 for each arm.

***Blinding***

The baseline interview will occur prior to randomization so research staff will be blind to study condition. Follow-up interviews will be performed by RAND SRG research staff who initially will be blinded to study condition, although such blinding will be broken during the course of the follow-up interview due to branching questions for START participants only.

***Minimization of Bias***

The START AMS and CM at each site will be designated to the START and will not see non-START patients for consultation during the study period once patients have been randomized.

While it is likely (and expected) that medical teams will treat patients in both the experimental and usual care (UC) conditions, patients in the START will have an enhanced experience because they (unlike

patients in the UC condition) will be receiving START components designed to increase their readiness to take the medication and link to follow-up care. Moreover, the support provided by the START to medical teams for each START patient is the component that we hypothesize will take the burden off the medical team and increase the likelihood of the patient receiving medication. Even as medical teams become more aware of MOUD and perhaps increase prescribing, we hypothesize that patients whose medical teams receive support for their START patients will still be more likely to receive medication, even if medical teams are aware they can prescribe these medications. Thus, over time, while we might see increased medication initiation for patients in both study conditions, we still hypothesize statistically and clinically significant differences for patients in the START group. Nevertheless, we still plan to safeguard against any biases and effects of UC patients receiving the START. The research team will review electronic health record data to determine whether the START AMS or CM accidentally treated UC patients.

## 6.4 STUDY INTERVENTION/EXPERIMENTAL MANIPULATION ADHERENCE

N/A

## 6.5 CONCOMITANT THERAPY

N/A

### 6.5.1 RESCUE THERAPY

N/A

# 7 STUDY INTERVENTION/EXPERIMENTAL MANIPULATION DISCONTINUATION AND PARTICIPANT DISCONTINUATION/WITHDRAWAL

## 7.1 DISCONTINUATION OF STUDY INTERVENTION/EXPERIMENTAL MANIPULATION

When a subject discontinues from the START intervention but not from the study, either because they decline to participate or leave the hospital, remaining study procedures will be completed as indicated by the study protocol.

The data to be documented at the time of study intervention discontinuation will include the following:

- The reason(s) for discontinuing the participant from the intervention, and methods for determining the need to discontinue (e.g., patient declined/refused, patient left AMA, unable to contact the patient after discharge)

- Patients who discontinue the intervention will still be contacted for their 1-month follow-up assessment.

## 7.2 PARTICIPANT DISCONTINUATION/WITHDRAWAL FROM THE STUDY

Participants are free to withdraw from participation in the study at any time upon request.

Participant discontinuation or withdrawal from the study will be recorded on the Case Report Form (CRF). Subjects who sign the informed consent form, are randomized and receive the study intervention, and subsequently withdraw or are discontinued from the study, will not be replaced.

Investigators will not withdraw a participant from the study unless the participant withdraws consent to use data not already collected, as all participants will be included in the analysis whether or not they receive the intervention.

Intent-to-Treat. All participants who are randomized are included in the statistical analysis and analyzed according to the group they were originally assigned, regardless of what treatment (if any) they received. This method allows the investigator (or consumer of the medical literature) to draw accurate (unbiased) conclusions regarding the effectiveness of an intervention. This method preserves the benefits of randomization, which cannot be assumed when using other methods of analysis.

## 7.3 LOST TO FOLLOW-UP

A participant will be considered lost to follow-up only if they do not complete the follow-up interview within 90 days of discharge.

The following actions must be taken if a participant cannot be reached for their follow-up interview:

- Before a participant is deemed lost to follow-up, the investigator or designee will make every effort to regain contact with the participant (where possible, up to 20 telephone calls, a Lexus/Nexus search, and, if necessary, a certified letter or visit to the participant's last known address or local equivalent methods). These contact attempts will be documented in the participant's study file.
- Should the participant continue to be unreachable, he or she will be considered to be lost to follow-up but will not be considered withdrawn from the study.
- Participants who are unable to complete the follow-up interview due to imprisonment will be considered to be lost to follow-up but will not be considered withdrawn from the study.
- Participants who are imprisoned and then released while still within the follow-up period will still be considered enrolled in the study and will be contacted to complete the follow-up interview.

# 8 STUDY ASSESSMENTS AND PROCEDURES

## 8.1 ENDPOINT AND OTHER NON-SAFETY ASSESSMENTS

### 8.1.1 ELIGIBILITY SCREENING

1. Approved study staff will conduct screening (Appendix 10.1), including a validated assessment (ASSIST) and demographic data.
2. Screening will be conducted in person or remotely using an approved platform (REDCap).
3. Screening data will be entered into a REDCap electronic form.
4. Screening data from patients who do not enroll in the study will be anonymous, i.e., they will not be linked to a MRN or any identifying information.

### 8.1.2 BASELINE INTERVIEW

1. Approved study staff will conduct an in-person or remote 30-40-minute baseline interview (Appendix 10.2).
2. Interview data will be recorded on a tablet or computer into a web-based survey system (REDCap).
3. Each site will be responsible for remunerating their participants per their institutional practice.

### 8.1.3 1-MONTH FOLLOW-UP INTERVIEW

1. Interviewers from the RAND Survey Research Group (SRG) will conduct a 1-month follow-up interview (Appendix 10.2) by telephone after the patient is discharged from the hospital.
2. The Statistics and Data Coordinating Center (SDCC) at UNM will provide contact information to RAND SRG through secure file transfer protocol.
3. The interview will be 30-40 minutes long.
4. RAND SRG will be responsible for remunerating participants per their institutional practices.
5. The interview will take place between 30 and 90 days after hospital discharge.

### 8.1.4 PROCEDURAL FLOW

Procedural flow and designation of research versus standard of care are shown in Table 7.

**Table 7. RCT Procedure Designation (Research v. Standard of Care)**

| **Research Procedures** | **Baseline visit** | **During course of hospital stay** | **1-month post-discharge** |
|---|---|---|---|
| Eligibility | R | | |
| Informed Consent | R | | |
| Randomization | R | | |
| Sociodemographic Data | R | | |

| | | | |
|---|---|---|---|
| Interviews (Baseline and follow-up) – Pain (PEG), Depression (PHQ-9), Anxiety (GAD-7), SUD treatment/substance use (NSDUH), Overdose, Healthcare utilization (GAIN), Substance Use Severity (PROMIS), Employment | R | | R |
| Interviews (Baseline only): Experience of stigma, Social Support (MSPSS), Opinions about MAT (OAMAT), Significant other OUD, criminal justice involvement | R | | |
| Interview (Follow-up only): MAT utilization, patient experience of chronic illness care (PACIC), therapeutic alliance (CAHPS)[a], Satisfaction with START intervention [a] | | | R |
| Medication for opioid use disorder [b] | | S | |
| Therapy for opioid use disorder [b] | | S | |
| START Intervention | | | |
| START Addiction Medicine Specialist (AMS) coordinates/delivers team-based care[a,c] | | R | R |
| START Care Manager (CM) Coordinates/delivers team-based care[a,c] | | R | R |

**LEGEND**
**R** = Research item/procedure done only for research purposes and covered by the study
**S** = Standard of care item/procedure that is part of regular care and billed to the patient/insurance

**Footnotes:**

a. Only for patients randomized to the START intervention arm of the study.
b. Available to but not required for both groups: Usual Care and START intervention.
c. The START intervention utilizes established standard-of-care services and procedures (care manager, addiction medicine specialist, medication treatment, therapy, etc.) and helps integrate them into the patient's care in a systematic way. It is this planned coordination and integration that are the intervention, not the services themselves. The START intervention includes Brief Negotiated Interview and addiction focused discharge planning and follow-up.

### 8.1.5 VARIABLES COLLECTED

Outcome variables, measures, and sources are shown in Table 8. Covariates and potential mediators/moderators are shown in Table 9.

**Table 8. Outcome Variables**

| Outcome | Endpoint | Data Source |
|---|---|---|
| **Primary Outcomes** | | |
| In-hospital initiation of MOUD therapy | Proportion of patients in each arm who initiate MOUD prior to discharge, defined as use of any FDA-approved pharmacotherapy for OUD, including buprenorphine, naltrexone and methadone | EMR |
| Linkage to follow-up OUD care | Proportion of patients in each arm who attend at least one OUD-related care visit within 30 days of hospital discharge | 1-month interview; optional validation through follow-up with service provider |
| **Secondary Outcomes** | | |
| OUD-specific discharge plan | Proportion of patients in each arm with an after-hospital care plan that specifies a date and time for a post-discharge addiction care appointment | EMR chart review |
| Any post-discharge MOUD utilization | Proportion of patients in each arm who initiate MOUD or continue MOUD treatment within 30 days following hospital discharge | 1-month interview; optional validation through follow-up with service provider |
| Post-discharge outpatient medical care | Proportion of patients in each arm who complete at least one visit to an outpatient medical provider within 30 days of hospital discharge | 1-month interview |
| Past 30-day number of days with any opioid use | Mean (or median, depending on distribution) days of use in the past 30 days after hospital discharge–Adapted National Survey of Drug Use and Health (NSDUH)[81] | Baseline interview<br>1-month interview |

**Table 9. Measures (Outcomes, Covariates, Potential Mediators and Moderators)**

| Variable | Measure | Data Source | Response Values/Scales |
|---|---|---|---|
| **Sociodemographics** | ***(Covariates; Potential Moderator)*** | | |
| • Age | | Eligibility Screener | Continuous |
| • Sex (Assigned at Birth) | | Eligibility Screener | Binary |
| • Gender Identity | | Eligibility Screener | Categorical (1-4) |
| • Hispanic | | Eligibility Screener | Binary |
| • Race | | Eligibility Screener | Categorical (1-5) |
| • Current homeless status | | Eligibility Screener | Binary |
| • Marital status | | Baseline Interview | Categorical (1-6) |

| Variable | Measure | Data Source | Response Values/Scales |
|---|---|---|---|
| • Income | | Baseline Interview | Continuous |
| • Education | | Baseline Interview | Categorical (1-20) |
| • Insurance type | Payer name | EMR | Text (code to numeric) |
| **Mental Health Symptoms** | ***(Covariates; Potential Moderator/ Mediator)*** | | |
| • Depression (9 items) | PHQ-9 [82,83] | Baseline Interview<br>1-month Follow-up | Likert-type (1-4) |
| • Anxiety (7 items) | GAD-7[84-86] | Baseline Interview<br>1-month Follow-up | Likert-type (1-4) |
| **Social Support Scale** | ***(Covariate; Potential Moderator)*** | | |
| • Social support: Family, Friends, Significant Other (6 items; 2 each scale) | Modified Multidimensional Scale of Perceived Social Support[87] | Baseline Interview<br>1-month Follow-up | Likert-type (1-7) |
| **Medical Symptoms/Treatment** | ***(Covariates; potential mediator/moderator)*** | | |
| • Overdoses (lifetime, past 3 mos) | N/A | Baseline Interview<br>1-month Follow-up | Continuous |
| • Primary and secondary diagnosis (inpatient stay) | Medical or mental health conditions as determined by the inpatient physician | EMR | Numeric text (ICD codes) |
| • Pain intensity and duration | PEG[88] | Baseline Interview<br>1-month Follow-up | 0-10 scale |
| • Length of hospital stay | Days in hospital | EMR | Continuous |
| **Substance Use Treatment History** | ***(Covariates; potential moderator)*** | | |
| • Ever used an MOUD<br>• Times started an MOUD | N/A | Eligibility Screener | • Binary<br>• Continuous |
| • Type of MOUD medication | N/A | Eligibility Screener | Categorical (1-4) |
| • Treatment other than MOUD<br>• Times had treatment other than MOUD | N/A | Eligibility Screener | • Binary<br>• Continuous |
| **Recent Substance Use Treatment Utilization; Opinions; Consequences;** | ***(Outcomes*; Covariates)*** | | |

| Variable | Measure | Data Source | Response Values/Scales |
|---|---|---|---|
| SUD Treatment Utilization* (5 items)<br>• Past 90 days baseline<br>• Past 30 days from discharge follow-up<br>***Linkage outcome*** | Adapted from National Survey on Drug Use and Health (NSDUH)[81] | Baseline Interview<br>1-month Follow-up (optional validation through follow-up with service provider) | Binary |
| Healthcare Utilization (ER, Inpatient, Outpatient) Related to SUD (5 items)<br>• Past 90 days baseline<br>• Past 30 days follow-up | Adapted from Global Appraisal of Individual Needs (GAIN)[89] | Baseline Interview<br>1-month Follow-up | Continuous |
| • Familiar with MOUD<br>• Opinions about MOUD (3 items) | Opinions about MAT (OAMAT)[90] | Baseline Interview | Likert-type (1-5) |
| Severity of Substance Use (7-items) | PROMIS | Baseline Interview<br>1-month follow-up | Likert-type (1-5) |
| Patient Experience of Stigma (5 items) | Adapted from Grosso et al. 2019.[91] | Baseline Interview | Binary |
| Patient Experience of Chronic Illness Care (11 items) | Patient Assessment of Chronic Illness Care (PACIC)[92] | 1-month Follow-up | Binary |
| Criminal Justice Involvement<br>• Ever arrested<br>• Times arrested past 90 | Locally developed | Baseline Interview | • Binary<br>• Continuous |
| **Intervention – Related** | ***(Covariates; Exploratory feasibility outcomes)*** | | |
| Intervention “dose”; exposure | Amount time spent with patient<br>Number of encounters with patient | START Registry (Deidentified) | Continuous |
| | | | |
| Therapeutic Alliance | Consumer Assessment of Healthcare Providers and Systems (CAHPS®)[93] | 1-month follow-up (START only) | Binary |
| Satisfaction with START intervention<br>• Helpful in addressing OUD (1 item)<br>• Feedback (5 items) | Locally developed | 1-month Follow-up (START only) | • Binary<br>• Open text |

**Minimum EMR variables needed for recruitment, as available at each site, are as follows:**

- Demographics: Patient name; MRN; CSN; Sex; DOB
- Hospital encounter data: Hospital admission date and time; Inpatient admission date and location; Reason for admission; Admission diagnosis; Admitting physician; Attending provider
- Interpreter needed – exclusion criterion unless Spanish
- Attending/PCP
- DRGs (Diagnosis Related Group): F11 (Opioid Use Disorders); F19 (Other psychoactive substance use); Overdose
- Past medication orders: Buprenorphine; Naloxone; Naltrexone
- Social history (if available): Drug usage

**Minimum EMR elements needed to compile outcome variables are:**

- Hospital encounter data: Type; Dates; Disposition; Attending provider; PCP; Psychiatry consult;
- Reason for admission
- Diagnoses
- Inpatient medication (listed by generic names): Buprenorphine; Buprenorphine/Naloxone; Methadone; Naltrexone; Naloxone.
- Hospital utilization metrics: Length of stay; Inpatient admissions in prior 12 months; ED admissions in prior 12 months; Number of 30-day readmissions
- Insurance type

## 8.2 SAFETY ASSESSMENTS

Because MOUD initiation will take place in the hospital and is not considered the intervention, safety will be monitored by the medical team as part of usual medical care. Safety concerns that arise during baseline and follow-up interviews will be reported to the PI.

## 8.3 ADVERSE EVENTS AND SERIOUS ADVERSE EVENTS

### 8.3.1 DEFINITION OF ADVERSE EVENTS

This protocol defines an adverse event (AE) as any unfavorable and unintended symptom or disease that an investigator or study staff learns about which occurs during a patient's enrollment in the study, if it is considered by the site study team to be possibly related to a study treatment or procedure ("possibly related" means there is a reasonable possibility that AE may have been caused by research procedures).

All AEs possibly related to a study treatment or procedure learned about during the course of a patient's enrollment in the study (from time of consent through the follow-up interview or close of the follow-up window if they do not have their follow-up interview) will be recorded and reviewed by the site PI and/or clinician and CSMC PI, and study staff, and reported further per guidelines described in section 8.3.5 and shown in Figure 4 and in Tables 10 and 11.

### 8.3.2 DEFINITION OF SERIOUS ADVERSE EVENTS

This protocol defines a serious adverse event (SAE) as an AE that an investigator or study staff learns about that is fatal, life-threatening, prolongs initial hospitalization, requires inpatient rehospitalization, or is medically significant and which the investigators and/or clinicians regard as serious based on appropriate medical judgment. With the exception of fatalities, other SAEs documented this study are considered those is possibly related to the study; other events occurring during the normal of the hospital stay will be numerous and thus not documented unless possibly related to the study.

Classification of SEVERITY AND RELATEDNESS

#### 8.3.2.1 SEVERITY OF EVENT

The following guidelines will be used to describe severity.

- **Mild** – Events require minimal or no treatment and do not interfere with the participant's daily activities or prescribed course of hospital treatment.
- **Moderate** – Events result in a low level of inconvenience or concern with the therapeutic measures. Moderate events may cause some interference with functioning or prescribed hospital treatment.
- **Severe** – Events interrupt a participant's usual daily activity or prescribed hospital treatment and may require systemic drug therapy or other treatment beyond what was prescribed for their inpatient treatment. Severe events are usually potentially life-threatening or incapacitating. Of note, the term "severe" does not necessarily equate to a "serious adverse event".

Severity will be used as a factor in determining expectedness of an event, and therefore, in determining if it requires reporting. Changes in severity will guide the duration an event is followed as well as potential changes in reporting requirements for that event.

#### 8.3.2.2 RELATIONSHIP TO STUDY INTERVENTION/EXPERIMENTAL MANIPULATION

AEs considered by study staff to be possibly related to the study and all SAEs possibly related to the study will have their relationship to study procedures, including the intervention, assessed by an appropriately trained clinician based on temporal relationship and his/her clinical judgment. The degree of certainty about causality will be graded using the categories below.

- **Definitely Related** – There is clear evidence to suggest a causal relationship, and other possible contributing factors can be ruled out. The clinical event occurs in a plausible time relationship to study procedures administration and cannot be explained by concurrent disease or other drugs or chemicals, or other events. The response to withdrawal of the study procedures should be clinically plausible. The event must be pharmacologically or phenomenologically definitive.
- **Probably Related** – There is evidence to suggest a causal relationship, and the influence of other factors is unlikely. The clinical event occurs within a reasonable time after administration of the study procedures, is unlikely to be attributed to concurrent disease or other drugs or chemicals or events, and follows a clinically reasonable response on withdrawal.
- **Unlikely to be related** – A clinical event whose temporal relationship to study procedures administration makes a causal relationship improbable (e.g., the event did not occur within a

reasonable time after administration of the study procedures) and in which other drugs or chemicals or underlying disease or events provides plausible explanations (e.g., the participant's clinical condition, other concomitant treatments).
- **Not Related** – The AE is completely independent of study procedures administration, and/or evidence exists that the event is definitely related to another etiology. There must be an alternative, definitive etiology documented by the clinician.

***Because the intervention is randomization to a collaborative care team and MOUD initiation is not considered part of the intervention, known adverse reactions to a specific medication will not be considered related to the study intervention. And, because of the likelihood of adverse events during the inpatient stay due to patients' illness or injury, most AEs/SAEs likely will not be related to the study.***

#### 8.3.2.3 EXPECTEDNESS

Study clinicians with appropriate expertise in addiction medicine will be responsible for determining whether an adverse event (AE) is expected or unexpected. An AE will be considered unexpected if the nature, severity, or frequency of the event is not consistent with the risk information previously described for the study procedures.

Expected events will not be reported or followed as AEs. Because the study is being conducted among a hospitalized population of people with OUD, patients likely will have many comorbid conditions as well as a higher likelihood of unexpected complications, death or readmission from disease. Any medical or psychiatric condition that is present at the time that the participant is screened will be considered as baseline and not reported or followed as an AE. However, if the study participant's condition deteriorates at any time during the study and is considered by site study staff to be possibly related to the study, it will be recorded as an AE. Common side-effects associated with taking MOUD will not be reported as AEs (see Appendix 11.3) and will be monitored by the medical team as part of usual medical care.

The only expected AEs from the START intervention (and not MOUD) may be psychological distress related to discussing OUD and seeking treatment.

### 8.3.3 TIME PERIOD AND FREQUENCY FOR EVENT ASSESSMENT AND FOLLOW-UP

The occurrence of an adverse event (AE) or serious adverse event (SAE) may come to the attention of study personnel during study encounters either in-person or over the phone – at enrollment, interviews, or follow-up calls), or through notification by a clinician or other source.

AEs and SAEs possibly related to the study will be captured on the case report form (CRF) in REDCap (Reporting Form) by selecting the appropriate event type, participant's clinical status (including any underlying conditions), study ID, gender and age; REDCap will then prompt for the information

applicable to the type of event be completed. Information to be collected, as applicable to the event type, may include:

- protocol title and number, PI's name, and the IRB project number
- date of enrollment and randomization
- a detailed description of the event, incident, experience, or outcome
- an explanation of the basis for determining that the event, incident, experience, or outcome represents an AE/SAE, PD, or UP
- times of awareness and onset
- concomitant medications
- dates and findings of any relevant tests/assessments that have taken place
- clinician's assessment of expectedness, severity, and relationship to study procedures (assessed only by those with the training and authority to make a diagnosis)
- time of resolution/stabilization of the event.
- a description of any changes to the protocol or other corrective actions that have been taken or are proposed in response to the event

All AEs and SAEs learned about by study staff during the patient's enrollment in the study that are possibly related to the study will be documented regardless of expectedness. All AEs and SAEs will be followed to adequate resolution.

Changes in the severity of an AE will be documented to allow an assessment of the duration of the event at each level of severity to be performed. Documentation of onset and duration of each episode will be maintained for AEs characterized as intermittent.

The study coordinator or designated staff member at each site will record events with start dates occurring any time after informed consent is obtained and end dates occurring up until 7 (for non-serious AEs) or 30 days (for SAEs) after the last day of study participation (i.e., end of follow-up window). Events will be followed for outcome information until resolution or stabilization.

### 8.3.4 ADVERSE EVENT REPORTING

When deciding whether an event should be recorded on the reporting form in REDCap, each site's study coordinator or designated staff member will consult with the site PI and/or their designated clinical study staff member. Following consultation, AEs and SAEs possibly related to the study will be recorded by each site's study coordinator or designated staff member in the REDCap Reporting Form. Each site PI and/or their designated clinical study staff member will review the form and investigate each event further to ascertain: (1) nature of the event (disclosure, psychological distress, or physical harm), (2) type and degree of actual harm (potential, social/psychological, physical), (3) type and degree of potential harm (social/psychological, physical), (4) potential attribution to the research study, (5) potential contributing factors, and (6) potential for recurrence. The research team (the site PI or designee, with the study PI and staff) will confer on how to handle the events, including discussing them

with the IRB, if uncertainty exists. The only exception to this rule is patient death, which always will be recorded on the REDCap form regardless of whether it is considered possibly related to the study.

AEs possibly related to the study will be provided to Dr. Danovitch and the CSMC study coordinator in a weekly summary report. SAEs will be sent to Dr. Danovitch and the CSMC Study Coordinator upon entry into REDCap, but regular AEs will not.

**IRB reporting:** AEs that are not SAEs do not need to be reported to CS-IRB. See below for SAE reporting.

**DSMB reporting:** All AEs considered possibly related to the study and all SAEs will be summarized and reported to the DSMB at three time points during data collection.

### 8.3.5 SERIOUS ADVERSE EVENT REPORTING

Upon consultation with the site PI or and/or their designated clinical study staff member, SAEs possibly related to the study will be recorded by each site's study coordinator or designated study staff in the REDCap Reporting Form. All patient deaths learned about by study staff will be recorded and reported regardless of whether they are considered possibly related to the study. Each site PI and/or designated clinical study staff member will review the form and investigate each event further to ascertain: (1) nature of the event (psychological distress, or physical harm), (2) type and degree of actual harm (potential, social/psychological, physical), (3) type and degree of potential harm (social/psychological, physical), (4) potential attribution to the research study, (5) potential contributing factors, and (6) potential for recurrence. The research team (the site PI or designee, with the study PI and staff) will confer on how to handle the events, including discussing them with the IRB, if uncertainty exists.

SAEs will be sent to Dr. Danovitch and the CSMC Study Coordinator upon entry into REDCap. Dr. Danovitch and the CSMC study coordinator who will review the form and notify the CS-IRB and the DSMB as required and outlined below.

**IRB reporting:** SAEs must be reported to the Cedars-Sinai IRB if they are unexpected (e.g., not listed in the consent form) and at least probably related to the research (with the exception of patient death, which is always reported). Reportable events must be reported to the CSMC study team as soon as possible but no later than 10 business days from the relying site's awareness of the event. The CSMC study team must submit the event in CS-IRB as soon as possible but no later than 10 business days from the CSMC study team's awareness of the event, via a Reportable New Information (RNI) form in CS-IRB, selecting the most appropriate type of report, and including copies of any relevant documentation.

**DSMB reporting:** All SAEs possibly related to the study and all patient deaths will be summarized and reported to the DSMB at three time points: initial, interim, final. Ad hoc review may take place if there is a study-related SAE.
Patient deaths that the study team becomes aware of during the follow-up period, regardless of the relationship to the study, must be reported to the CSMC study team as soon as possible but within 10

business days of the site's awareness of the event. These events will be reported to the DSMB by email within 10 business days of CSMC's awareness of the event.

**NIH reporting:** SAEs possibly related to the study will be reported to the Project Officer by email within 10 business days of CSMC's awareness of the event.

#### 8.3.6 REPORTING EVENTS TO PARTICIPANTS

N/A

#### 8.3.7 EVENTS OF SPECIAL INTEREST

N/A

#### 8.3.8 REPORTING OF PREGNANCY

N/A

**Figure 4. AE/SAE Reporting Flowchart**

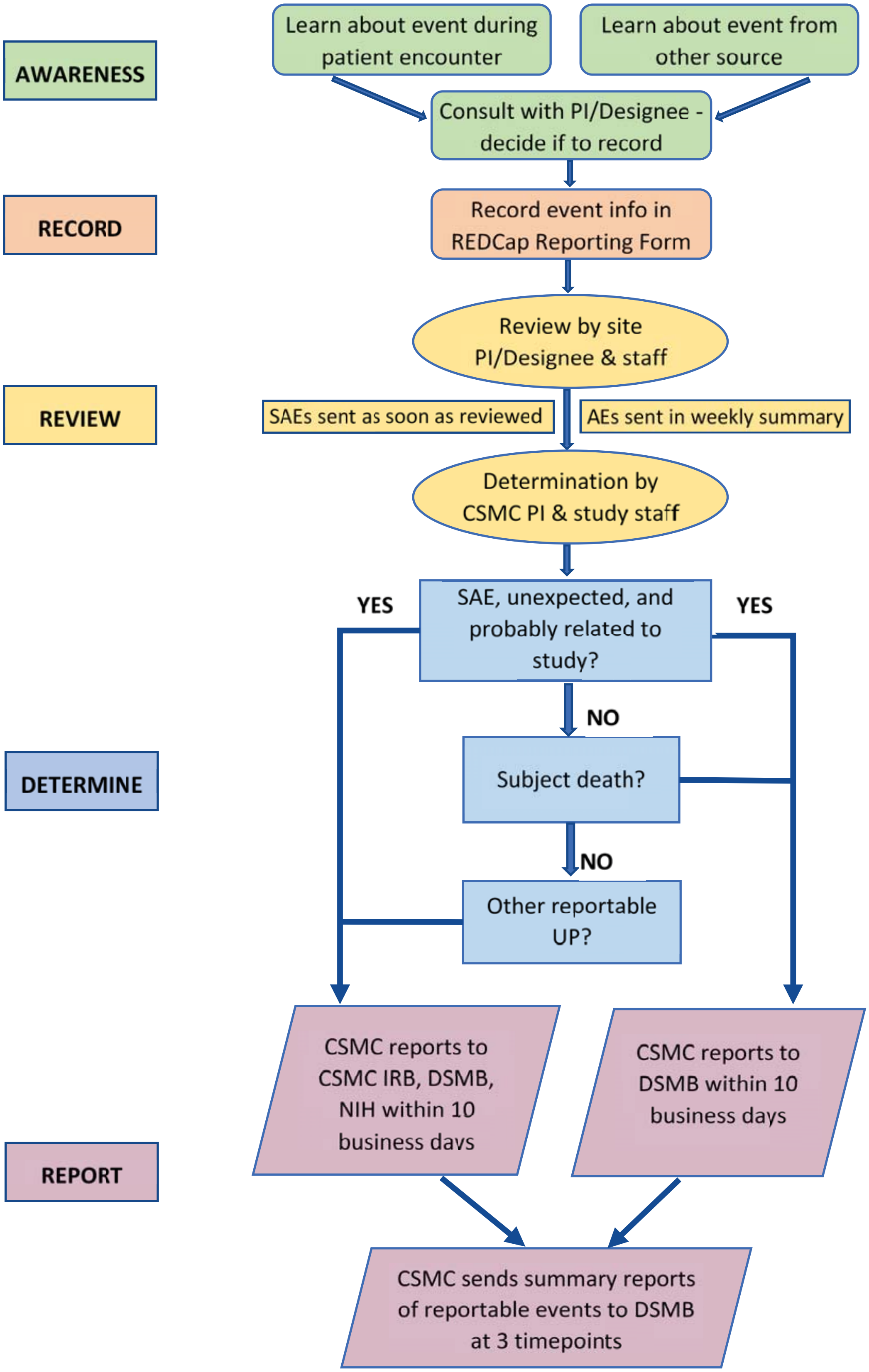

**Table 10. Site AE/SAE Reporting**

| Nature of Event | Examples | Study-Related | Reported By | Reported To | Format | Reporting Timeframe (From Awareness) |
|---|---|---|---|---|---|---|
| **ADVERSE EVENTS (Events deemed by site study staff to be at least possibly related to the study)** | | | | | | |
| Expected | Mild side effects of MOUD or the psychosocial intervention | At least possibly | N/A (Expected events are not reportable) | | | |
| Unexpected | Unknown | At least possibly | Site study staff/SDCC | CSMC PI/study staff | REDCap (AE) Reporting Form | Weekly summary prepared by SDCC |
| **SERIOUS ADVERSE EVENTS (Events deemed by site study staff to be at least possibly related to the study, except patient death)** | | | | | | |
| Expected | Unknown | At least possibly | N/A (Study does not have any expected SAE) | | | |
| Unexpected | Deteriorating condition related to expected side effects of MOUD or the psychosocial intervention; other unexpected side effects; readmission | At least possibly | Site study staff | Site PI, CSMC PI/study staff | REDCap (SAE) Reporting Form | As soon as recorded, within 10 business days |
| Unexpected | Patient death | Unrelated or related | Site study staff | Site PI, CSMC PI/study staff | REDCap (SAE) Reporting Form | As soon as recorded, within 10 business days |
| **UNANTICIPATED PROBLEMS** | | | | | | |
| Breach of confidentiality or privacy | PHI shared with another site; wrong patient email used | Related | Site study staff | Site PI, CSMC PI/study staff | REDCap (UP) Reporting Form | As soon as recorded, within 10 business days |
| Patient complaint (unresolved) | Claim of non-payment | Related | | | | |
| Incarceration | Patient incarcerated at any point during follow-up period (even if released) | N/A | | | | |
| New risk | Recall of MOUD given to patient | Related | | | | |

| Nature of Event | Examples | Study-Related | Reported By | Reported To | Format | Reporting Timeframe (From Awareness) |
|---|---|---|---|---|---|---|
| **NON-COMPLIANCE** | | | | | | |
| Protocol Deviations | Eligibility or consent deviation; HIPAA violation | Related | Site study staff | Site PI, CSMC PI/study staff | REDCap (PD) Reporting Form | As soon as recorded, within 10 business days |

**Table 11. CSMC AE/SAE Reporting**

| Nature of Event | Examples | Study-Related | Reported By | Reported To | Format | Reporting Timeframe (From Awareness) |
|---|---|---|---|---|---|---|
| **ADVERSE EVENTS (Events deemed by CSMC PI/study staff to be <u>at least possibly related</u> to the study)** | | | | | | |
| Expected | Mild side effects of MOUD or the psychosocial intervention | At least possibly | N/A (Expected events are not reportable and the study does not have expected SAE) | | | |
| Unexpected | Unknown | At least possibly | CSMC study staff | DSMB | AE Summary | At three time points during data collection: initial, interim, final. Ad hoc review may take place if there is a study-related SAE. |
| **SERIOUS ADVERSE EVENTS (Events deemed by CSMC PI/study staff to be <u>at least possibly related</u> to the study, except patient death)** | | | | | | |
| Expected | Unknown | At least possibly | N/A (Expected SAEs are not reportable to IRB) | | | |
| Unexpected | Deteriorating condition related to expected side effects of MOUD or the psychosocial intervention; other unexpected side effects; readmission | Probably or Definitely | CSMC study staff | CSMC IRB / DSMB, NIH | RNI Form (IRB) / SAE Form (DSMB, NIH) | Within 10 business days |
| Unexpected | Patient death | Unrelated or related | CSMC study staff | CSMC IRB (if probably related) / | RNI Form / SAE Form | Within 10 business days |

| Nature of Event | Examples | Study-Related | Reported By | Reported To | Format | Reporting Timeframe (From Awareness) |
|---|---|---|---|---|---|---|
| | | | | DSMB (all deaths) | | |
| **UNANTICIPATED PROBLEMS** | | | | | | |
| Breach of confidentiality or privacy | PHI shared with another site; wrong patient email used | Related | CSMC Study Staff | DSMB / CSMC IRB | RNI Form (IRB) / Summary (DSMB) | Within 10 business days (IRB); At three time points during data collection. (DSMB) |
| Patient complaint (Unresolved) | Claim of non-payment | Related | | | | |
| Incarceration | Patient incarcerated at any point during follow-up period (even if released) | N/A | | | | |
| New risk | Recall of MOUD given to patient | Related | | | | |
| **NON-COMPLIANCE** | | | | | | |
| Protocol Deviations | Eligibility or consent deviation; HIPAA violation | Related | CSMC Study Staff | CSMC IRB / DSMB, NIH | RNI Form (IRB) / PD Form (DSMB, NIH) | Within 10 business days |

## 8.4 UNANTICIPATED PROBLEMS

### 8.4.1 DEFINITION OF UNANTICIPATED PROBLEMS

This protocol uses the definition of Unanticipated Problems (UPs) as defined by the Office for Human Research Protections (OHRP). OHRP considers unanticipated problems involving risks to participants or others to include, in general, any incident, experience, or outcome that meets **all** of the following criteria:

- Unexpected in terms of nature, severity, or frequency given (a) the research procedures that are described in the protocol-related documents, such as the Institutional Review Board (IRB)-approved research protocol and informed consent document; and (b) the characteristics of the participant population being studied;
- Related or possibly related to participation in the research ("possibly related" means there is a reasonable possibility that the incident, experience, or outcome may have been caused by research procedures); and
- Suggests that the research places participants or others at a greater risk of harm (including physical, psychological, economic, or social harm) than was previously known or recognized.

UPs may or may not be AEs/SAEs.

### 8.4.2 UNANTICIPATED PROBLEMS REPORTING

Site investigators will report UPs to Dr. Danovitch. Dr. Danovitch will report UPs to the CSMC Institutional UPs that do not otherwise meet the definition of an AE/SAE (e.g., breach of confidentiality or privacy, unresolved patient complaints, incarceration, new risks) will be recorded by each site's study coordinator in the REDCap Reporting Form. Each site PI and/or clinical designee will review the form and investigate each event further to ascertain: (1) nature of the event (disclosure, psychological distress, or physical harm), (2) type and degree of actual harm (potential, social/psychological, physical), (3) type and degree of potential harm (social/psychological, physical), (4) potential attribution to the research study, (5) potential contributing factors, and (6) potential for recurrence. The research team (the site PI or designee, with the study PI and staff) will confer on how to handle the events, including discussing them with the CSMC IRB, if uncertainty exists. If disclosures of sensitive individually identifiable information may have occurred outside the research team, the study will adhere to HIPAA and other relevant considerations.

Completed UP Forms will be automatically sent through REDCap to Dr. Danovitch and the CSMC study coordinator, who will review and notify the Cedars-Sinai IRB, the DSMB, and the study Project Officer as required and outlined below.

- UPs that are adverse events (AEs) or serious adverse events (SAEs) will be reported as described in Section 8.3.
- Any other UPs will be reported per the same guidelines as AEs/SAEs.

#### 8.4.3 REPORTING UNANTICIPATED PROBLEMS TO PARTICIPANTS

N/A

# 9 STATISTICAL CONSIDERATIONS

## 9.1 STATISTICAL HYPOTHESES

***Primary Hypotheses:***

Primary Hypothesis 1: We hypothesize that, compared to patients who receive usual care, patients in the START condition will be more likely to initiate MOUD while hospitalized. Alternatively, our null hypothesis is that there will be no difference in likelihood of in-hospital MOUD initiation between patients in the START and UC conditions.

Primary Endpoint 1: Proportion of patients in each arm who initiate MOUD prior to discharge, defined as use of any FDA-approved pharmacotherapy for OUD, including buprenorphine, naltrexone and methadone.

Primary Hypothesis 2: We hypothesize that, compared to patients who receive usual care, patients in the START condition will be more likely to receive linkage to post-discharge MOUD care (i.e., attend at least one OUD-related care visit within 30 days of hospital discharge). Alternatively, our null hypothesis is that there will be no difference in linkage to post-discharge MOUD care for patients in the START condition compared to those in UC condition.

Primary Endpoint 2: Proportion of patients in each arm who attend at least one OUD-related care visit within 30 days of hospital discharge.

***Secondary Hypotheses:***

Secondary Hypothesis 1: We hypothesize that, compared to patients who receive usual care, patients in the START condition will be more likely to receive addiction-focused discharge planning. Alternatively, our null hypothesis is that there will be no difference in likelihood of receiving addiction-focused discharge planning between patients in the START and UC conditions.

Secondary Endpoint 1: Proportion of patients in each arm with an after-hospital care plan that specifies a date and time for a post-discharge addiction care appointment.

Secondary Hypothesis 2: We hypothesize that, compared to patients who receive usual care, patients in the START condition will be more likely to receive MOUD treatment after discharge (i.e., initiate MOUD or continue MOUD treatment within 30 days following hospital discharge). Alternatively, our null

hypothesis is that there will be no difference in likelihood of receiving MOUD treatment between patients in the START and UC conditions.

Secondary Endpoint 2: Proportion of patients in each arm who initiate MOUD or continue MOUD treatment within 30 days following hospital discharge.

Secondary Hypothesis 3: We hypothesize that, compared to patients who receive usual care, patients in the START condition will be more likely to receive linkage to post-discharge medical care (i.e., complete at least one visit to an outpatient medical provider within 30 days of hospital discharge). Alternatively, our null hypothesis is that there will be no difference in linkage to post-discharge medical care for patients in the START condition compared to those in UC condition.

Secondary Endpoint 3: Proportion of patients in each arm who complete at least one visit to an outpatient medical provider within 30 days of hospital discharge.

Secondary Hypothesis 4: We hypothesize that, compared to patients who receive usual care, patients in the START condition will be more likely to significantly reduce opioid use after discharge (i.e., days of opioid use in the 30 days between discharge and follow-up). Alternatively, our null hypothesis is that there will be no difference in likelihood of reducing opioid use between patients in the START and UC conditions.

Secondary Endpoint 4: Mean (or median, depending on distribution) days of use in the past 30 days after hospital discharge – Adapted National Survey of Drug Use and Health (NSDUH).

## 9.2 SAMPLE SIZE DETERMINATION

In-patient MOUD initiation: A sample size of n=414 (allowing for 20% attrition) and adjusted type I error rate of 2.5% provides 84% power to detect an OR=2.3 comparing the in-patient MOUD initiation rates in the CCT and UC arms, stratified on prior MOUD use. Based on literature, 14% of UC patients who are MOUD-naïve initiate MOUD in hospital.[5] Assuming the average of MOUD-naïve and MOUD-experienced in-patient MOUD initiation rates is 20%, we have an adequate sample size and power to detect this increase of in-patient MOUD initiation in the CCT arm (37%) compared to UC.[5,59,61]

Linkage to OUD Care: We base the sample size estimate on the linkage to care measure since the probabilities of successful linkage are lower than for in-patient MOUD initiation. Linkage to care rates reported in the literature range between 10%-17% in usual care settings. To err on the side of caution, we estimate linkage to care in UC for MOUD-naïve and MOUD-experienced to be 5% and 10%,[5,59,61,94] respectively, yielding an average of 7.5%. We hypothesize that at least 20% of patients randomized to the START arm will link to OUD care (attend at least one OUD-related visit) within 30 days following discharge. Assuming a Bonferroni-corrected, two-sided type I error rate of 2.5% to adjust for two primary endpoints, we will enroll a minimum of 414 patients (207 in each intervention arm) to have 80%

power to detect this difference. This estimate includes an adjustment for up to 20% attrition. This effect size corresponds to a clinically meaningful odds ratio of 3.0. Prior studies in different settings have found larger effects,[61,94,95] supporting our ability to conduct this test.

Sample size calculations for the primary endpoints were performed in PASS 14 using stratified Mantel-Haenszel tests for two proportions between two groups,[96] with strata defined as 50% MOUD-naïve and 50% MOUD-experienced.[61,94,95,97,98] Due to the short 1-month duration of participation, subject withdrawal from the study is not anticipated to be significant.

## 9.3 POPULATIONS FOR ANALYSES

Intention-to-Treat (ITT) Analysis Population (i.e., all randomized participants) will be included in analyses. Participants who complete the informed consent or part of the baseline interview but are not randomized will be excluded from analysis.

## 9.4 STATISTICAL ANALYSES

### 9.4.1 GENERAL APPROACH

Baseline characteristics will be summarized with descriptive statistics such as means and standard deviations for continuous variables and frequencies and percentages for categorical variables. Summaries will be presented overall, by intervention arm, and by previous MOUD use. Continuous baseline demographics and characteristics will be compared with *t* tests or Wilcoxon rank sum tests, as appropriate. Categorical variables will be compared with chi-square or Fisher exact tests, as appropriate. Corresponding confidence intervals will be reported in addition to p-values. The primary and secondary analyses will be performed for the intention-to-treat population, which consists of all randomized subjects who meet inclusion and exclusion criteria.

Missing Data: Study endpoints are cross-sectional in time. Every effort will be made to obtain all necessary outcome and covariate data. We will use inverse probability weighting and multiple imputation (IPW-MI) to adjust for missing covariate data.[99] Specifically, we will examine whether observable baseline characteristics differ by attrition status, and if so we will adjust our comparisons using weights. MI will be used to impute intermittently missing data for study completers. We will not impute outcomes, but only covariates.

### 9.4.2 ANALYSIS OF PRIMARY AND SECONDARY ENDPOINTS

Primary Endpoint Analyses:

Unadjusted point estimates and confidence intervals for proportions and means will be reported by arm and by prior MOUD use for endpoints. Primary endpoints will be compared between arms by fitting a multivariable logistic regression model to each that includes as independent variables: intervention arm,

prior MOUD exposure and site, as well as relevant baseline characteristics as covariates, including age, insurance status (as a marker for income), race, and ethnicity. Additional covariates that may be included are substance use severity, homelessness and length of index hospitalization, as well as any other variables also thought to be associated with outcomes that demonstrated imbalance between treatment arms.[95] Site will be included as a fixed effect to reflect the study design and to control for potential variability in CCT implementation. Odds ratios and their Bonferroni-adjusted 97.5% Wald confidence intervals will be reported for the two primary outcomes.

Secondary Endpoint Analyses:

Similar analyses as described for the primary endpoints will be performed for these secondary proportions outcomes, but instead reporting 95% confidence intervals. For Secondary Endpoint #4, a general linearized model to number of days of opioid use will be fitted along with the covariates described for the logistic regression models. An appropriate link function will be identified and used based on the distribution of the outcome data.

### 9.4.3 SAFETY ANALYSES

N/A

### 9.4.4 BASELINE DESCRIPTIVE STATISTICS

Intervention groups will be compared on baseline characteristics (e.g., demographics, mental health characteristics) using descriptive statistics. Planned comparisons are below. Additional comparisons may be added to the statistical analysis plan.

- Site
- Age (mean, SD)
- Assigned Sex
- Gender Identity
- Ethnicity (Hispanic/Latino)
- Race
    - Black/African American
    - White
    - Asian/Pacific Islander
    - American Indian/Alaska Native
    - Multiple races
    - Other race
- Insurance status
- Homeless
- Depression (PHQ) and anxiety (GAD)

### 9.4.5 PLANNED INTERIM ANALYSES

We will conduct an interim analysis on the second primary outcome (linkage to post-discharge care) when we have enrolled 288 participants. 288 is the revised analytic sample size needed for the first primary outcome - medication initiation. This revised analytic sample size is based on the actual balance between those with and without prior MOUD use (our strata) as well as the actual attrition rate rather than our hypothetical pre-study calculations, as well as our desire to minimize participant burden.

Including 288 patients in the interim analysis allows for approximately n = 288 × 0.70 (actual attrition rate) = 202, or ~68%, of our final analytic sample size for the linkage outcome (202/299). We will utilize an alpha-spending method[100,101] to ensure that, should we continue the study until full enrollment, we control the family-wise type I error rate to be 2.5% for each outcome. Using a two-sided test, this approach will allow us to assess superiority of the intervention over the control or the control over the intervention. If we reject the null hypothesis at the interim analysis, we will discontinue the study. Should we fail to reject the null hypothesis at the interim analysis, we will continue to full enrollment before conducting the final analysis.

### 9.4.6 SUB-GROUP ANALYSES

Past research suggests that sex or gender does not moderate START effectiveness.[102] However, we will conduct exploratory analyses to see if patient sex or gender, or race/ethnicity has an effect on primary outcomes or retention. Adjusted odds ratios and their 95% confidence intervals will be calculated from interaction effects between treatment group and sex or gender from the specified linear models for the primary and secondary outcome measures.

### 9.4.7 TABULATION OF INDIVIDUAL PARTICIPANT DATA

Individual participant data will be aggregated and summarized descriptively. Inference will be made on the entire study sample and not at the individual participant level.

### 9.4.8 EXPLORATORY ANALYSES

To explore possible mechanisms of how START works, we will conduct the following exploratory analyses: (1) Assess the mediating effect of inpatient MOUD initiation on use of MOUD and linkage with OUD treatment post-discharge; (2) Assess the mediating effect of completion of an OUD-specific discharge plan on linkage with OUD treatment 30 days post-discharge; (3) Assess the moderating effects of patient characteristics (e.g., gender, race, ethnicity, insurance status) on post-discharge linkage. We will summarize bivariate relationships between site and patient characteristics. To evaluate how these relationships may affect endpoints, we will assess the interaction effects between site and these covariates from the logistic regression models described for the primary and secondary analyses.

Additionally, of interest is time to linkage to care following discharge. A Cox proportional hazards model will be fitted to the time to linkage with intervention arm and other relevant baseline characteristics as covariates, including age, insurance status (as a marker for income), race, and ethnicity. Additional covariates identified for the primary and secondary analyses may also be included. The proportional hazards assumption will be assessed. The relative risk and 95% CI for the two arms will reported and median times to linkage will be reported.

# 10 SUPPORTING DOCUMENTATION AND OPERATIONAL CONSIDERATIONS

## 10.1 REGULATORY, ETHICAL, AND STUDY OVERSIGHT CONSIDERATIONS

### 10.1.1 INFORMED CONSENT PROCESS

#### 10.1.1.1 CONSENT/ASSENT AND OTHER INFORMATIONAL DOCUMENTS PROVIDED TO PARTICIPANTS

Consent forms describing in detail the study intervention, study procedures, and risks will be given to the participant and documentation (e-signature) of informed consent will be completed prior to starting the study intervention. The consent form will be prepared in English and Spanish.
English and Spanish consent forms are included in Appendix 11.1.

#### 10.1.1.2 CONSENT PROCEDURES AND DOCUMENTATION

The consent process for eligible patients is as follows:

1. Approved study staff will provide the full consent form and review the consent summary (see Appendix 11.1) with patient.
2. Consent will be obtained in-person either on paper or electronically using an approved platform or remotely using an approved platform.
3. When signing electronically, patients will be offered the option of receiving a copy of the signed and dated consent form by encypted email. Otherwise, they will be given a paper copy.
4. Approved study staff will enroll consented, eligible patients by assigning a study ID in the REDCap project.

The consent form will be translated into Spanish. For Spanish-speaking participants, Spanish-speaking research staff or English-speaking staff with a Spanish-speaking interpreter will obtain consent.

### 10.1.2 STUDY DISCONTINUATION AND CLOSURE

This study may be temporarily suspended or prematurely terminated if there is sufficient reasonable cause. Written notification, documenting the reason for study suspension or termination, will be provided by the suspending or terminating party to study participants, investigators, and funding agencies.

If the study is prematurely terminated or suspended, the lead Principal Investigator (PI) (Danovitch) will promptly inform study participants, the Institutional Review Board (IRB), and sponsor/funding agencies and will provide the reason(s) for the termination or suspension. Study participants will be contacted, as applicable, and be informed of changes to study visit schedule.

Circumstances that may warrant termination or suspension include, but are not limited to:

- Determination of unexpected, significant, or unacceptable risk to participants
- Insufficient compliance of study staff to the protocol (i.e., significant protocol violations)
- Data that are not sufficiently complete and/or evaluable
- Determination that the primary endpoint has been met

The study may resume once concerns about safety, protocol compliance, and data quality are addressed, and satisfy the funding agencies, sponsor, IRB, DSMB.

### 10.1.3 CONFIDENTIALITY AND PRIVACY

Participant confidentiality and privacy is strictly held in trust by the participating investigators, their staff, and the DMSB and funding agencies. This confidentiality is extended to the data being collected as part of this study. Data that could be used to identify a specific study participant will be held in strict confidence within the research team. No personally identifiable information from the study will be released to any unauthorized third party without prior written approval of the sponsor/funding agency.

All research activities will be conducted in as private a setting as possible.

**Measures Taken to Ensure Confidentiality of Data Shared per the NIH Data Sharing Policies**
It is NIH policy that the results and accomplishments of the activities that it funds should be made available to the public (see *https://grants.nih.gov/policy/sharing.htm*). The PIs will ensure all mechanisms used to share data will include proper plans and safeguards for the protection of privacy, confidentiality, and security for data dissemination and reuse (e.g., all data will be thoroughly de-identified and will not be traceable to a specific study participant). Plans for archiving and long-term preservation of the data will be implemented, as appropriate.

**Certificate of Confidentiality**

To further protect the privacy of study participants, the Secretary, Health and Human Services (HHS), has issued a Certificate of Confidentiality (CoC) to all researchers engaged in biomedical, behavioral, clinical or other human subjects research funded wholly or in part by the federal government. Recipients

of NIH funding for human subjects research are required to protect identifiable research information from forced disclosure per the terms of the NIH Policy (see *https://humansubjects.nih.gov/coc/index*). As set forth in 45 CFR Part 75.303(a) and NIHGPS Chapter 8.3, recipients conducting NIH-supported research covered by this Policy are required to establish and maintain effective internal controls (e.g., policies and procedures) that provide reasonable assurance that the award is managed in compliance with Federal statutes, regulations, and the terms and conditions of award. It is the NIH policy that investigators and others who have access to research records will not disclose identifying information except when the participant consents or in certain instances when federal, state, or local law or regulation requires disclosure. NIH expects investigators to inform research participants of the protections and the limits to protections provided by a Certificate issued by this Policy.

### 10.1.4 FUTURE USE OF STORED SPECIMENS AND DATA

Data collected for this study will be analyzed and stored at UNM SDCC. After the study is completed, de-identified, archived data will be transmitted to and stored at each site for future analyses. Data will not be stored in a central repository. UNM will store all identifiable study data on their secured servers for up to seven years following completion of the START study, per UNM and federal policies, and will be destroyed at that time or according to NIH guidelines. Each site may retain their site-specific, identified data on their own password-protected, secured servers behind their institution's firewall for additional analyses.

### 10.1.5 KEY ROLES AND STUDY GOVERNANCE

**Principal Investigators**

| **Principal Investigator (Contact)** | **Principal Investigator** |
|---|---|
| Itai Danovitch, MD, MBA (MPI) | Allison Ober, PhD, MSW (MPI) |
| Cedars-Sinai Medical Center | RAND Corporation |
| 8730 Alden Drive, Suite E-135<br>Los Angeles, CA 90048 | 1776 Main Street, Santa Monica,<br>CA 90407 |
| 310.423.2600 | 310-393-0411 ext. 6639 |
| Itai.danovitch@csmc.org | ober@rand.org |

The study has a Steering Committee that consists of the MPIs and Co-Is, workgroup leads, and other relevant study staff. The Steering Committee meets monthly to discuss issues relevant to study design, administration, and implementation. Site responsibilities and leadership are further enumerated below.

**Key Roles**

| | |
|---|---|
| **Principal Investigator (MPI - Contact)** | Itai Danovitch, MD, MBA<br>Cedars-Sinai Medical Center<br>8730 Alden Drive, Suite E-135<br>Los Angeles, CA 90048<br>Research Activities: Overall study oversight; lead clinical intervention |
| **Principal Investigator (MPI)** | Allison Ober, MSW, PhD (MPI) |

| | |
|---|---|
| | RAND Corporation, 1776 Main Street, Santa Monica, CA 90407; 310-393-0411 ext. 6639<br>Research Activities: Lead RCT implementation and evaluation; oversee organizational context analysis |
| **Sponsor/Funders** | Pablo Cure (Program Officer) NCATS National Center for Advancing Translational Sciences;<br>NIDA (National Institute on Drug Abuse) |
| **Study Sites/Statistics and Data Coordinating Center (SDCC)** | Kimberly Page, PhD, MPH (Co-I);<br>Cristina Murray-Krezan, PhD (Study Statistician)<br>University of New Mexico Health Sciences Center, MSC10 5550, 1 University of New Mexico, Albuquerque, NM 87131; (415) 350-0625<br>Research Activities: Conduct RCT. Serve as multisite statistics and data coordinating center (SDCC) - obtain EMR, patient survey and follow-up medical records data variables, and analyze RCT outcomes<br><br>Peter Friedmann, MD, MPH (Co-I)<br>Baystate Medical Center, 759 Chestnut Street, Springfield, MA 01199; (413) 794-3391<br>Research Activities: Conduct RCT |
| **CTSA** | Research Activities: Support sites as needed inpatient identification and recruitment procedures |
| **Data Safety and Monitoring Board** | UCLA Data and Safety Monitoring Board for Addiction Medicine (DSMB-AM, led by Dr. Steven Shoptaw of UCLA Department of Family Medicine).<br>Research Activities: Serve as DSMB for multisite trial |

### 10.1.6 SAFETY OVERSIGHT

The NIH requires the establishment of DSMBs for multi-site clinical trials involving interventions that entail potential risks to the participants, even trials that pose little likelihood of harm. This study's DSMB will be established through the UCLA Data and Safety Monitoring Board for Addiction Medicine (DSMB-AM, led by Steven Shoptaw).

The UCLA DSMB-AM will monitor throughout the entire course of the study, specifically during the period that involves any human involvement (e.g., enrollment stage, during course of study treatment, throughout any follow-up assessments, etc.). The DSMB-AM will conduct an initial review at the beginning of study, when the team will be applying for Institutional Review Board approval. After that, they will hold annual interim reviews. Once all data collection for human subjects is complete, the DSMB-AM will conduct a final review. The DSMB-AM will reserve the right to request additional interim reviews, in the unlikely instance that there is an increased risk during the course of the study.

The DSMB-AM includes experts in all scientific disciplines needed to interpret the data and ensure patient safety, including addition experts, clinical trial experts, biostatisticians, and bioethicists. DSMB members will have no association with the project investigators, and no conflicts of interest with study outcomes. DSMB procedures will conform with usual standards, including reviewing emerging trial data and maintaining confidentiality. DSMB members will serve the following functions: (1) reviewing the

research protocol and plans for data and safety monitoring; (2) monitoring data quality, timeliness, recruitment, retention, performance across study sites, and factors that may affect the risks and benefits of the study such as emerging literature; and (3) making recommendations regarding continuation of the trial.

### 10.1.7 CLINICAL MONITORING

N/A

### 10.1.8 QUALITY ASSURANCE AND QUALITY CONTROL

Quality control (QC) procedures will be implemented as follows:

**Informed consent** — Study staff at each site will review both the documentation of the consenting process as well as a percentage of the completed consent documents. This review will evaluate compliance with GCP, accuracy, and completeness. Feedback will be provided to the larger study team to ensure proper consenting procedures are followed.

**Intervention Fidelity** — Consistent delivery of the study interventions will be monitored throughout the intervention phase of the study. Procedures for ensuring fidelity of intervention delivery are described in **Section 6.2.1, Interventionist Training and Tracking**.

**Protocol Deviations** – The study team will review protocol deviations on an ongoing basis and will implement corrective actions when the quantity or nature of deviations are deemed to be at a level of concern. **Section 9.5.10 provides information on protocol deviations and reporting.**

**Data** – PI Page and the SDCC team at UNM will be responsible for data quality control, including evaluating data for adherence with the protocol and for accuracy. Site queries will occur every 2-4 weeks. Study status reports generated from the database will provide a basis for ongoing monitoring of subject accrual and retention, as well as completeness of data. These will be used to identify and resolve problems that may arise. Data extracted from each site's electronic medical record will be uploaded to the UNM SDCC via secure file transfer protocol (SFTP) and checked monthly during the study. Follow-up survey data will be entered directly into REDCap. Under the direction of a statistician, a quantitative analyst will check the data for completeness and accuracy.

Should independent monitoring become necessary, the PI will provide direct access to all trial-related sites, source data/documents, and reports for the purpose of monitoring and auditing by the sponsor/funding agency, and inspection by local and regulatory authorities.

### 10.1.9 DATA HANDLING AND RECORD KEEPING

#### 10.1.9.1 DATA COLLECTION AND MANAGEMENT RESPONSIBILITIES

***Source Documents***

- Source documents are:
  - REDCap eligibility form
  - REDCap electronic consent
  - REDCap baseline and follow-up survey forms (RAND SRG will enter follow-up interview data directly into REDCap)
  - REDCap contact information form
  - Medical records from providers indicating service utilization, faxed to each site then uploaded into REDCap
  - Patient tracking spreadsheet
  - Patient registry (This is part of the intervention; these records will be linked to study records using the MRN, which will be in REDCap and in the Registry.)

***Data Management***

Data will be collected from multiple sources throughout the course of the study. All prospectively collected data will be directly entered into the UNM REDCap electronic data capture system which is administered by the UNM Clinical and Translational Science Center (CTSC). The UNM Statistics and Data Coordinating Center (SDCC) will develop electronic data collection forms of the patient interviews in REDCap and will maintain access rights to the database. All data will be stored on UNM HSC's secured servers and behind their firewall. Other data sent to UNM will be transferred via SFTP following all institutional policies and executed data use agreements. Data to be curated by the UNM SDCC includes the following sources:

- Potentially eligible participants will be identified from reports generated from the electronic medical record (EMR) 3-5 times per week by the sites' informatics teams. These reports will be restricted to admitted patients who meet the eligibility criteria specified in Section 5.
- Consent forms obtained electronically may be sent to participants by encrypted email.
- Baseline and outcome interview data will be collected by Research Assistants at each site and follow-up interview data will be collected by RAND SRG by telephone. All data will be directly entered into the UNM SDCC's REDCap system.
- In the event that the UNM SDCC's REDCap system cannot be accessed, the sites may enter data in a copy of the project on their local REDCap system or utilize paper forms. In these cases, the data will be merged into the the UNM SDCC's REDCap system when it becomes available, either by site staff or by transmission to UNM SDCC via SFTP for entry into their system.
- Additional outcome data will be obtained from the sites' EMR and will be retrieved by their informatics teams. This data will be sent to the UNM SDCC via SFTP and will be merged with the other study data.

***Data Storage***

- All study data will be stored in a REDCap database behind UNM Health Science Center's (HSC) firewall on a secured and encrypted password protected server following UNM HSC data

security policies. Any identifiable information (such as contact information and medical record number) will be stored in a REDCap database ("project") in an encrypted format behind a firewall on UNM's secured servers and is accessible only to designated personnel on a case-by-case basis. The UNM data center is staffed with onsite security personnel 24x7x365 and provides multilevel physical and logical security protection including: monitoring, video surveillance, biometric and access card and man-trap access to datacenter floor, encrypted and password protected servers, and restricted logical access, with a dedicated and encrypted data drive behind a firewall. Access to the data is provided only to authorized users through an encrypted transmission channel with a password-protected application interface. UNM will store all data on their secured servers for up to 7 years following completion of the START study, per UNM policies, and will be destroyed at that time or according to NIH guidelines. Should medical record data need to be received by the SDCC via fax, it will be uploaded to REDCap and paper copies will be destroyed. Should paper collection of study data or forms need to be utilized, they will be destroyed once they are confirmed to be in the electronic system. Additionally, all deidentified analysis data sets will be transmitted via SFTP to the Principal Investigators at the participating sites for additional analyses where the data will be stored behind their institution's firewalls on password-protected and encrypted servers.

***Responsibility for Data Safety and Monitoring***

The study team will monitor the safety of participants and the validity and integrity of the data. Data safeguarding procedures will adhere to standards established by applicable regulations including those by the NIH, the Department of Health and Human Services, local HIPAA regulations, and standards set by the Cedars-Sinai Institutional Review Board.

As Co-PIs, Drs. Itai Danovitch and Allison Ober carry ultimate responsibility for ensuring compliance with the trial Protocol as well as the Data and Safety Monitoring Plan. Dr. Nuckols at Cedars-Sinai will be tasked with overseeing compliance with procedures for human subjects' protections as well as data and safety monitoring, under the guidance of Drs. Ober and Danovitch. Researchers at the University of New Mexico SDCC will be responsible for monitoring the data quality and completeness, and ensuring the integrity of descriptive and statistical analyses. All of these investigators have extensive experience in handling sensitive information.

To monitor data and safety issues, this team will meet regularly throughout the study project, enabling any concerns to be addressed rapidly. We will monitor reasons that patients give for declining to participate in the study, as well as reasons for stopping their study participation after initially giving consent. Drs. Ober and Danovitch will report to the Cedars-Sinai Institutional Review Board, the single IRB for the project, through annual progress reports. Site investigators also will report to their local IRB, as required by each IRB.

#### 10.1.9.2 STUDY RECORDS RETENTION

All study records will be retained for a minimum of 7 years (as required for NIH-funded studies) after the formal discontinuation the study intervention. These documents will be retained for a longer period, however, if required by local regulations. For research data and materials involving Protected Health Information (PHI), the PI must retain the signed consent forms that contain the permission to use the PHI for six (6) years beyond the expiration date of the authorization (i.e. the consent form or authorization).

### 10.1.10 PROTOCOL DEVIATIONS

This protocol defines a protocol deviation (PD) as any noncompliance with the clinical trial protocol, International Council on Harmonisation Good Clinical Practice (ICH GCP), Protocol) requirements. The noncompliance may be either on the part of the participant, the investigator, or the study site staff. As a result of deviations, corrective actions will be developed by the site and implemented promptly. Examples of protocol deviations may include enrollment of an ineligible subject, follow-up interview conducted out-of-window, or a START member seeing a usual care patient.

These practices are consistent with ICH GCP:

- Section 4.5 Compliance with Protocol, subsections 4.5.1, 4.5.2, and 4.5.3
- Section 5.1 Quality Assurance and Quality Control, subsection 5.1.1
- Section 5.20 Noncompliance, subsections 5.20.1, and 5.20.2.

PDs will be recorded by each site's study coordinator in the REDCap Reporting Form. Each site PI and/or clinical designee will review the form and investigate each event further to ascertain: (1) nature of the event (subject eligibility, consent process), (2) type and degree of potential or actual harm (social/psychological), (3) potential contributing factors, and (4) potential for recurrence. The research team will confer on how to handle the events, including discussing them with the IRB, if uncertainty exists. Further details about the handling of protocol deviations are included in the MOP.

Completed PD Forms will be automatically sent through REDCap to Dr. Danovitch and the CSMC study coordinator, who will review and notify the Cedars-Sinai IRB, the DSMB, and the study Project Officer as required and outlined below.

**IRB reporting:** The site PIs and Dr. Danovitch, will assist in determining whether the deviation must be reported to the Cedars-Sinai IRB. A minor logistical deviation (e.g., an out-of-window study visit or procedure, a missed weekly phone check-in) does not need to be reported to the IRB. A protocol deviation must be reported to the IRB if the study investigators determine it poses a threat to the integrity of the study or if the deviation impacts participant rights, safety, or welfare. For example, deviations related to subject eligibility or consent procedures (e.g., patient enrolled despite meeting an exclusion criterion, consent not obtained prior to study start, consent obtained by unauthorized individual, incorrect version of consent form used) must be reported. Reportable events must be reported by the sites to the CSMC study team as soon as possible but no later than 10 business days from the relying site's awareness of the event. The CSMC study team must submit the event in CS-IRB as

soon as possible but no later than 10 business days from the CSMC study team's awareness of the event, via a Reportable New Information (RNI) form in CS-IRB, selecting the most appropriate type of report, and including copies of any relevant documentation.

In the event that the protocol exception needs to be requested to accommodate a single subject, the CSMC IRB must be notified in advance. Deviations made to avoid immediate hazard to a participant must be reported to the CSMC IRB within 5 business days. Unintentional protocol deviations must be reported within 10 business days.

**DSMB reporting:** All PDs will be summarized and reported to the DSMB at three timepoints: At three time points during data collection, when the enrollment reaches one-third (N=137), two-thirds (N=272), and upon completion (N=414).

**NIH reporting:** PDs that require IRB reporting (as described above) will be reported to the project officer by email within 10 business days.

### 10.1.11 PUBLICATION AND DATA SHARING POLICY

This study will be conducted in accordance with the following publication and data sharing policies and regulations:

National Institutes of Health (NIH) Public Access Policy, which ensures that the public has access to the published results of NIH funded research. It requires scientists to submit final peer-reviewed journal manuscripts that arise from NIH funds to the digital archive PubMed Central upon acceptance for publication.

This study will comply with the NIH Data Sharing Policy and Policy on the Dissemination of NIH-Funded Clinical Trial Information and the Clinical Trials Registration and Results Information Submission rule. As such, this trial will be registered at ClinicalTrials.gov, and results information from this trial will be submitted to ClinicalTrials.gov. In addition, every attempt will be made to publish results in peer-reviewed journals. Data from this study may be requested from other researchers after the completion of the primary endpoint by contacting the PIs. Reasonable request for sharing data will be considered by the PIs (see Publications and Dissemination Policies and Procedures). Considerations for ensuring confidentiality of these shared data are described in Section 10.1.3.

### 10.1.12 CONFLICT OF INTEREST POLICY

The independence of this study from any actual or perceived influence, such as by the pharmaceutical industry, is critical. Therefore, any actual conflict of interest of persons who have a role in the design, conduct, analysis, publication, or any aspect of this trial will be disclosed and managed. Furthermore, persons who have a perceived conflict of interest will be required to have such conflicts managed in a way that is appropriate to their participation in the design and conduct of this trial. The study leadership in conjunction with the National Center for Advancing Translational Sciences (NCATS) has established

policies and procedures for all study group members to disclose all conflicts of interest and will establish a mechanism for the management of all reported dualities of interest.

## 10.2 ADDITIONAL CONSIDERATIONS

N/A

## 10.3 ABBREVIATIONS AND SPECIAL TERMS

| | |
|---|---|
| AMS | Addiction Medicine Specialist |
| ASSIST | Alcohol, Smoking, and Substance Involvement Screening test |
| BH | Baystate Health |
| BNI | brief negotiated interview |
| CCT | collaborative care team |
| CFIR | Consolidated Framework for Implementation Research |
| CIs | confidence intervals |
| CM | Care Manager |
| CSMC | Cedars-Sinai Medical Center |
| DRGs | Diagnosis Related Group |
| DSMB | Data and Safety Monitoring Board |
| EMR | electronic medical record |
| GAD | Generalized anxiety disorder |
| GAIN | Global Appraisal of Individual Needs |
| IPW-MI | inverse probability weighting and multiple imputation |
| MRN | medical records number |
| MOUD | medication for opioid use disorder |
| NSDUH | National Survey of Drug Use and Health |
| NIDA | National Institute on Drug Abuse |
| OUD | opioid use disorders |
| OR | odds ratio |
| PACIC | Patient Assessment of Chronic Illness Care |
| PEG | pain intensity (P), interference with enjoyment of life (E), and interference with general activity (G) |
| PHQ | Patient Health Questionnaire |
| RED | re-engineered discharge |
| REDCap | Research Electronic Data Capture |
| START | Substance Use Treatment and Recovery Team |
| SIP-AD | Short Inventory of Problems Alcohol and Drugs |
| SDCC | Statistics and Data Coordinating Center |
| SFTP | Secure File Transfer Protocol |
| SASS | Substance Abuse Stigma Scale |
| UC | Usual Care |
| UCLA | University of California, Los Angeles |

| | |
|---|---|
| UNM | University of New Mexico |
| WHO | World Health Organization |

## 10.4 PROTOCOL AMENDMENT HISTORY

*The table below is intended to capture changes of IRB-approved versions of the protocol, including a description of the change and rationale.*

**Table 12. Protocol Amendment History**

| Version | Date | Description of Change | Brief Rationale |
|---|---|---|---|
| 1.2 | 10/21/2021 | Process for sending participants a copy of the electronic consent form by encrypted email | Ensure accurate description of process |
| 1.3 | 02/02/2021 | Follow-up period extended from 30 to 60 days; for participants in the intervention arm who are discharged prior to intervention initiation, intervention will be delivered telephoincally | Retention improvement; allow flexibility of intervention delivery |
| 1.4 | 04/21/2022 | Use of paper forms in the event that electronic data capture is not accessible | Allow flexibility in the event of Internet or database outage |
| 1.5 | 06/15/2022 | Optional, rather than required, validation of self-reported outcome measures with providers; increase follow-up incentive | Difficulty in validating outcome measures with providers; retention improvement |
| 1.6 | 10/27/2022 | Update includion criteria to clarify that "inpatient" also refers to patients under observation | Expand eligibility criteria to be more inclusive |
| 1.7 | 08/08/2023 | Describe planned interim analysis | Determine if current sample size is sufficient to analyze second primary outcome |

# 11 APPENDICES

(May be found as separate documents as listed below.)

## 11.1 RECRUITMENT AND ENROLLMENT

- Consent
- Consent Checklist
- Eligibility Screener
- Eligibility Checklist
- Study Physician Flyer
- Physician Screening Card

- Baystate and UNM: Physician to Patient Letter, Physician Recruitment Letter with FAQs about START
- CSMC: Direct-to-Patient Recruitment Letter, Physician Recruitment Letter with FAQs about START
- Patient & Family Education Materials

## 11.2 EVALUATION

- Baseline Survey
- Follow-up Survey

## 11.3 DATA SAFETY AND MONITORING

- Adverse Event Form
- Adverse Event Summary Form
- List of “expected” AE
- Protocol Deviation Form

# 12 REFERENCES


1. Ronan MV, Herzig SJ. Hospitalizations Related To Opioid Abuse/Dependence And Associated Serious Infections Increased Sharply, 2002-12. *Health Aff (Millwood).* May 01 2016;35(5):832-837. PMC5240777.
2. Fanucchi L, Lofwall MR. Putting Parity into Practice - Integrating Opioid-Use Disorder Treatment into the Hospital Setting. *N Engl J Med.* Sep 1 2016;375(9):811-813.
3. Rosenthal ES, Karchmer AW, Theisen-Toupal J, Castillo RA, Rowley CF. Suboptimal addiction interventions for patients hospitalized with injection drug use-associated infective endocarditis. *Am. J. Med.* May 2016;129(5):481-485.
4. Reif S, Acevedo A, Garnick DW, Fullerton CA. Reducing Behavioral Health Inpatient Readmissions for People With Substance Use Disorders: Do Follow-Up Services Matter? *Psychiatr Serv.* Aug 1 2017;68(8):810-818.
5. Nordeck CD, Welsh C, Schwartz RP, et al. Rehospitalization and substance use disorder (SUD) treatment entry among patients seen by a hospital SUD consultation-liaison service. *Drug Alcohol Depend.* May 1 2018;186:23-28.
6. Walley AY, Paasche-Orlow M, Lee EC, et al. Acute care hospital utilization among medical inpatients discharged with a substance use disorder diagnosis. *J Addict Med.* Mar 2012;6(1):50-56.
7. Hsu DJ, McCarthy EP, Stevens JP, Mukamal KJ. Hospitalizations, costs and outcomes associated with heroin and prescription opioid overdoses in the United States 2001-12. *Addiction.* Sep 2017;112(9):1558-1564.
8. Gupta A, Nizamuddin J, Elmofty D, et al. Opioid abuse or dependence increases 30-day readmission rates after major operating room procedures: A national readmissions database study. *Anesthesiology.* May 2018;128(5):880-890.
9. Binswanger IA. Commentary on Hsu et al. (2017): A systems approach to improving health services for overdose in the hospital and across the continuum of care-an unmet need. *Addiction.* Sep 2017;112(9):1565-1566.
10. Stewart S, Swain S. Assessment and management of alcohol dependence and withdrawal in the acute hospital: concise guidance. *Clin Med.* Jun 2012;12(3):266-271.
11. Wakeman SE, Kanter GP, Donelan K. Institutional substance use disorder intervention improves general internist preparedness, attitudes, and clinical practice. *J Addict Med.* Jul/Aug 2017;11(4):308-314.
12. Watkins KE, Ober AJ, Lamp K, et al. Implementing the chronic care model for opioid and alcohol use disorders in primary care. *Progress in Community Health Partnerships: Research, Education, and Action.* 2017;11(4):397-407. PMCID: PMC6124482.
13. Watkins KE, Ober AJ, Lamp K, et al. Collaborative care for opioid and alcohol use disorders in primary care: The SUMMIT randomized clinical trial. *JAMA Intern Med.* Oct 1 2017;177(10):1480-1488. PMCID: PMC5710213.
14. Hunt P, Ober AJ, Watkins KE. *The costs of implementing collaborative care for opioid and alcohol use disorders in primary care (RR-2049-NIDA).* Santa Monica, CA: RAND Corporation;2017.
15. Hunter SB, Ober AJ, McCullough CM, et al. Sustaining alcohol and opioid use disorder treatment in primary care: A mixed methods study. *Implementation Science.* 2018;13:83. PMCID: PMC6006923.

16. Ober AJ, Watkins KE, Hunter SB, et al. Assessing and improving organizational readiness to implement substance use disorder treatment in primary care: Findings from the SUMMIT study. *BMC Fam Pract.* Dec 21 2017;18(1):107. PMCID: PMC5740845.
17. American Hospital Association. Behavioral Health Task Force report. 2017.
18. Bukberg J, Penman D, Holland JC. Depression in hospitalized cancer patients. *Psychosom Med.* May-Jun 1984;46(3):199-212.
19. Freedland KE, Rich MW, Skala JA, Carney RM, Dávila-Román VG, Jaffe AS. Prevalence of Depression in Hospitalized Patients With Congestive Heart Failure. *Psychosomatic Medicine.* 2003;65(1).
20. Hsu DJ, McCarthy EP, Stevens JP, Mukamal KJ. Hospitalizations, costs and outcomes associated with heroin and prescription opioid overdoses in the United States 2001-12. *Addiction (Abingdon, England).* 2017;112(9):1558-1564.
21. Martucci M, Balestrieri M, Bisoffi G, et al. Evaluating psychiatric morbidity in a general hospital: a two-phase epidemiological survey. *Psychol Med.* Jul 1999;29(4):823-832.
22. Ronan MV, Herzig SJ. Hospitalizations Related To Opioid Abuse/Dependence And Associated Serious Infections Increased Sharply, 2002-12. *Health affairs (Project Hope).* 2016;35(5):832-837.
23. Silverstone PH. Prevalence of psychiatric disorders in medical inpatients. *J Nerv Ment Dis.* Jan 1996;184(1):43-51.
24. Roberto P, Brandt N, Onukwugha E, Perfetto E, Powers C, Stuart B. Adherence to Antipsychotic Therapy: Association With Hospitalization and Medicare Spending Among Part D Enrollees With Schizophrenia. *Psychiatr Serv.* Nov 1 2017;68(11):1185-1188.
25. Wu LT, Zhu H, Swartz MS. Treatment utilization among persons with opioid use disorder in the United States. *Drug Alcohol Depend.* Dec 1 2016;169:117-127. PMC5223737.
26. Pan Y-J, Lee M-B, Chiang H-C, Liao S-C. The recognition of diagnosable psychiatric disorders in suicide cases' last medical contacts. *General hospital psychiatry.* 2009 Mar-Apr 2009;31(2):181-184.
27. Shei A, Rice JB, Kirson NY, et al. Characteristics of High-Cost Patients Diagnosed with Opioid Abuse. *Journal of managed care & specialty pharmacy.* 2015/10// 2015;21(10):902-912.
28. Wakeman SE, Pham-Kanter G, Donelan K. Attitudes, practices, and preparedness to care for patients with substance use disorder: Results from a survey of general internists. *Subst Abus.* Oct-Dec 2016;37(4):635-641.
29. Hussain M, Seitz D. Integrated models of care for medical inpatients with psychiatric disorders: A systematic review. *Psychosomatics.* Jul-Aug 2014;55(4):315-325.
30. Institute of Medicine. *Improving the quality of health care for mental and substance-use conditions: Quality chasm series. Appendix B, constraints on sharing mental health and substance-use treatment information imposed by federal and state medical records privacy laws.* Washington, DC: National Academies Press;2006.
31. Raven MC, Carrier ER, Lee J, Billings JC, Marr M, Gourevitch MN. Substance use treatment barriers for patients with frequent hospital admissions. *J Subst Abuse Treat.* Jan 2010;38(1):22-30.
32. Kopera M, Suszek H, Bonar E, et al. Evaluating explicit and implicit stigma of mental illness in mental health professionals and medical students. *Community Mental Health Journal.* July 01 2015;51(5):628-634.
33. Stull LG, McGrew JH, Salyers MP, Ashburn-Nardo L. Implicit and explicit stigma of mental illness: Attitudes in an evidence-based practice. *J Nerv Ment Dis.* Dec 2013;201(12):1072-1079.

34. Owens P, Barrett M, Weiss A, Kronick R. *Hospital inpatient utilization related to opioid overuse among adults, 1993-2012. Vol HCUP statistical brief #177.* Rockville, MD: Agency for Healthcare Research and Quality;2014.
35. Bahorik AL, Satre DD, Kline-Simon AH, Weisner CM, Campbell CI. Alcohol, cannabis, and opioid use disorders, and disease burden in an integrated health care system. *J Addict Med.* Jan/Feb 2017;11(1):3-9.
36. Larochelle MR, Bernson D, Land T, et al. Medication for opioid use disorder after nonfatal opioid overdose and association with mortality: A cohort study. *Ann Intern Med.* Jun 19 2018.
37. Volkow ND, Frieden TR, Hyde PS, Cha SS. Medication-assisted therapies--tackling the opioid-overdose epidemic. *N Engl J Med.* May 29 2014;370(22):2063-2066.
38. Clark RE, Baxter JD, Aweh G, O'Connell E, Fisher WH, Barton BA. Risk factors for relapse and higher costs among Medicaid members with opioid dependence or abuse: Opioid agonists, comorbidities, and treatment history. *J Subst Abuse Treat.* Oct 2015;57:75-80.
39. Naeger S, Mutter R, Ali MM, Mark T, Hughey L. Post-discharge treatment engagement among patients with an opioid-use disorder. *J Subst Abuse Treat.* Oct 2016;69:64-71.
40. Center for Behavioral Health Statistics and Quality (CBHSQ). Results from the 2015 National Survey on Drug Use and Health: detailed tables. 2016; *http://www.samhsa.gov/data/sites/default/files/NSDUH-DetTabs-2015/NSDUH-DetTabs-2015/NSDUH-DetTabs-2015.pdf*. Accessed July 6, 2018.
41. Wu L-T, Zhu H, Swartz MS. Treatment utilization among persons with opioid use disorder in the United States. *Drug and alcohol dependence.* 10/19 2016;169:117-127.
42. Tsui JI, Evans JL, Lum PJ, Hahn JA, Page K. Association of opioid agonist therapy with lower incidence of hepatitis C virus infection in young adult injection drug users. *JAMA Intern Med.* Dec 2014;174(12):1974-1981. PMCID: PMC4506774.
43. Pecoraro A, Horton T, Ewen E, et al. Early data from Project Engage: a program to identify and transition medically hospitalized patients into addictions treatment. *Addict Sci Clin Pract.* Sep 25 2012;7:20. PMC3507657.
44. Pollini RA, O'Toole TP, Ford D, Bigelow G. Does this patient really want treatment? Factors associated with baseline and evolving readiness for change among hospitalized substance using adults interested in treatment. *Addict Behav.* Oct 2006;31(10):1904-1918.
45. Velez CM, Nicolaidis C, Korthuis PT, Englander H. "It's been an experience, a life learning experience": A qualitative study of hospitalized patients with substance use disorders. *J Gen Intern Med.* Mar 2017;32(3):296-303.
46. Englander H, Weimer M, Solotaroff R, et al. Planning and designing the Improving Addiction Care Team (IMPACT) for hospitalized adults with substance use disorder. *Journal of Hospital Medicine.* 2017;12(5):339-342.
47. Huhn AS, Tompkins DA, Dunn KE. The relationship between treatment accessibility and preference amongst out-of-treatment individuals who engage in non-medical prescription opioid use. *Drug Alcohol Depend.* Nov 1 2017;180:279-285.
48. Alfandre DJ. "I'm going home": Discharges against medical advice. *Mayo Clin Proc.* Mar 2009;84(3):255-260.
49. Ti L, Milloy MJ, Buxton J, et al. Factors associated with leaving hospital against medical advice among people who use illicit drugs in Vancouver, Canada. *PLoS One.* 2015;10(10):e0141594.
50. Sledge W, Bozzo J, White-McCullum B, Lee H. The cost-benefit from the perspective of the hospital of a proactive psychiatric consultation service on inpatient general medicine services. *Health Economics & Outcome Research: Open Access.* 2016;2(122):2.

51. Sledge WH, Gueorguieva R, Desan P, Bozzo JE, Dorset J, Lee HB. Multidisciplinary proactive psychiatric consultation service: Impact on length of stay for medical inpatients. *Psychother Psychosom.* 2015;84(4):208-216.
52. Muskin PR, Skomorowsky A, Shah RN. Co-managed care for medical inpatients, C-L vs C/L psychiatry. *Psychosomatics.* May-Jun 2016;57(3):258-263.
53. Mayo AT, Williams Woolley A. Teamwork in health care: Maximizing collective intelligence via inclusive collaboration and open communication. *AMA Journal of Ethics.* 2016;18(9):933-940.
54. Whiteside LK, Darnell D, Jackson K, et al. Collaborative care from the emergency department for injured patients with prescription drug misuse: An open feasibility study. *J Subst Abuse Treat.* Nov 2017;82:12-21.
55. Zatzick D, Roy-Byrne P, Russo J, et al. A randomized effectiveness trial of stepped collaborative care for acutely injured trauma survivors. *Arch Gen Psychiatry.* May 2004;61(5):498-506.
56. Zatzick DF, Koepsell T, Rivara FP. Using target population specification, effect size, and reach to estimate and compare the population impact of two PTSD preventive interventions. *Psychiatry.* Winter 2009;72(4):346-359.
57. Zatzick DF, Roy-Byrne P, Russo JE, et al. Collaborative interventions for physically injured trauma survivors: A pilot randomized effectiveness trial. *Gen Hosp Psychiatry.* May-Jun 2001;23(3):114-123.
58. Zatzick DF, Russo J, Darnell D, et al. An effectiveness-implementation hybrid trial study protocol targeting posttraumatic stress disorder and comorbidity. *Implementation Science.* Apr 30 2016;11:58.
59. Trowbridge P, Weinstein ZM, Kerensky T, et al. Addiction consultation services - Linking hospitalized patients to outpatient addiction treatment. *J. Subst. Abuse Treat.* Aug 2017;79:1-5.
60. Wakeman SE, Metlay JP, Chang Y, Herman GE, Rigotti NA. Inpatient addiction consultation for hospitalized patients increases post-discharge abstinence and reduces addiction severity. *J Gen Intern Med.* Aug 2017;32(8):909-916.
61. Liebschutz JM, Crooks D, Herman D, et al. Buprenorphine treatment for hospitalized, opioid-dependent patients: a randomized clinical trial. *JAMA internal medicine.* Aug 2014;174(8):1369-1376. Pmc4811188.
62. Archer J, Bower P, Gilbody S, et al. Collaborative care for depression and anxiety problems. *Cochrane Database Syst Rev.* Oct 17 2012;10:Cd006525.
63. Huang Y, Wei X, Wu T, Chen R, Guo A. Collaborative care for patients with depression and diabetes mellitus: A systematic review and meta-analysis. *BMC Psychiatry.* Oct 14 2013;13:260.
64. Huffman JC, Niazi SK, Rundell JR, Sharpe M, Katon WJ. Essential articles on collaborative care models for the treatment of psychiatric disorders in medical settings: A publication by the Academy of Psychosomatic Medicine Research and Evidence-Based Practice Committee. *Psychosomatics.* Mar-Apr 2014;55(2):109-122.
65. Tully PJ, Baumeister H. Collaborative care for comorbid depression and coronary heart disease: A systematic review and meta-analysis of randomised controlled trials. *BMJ Open.* Dec 21 2015;5(12):e009128.
66. Arean PA, Ayalon L, Hunkeler E, et al. Improving depression care for older, minority patients in primary care. *Med Care.* Apr 2005;43(4):381-390.
67. Ell K, Aranda MP, Xie B, Lee PJ, Chou CP. Collaborative depression treatment in older and younger adults with physical illness: Pooled comparative analysis of three randomized clinical trials. *Am J Geriatr Psychiatry.* Jun 2010;18(6):520-530.

68. Ell K, Katon W, Cabassa LJ, et al. Depression and diabetes among low-income Hispanics: Design elements of a socioculturally adapted collaborative care model randomized controlled trial. *Int J Psychiatry Med.* 2009;39(2):113-132.
69. Ell K, Katon W, Xie B, et al. Collaborative care management of major depression among low-income, predominantly Hispanic subjects with diabetes: A randomized controlled trial. *Diabetes Care.* Apr 2010;33(4):706-713.
70. Miranda J, Duan N, Sherbourne C, et al. Improving care for minorities: Can quality improvement interventions improve care and outcomes for depressed minorities? Results of a randomized, controlled trial. *Health Serv Res.* Apr 2003;38(2):613-630.
71. Miranda J, Schoenbaum M, Sherbourne C, Duan N, Wells K. Effects of primary care depression treatment on minority patients' clinical status and employment. *Arch Gen Psychiatry.* Aug 2004;61(8):827-834.
72. Adib-Hajbaghery M, Maghaminejad F, Abbasi A. The role of continuous care in reducing readmission for patients with heart failure. *Journal of caring sciences.* Dec 2013;2(4):255-267. 4134146.
73. Naylor MD, Brooten D, Campbell R, et al. Comprehensive discharge planning and home follow-up of hospitalized elders - A randomized clinical trial. *Jama-J Am Med Assoc.* Feb 17 1999;281(7):613-620.
74. Bielaszka-DuVernay C. Redesigning acute care processes in Wisconsin. *Health Affairs.* Mar 2011;30(3):422-425.
75. Davenport S, Matthews K. *Milliman White Paper: Opioid use disorder in the United States: Diagnosed prevalence by payer, age, sex, and state.* Seattle, WA: Milliman;2018.
76. Rogers E. *Diffusion of innovations, 4th edition.* New York, NY: The Free Press; 1995.
77. Bao Y DB, Jung HY, Chan YF, Unützer J. . Unpacking Collaborative Care for Depression: Examining Two Essential Tasks for Implementation. *Psychiatr Serv.* 2016;67:418-424.
78. Leiter RE YM, Hasdianda MA, et al. Fidelity and Feasibility of a Brief Emergency Department Intervention to Empower Adults With Serious Illness to Initiate Advance Care Planning Conversations *J Pain Symptom Manage. 2018;.* 2018:878-885.
79. Jack BW, Chetty VK, Anthony D, et al. A reengineered hospital discharge program to decrease rehospitalization: a randomized trial. *Ann Intern Med.* Feb 3 2009;150(3):178-187. PMCID: PMC2738592.
80. Agency for Healthcare Research and Quality. Re-Engineered Discharge (RED) Toolkit. 2020; *https://www.ahrq.gov/patient-safety/settings/hospital/red/toolkit/index.html*.
81. Center for Behavioral Health Statistics and Quality. *2019 National Survey on Drug Use and Health (NSDUH): CAI Specifications for Programming (English Version).* Rockville, MD: Substance Abuse and Mental Health Services Administration;2018.
82. Gelaye B, Tadesse MG, Williams MA, Fann JR, Vander Stoep A, Andrew Zhou XH. Assessing validity of a depression screening instrument in the absence of a gold standard. *Ann Epidemiol.* Jul 2014;24(7):527-531. PMC4104527.
83. Kroenke K, Spitzer RL, Williams J. The PHQ-9: Validity of a brief depression severity measure. *J Gen Intern Med.* Sep 2001;16(9):606-613. PMC1495268.
84. Löwe B, Decker O, Müller S, et al. Validation and standardization of the Generalized Anxiety Disorder Screener (GAD-7) in the general population. *Med Care.* 2008;46(3):266-274.
85. Ruiz M, Zamorano E, García-Campayo J, Pardo A, Freire O, Rejas J. Validity of the GAD-7 scale as an outcome measure of disability in patients with generalized anxiety disorders in primary care. *J Affect Disord.* 2011;128(3):277-286.

86. Spitzer R, Kroenke K, Williams J, B L. A brief measure for assessing generalized anxiety disorder: the GAD-7. *Arch Intern Med.* 2006;166(10):1092-1097.
87. Zimet GD, Powell SS, Farley GK, Werkman S, Berkoff KA. Psychometric characteristics of the Multidimensional Scale of Perceived Social Support. *J Pers Assess.* Winter 1990;55(3-4):610-617.
88. Krebs EE, Lorenz KA, Bair MJ, et al. Development and initial validation of the PEG, a three-item scale assessing pain intensity and interference. *J Gen Intern Med.* Jun 2009;24(6):733-738. PMC2686775.
89. M. D. Global Appraisal of Individual Needs (GAIN) : Administration Guide for the GAIN and Related Measures. . Bloomington, IL Chestnut Health Systems.; 2003.
90. Friedmann PD, Wilson D, Knudsen HK, et al. Effect of an organizational linkage intervention on staff perceptions of medication-assisted treatment and referral intentions in community corrections. *J Subst Abuse Treat.* Mar 2015;50:50-58. PMC4304936.
91. Grosso AL, Ketende SC, Stahlman S, et al. Development and reliability of metrics to characterize types and sources of stigma among men who have sex with men and female sex workers in Togo and Burkina Faso. *BMC infectious diseases.* 2019/03/05 2019;19(1):208.
92. Glasgow RE, Wagner EH, Schaefer J, Mahoney LD, Reid RJ, Greene SM. Development and validation of the Patient Assessment of Chronic Illness Care (PACIC). *Med Care.* May 2005;43(5):436-444.
93. Agency for Healthcare Research and Quality. CAHPS ECHO Survey Measures 2018; *https://www.ahrq.gov/cahps/surveys-guidance/echo/about/survey-measures.html*. Accessed June 23, 2021.
94. Cushman PA, Liebschutz JM, Anderson BJ, Moreau MR, Stein MD. Buprenorphine initiation and linkage to outpatient buprenorphine do not reduce frequency of injection opiate use following hospitalization. *J. Subst. Abuse Treat.* Sep 2016;68:68-73.
95. Lee CS, Liebschutz JM, Anderson BJ, Stein MD. Hospitalized opioid-dependent patients: Exploring predictors of buprenorphine treatment entry and retention after discharge. *Am. J. Addict.* Oct 2017;26(7):667-672.
96. The Comprehensive R Archive Network. Power analysis functions along the lines of Cohen (1988). undated; *https://CRAN.R-project.org/package=pwr*. Accessed July 9, 2018.
97. PASS 14. *Power analysis and sample size software.* Kaysville, Utah, USA: NCSS, LLC;2015.
98. R Core Team. *R: A language and environment for statistical computing.* Vienna, Austria: R Foundation for Statistical Computing;2017.
99. Seaman SR, White IR, Copas AJ, Li L. Combining multiple imputation and inverse-probability weighting. *Biometrics.* Mar 2012;68(1):129-137.
100. DeMets DL, Lan K. K. . Interim analysis: the alpha spending function approach. *Stat Med.* July 15-30 1994;13(13-14):1341-1356; discussion 1353-1346.
101. Lan KK, DeMets DL. Further comments on the alpha-spending function. *Stat Biosci.* May 2009;1:95-111.
102. Grubbs KM, Cheney AM, Fortney JC, et al. The role of gender in moderating treatment outcome in collaborative care for anxiety. *Psychiatr. Serv.* Mar 1 2015;66(3):265-271.

# Statistical Analysis Plan

| | |
|---|---|
| OFFICIAL GRANT TITLE | Collaborative Care Teams for Hospitalized Patients with Opioid Use Disorders |
| STUDY TITLE | Substance Use Treatment and Recovery Team (START) |
| NCT IDENTIFIED NUMBER | NCT05086796 |
| FUNDED BY | National Center for Advancing Translational Sciences & National Institute on Drug Abuse |
| GRANT NUMBER | 1U01TR002756-01A1 |
| SAP VERSION | 3.0 |
| SAP VERSION DATE | December 10, 2024 |
| TRIAL STATISTICIAN | Cristina Murray-Krezan, PhD |
| PROTOCOL VERSION (SAP associated with) | 1.7 |
| TRIAL PRINCIPAL INVESTIGATORS | Itai Danovitch, MD, Cedars-Sinai Medical Center<br>Allison Ober, PhD, RAND Corporation |
| SAP AUTHOR(s) | Cristina Murray-Krezan, PhD |

TABLE OF CONTENTS

1 ABBREVIATIONS AND DEFINITIONS ..... 4

2 INTRODUCTION ..... 5

3 STUDY OBJECTIVES AND ENDPOINTS ..... 5

3.1 Study Objectives ..... 5

3.2 Endpoints ..... 5

4 STUDY METHODS ..... 6

4.1 General Study Design and Plan ..... 6

4.2 Intervention Arms ..... 6

4.3 Study Population ..... 8

4.4 Randomization and Blinding ..... 8

4.5 Study Assessments ..... 8

4.6 Description of Variables ..... 9

5 SAMPLE SIZE ..... 12

5.1 Original Sample Size Determination ..... 12

5.2 Revised Sample Size Determination ..... 13

6 GENERAL ANALYSIS CONSIDERATIONS ..... 13

6.1 Timing of Analyses ..... 13

6.2 Analysis Populations ..... 13

6.3 Covariates and Subgroups ..... 13

6.4 Missing Data ..... 14

6.5 Summary of Study Data ..... 14

6.6 Subject Disposition ..... 14

6.7 Protocol Deviations ..... 14

6.8 Demographic and Baseline Variables ..... 14

6.9 Outcome Analyses ..... 15

6.10 Baseline Descriptive Analyses ..... 15

6.11 Primary Outcome Analysis ..... 15

6.12 Secondary Outcome Analyses ..... 16

6.13 Exploratory Outcome Analyses ..... 17

6.14 Interim Analyses ..... 17

6.15 Sub-Group Analyses ..... 17

6.16 Post-Hoc Analyses ..... 17

6.17 Safety Analyses ..... 17

7 REPORTING CONVENTIONS ..... 18

8 SUMMARY OF CHANGES TO THE SAP ..... 18

9 REFERENCES ..... 20

APPENDIX A: ELECTRONIC HEALTH RECORD OUTCOME VARIABLES ..... 21

APPENDIX B: MEDICATIONS PRESCRIBED FOR TREATMENT INITIATION FROM ELECTRONIC HEALTH RECORD ..... 22

## 1 Abbreviations and Definitions

**Table 1.** Abbreviations and Definitions

| | |
|---|---|
| AE | Adverse events |
| AMS | Addiction medicine specialist |
| ASSIST | Alcohol, Smoking, and Substance Involvement Screening test |
| BH | Baystate Health |
| BMC | Boston Medical Center |
| CC | Collaborative care |
| CCT | Collaborative care team |
| CL | Consultation liaison |
| CM | Care manager |
| CSMC | Cedars-Sinai Medical Center |
| ED | Emergency Department |
| EMR | Electronic medical record |
| FDA | United States Federal Drug Administration |
| GAIN | Global Appraisal of Individual Needs |
| GAD-7 | Generalized anxiety disorder-7 |
| IPW-MI | Inverse probability weighting and multiple imputation |
| MOUD | Medication(s) for opioid use disorder |
| NSDUH | National Survey on Drug Use and Health |
| OAMAT | Opinions about MAT |
| OUD | Opioid use disorder |
| PACIC | Patient assessment of chronic illness care |
| PCP | Primary care provider |
| PEG | Pain, Enjoyment of Life, and General Activity scale |
| PHQ-9 | Patient Health Questionnaire-9 |
| PROMIS | Patient-Reported Outcomes Measurement Information System |
| PTSD | Post-traumatic stress disorder |
| RCT | Randomized controlled trial |
| REDCap | Research Electronic Data Capture system |
| SAE | Severe adverse event |
| SAP | Statistical Analysis Plan |
| SAS | Statistical analysis system |
| SDCC | (UNM) Statistics and Data Coordinating Center |
| SRG | Survey Research Group |
| START | Substance Use Treatment and Recovery Team |
| UC | Usual Care |
| UNM | University of New Mexico |

# 2 Introduction

The purpose of this study is to evaluate whether an intervention by an interdisciplinary collaborative care team (Substance Use Treatment and Recovery Team (START) intervention) compared with usual care for hospitalized patients with opioid use disorder (OUD) can increase initiation of medication for opioid use disorder (MOUD) and improve linkage to OUD-focused follow-up care. If the aims of the research are achieved, we hope to improve MOUD initiation and linkage to follow-up care as well as clinical outcomes, and, ultimately, create a generalizable, sustainable model of care to increase OUD treatment delivery and decrease the downstream effects of untreated OUD. If effective, this translational model also can be used to increase uptake of evidence-based practices for other substance use and associated behavioral health disorders.

# 3 Study Objectives and Endpoints

## 3.1 Study Objectives

### 3.1.1 Primary Objectives

#### 3.1.1.1 Primary Objective 1: To test the effectiveness of the START intervention on MOUD initiation relative to usual care.

#### 3.1.1.2 Primary Objective 2: To test the effectiveness of the START intervention on linkage with post-discharge OUD treatment relative to usual care.

### 3.1.2 Secondary Objectives

#### 3.1.2.1 Secondary Objective 1: To test the effectiveness of the START intervention on addiction-focused discharge planning.

#### 3.1.2.2 Secondary Objective 2: To test the effectiveness of the START intervention on MOUD engagement relative to usual care.

#### 3.1.2.3 Secondary Objective 3: To test the effectiveness of the START intervention on linkage to medical care relative to usual care.

#### 3.1.2.4 Secondary Objective 4: To test the effectiveness of the START intervention on self-reported days of opioid use relative to usual care.

### 3.1.3 Exploratory Objectives

## 3.2 Endpoints

The objectives described above will be measured with the following endpoints. Intervention arms are described in Section 4.2.

### 3.2.1 Primary Endpoints

Primary Endpoint 1: Proportion of patients in each arm who initiate MOUD prior to discharge, defined as use of any FDA-approved pharmacotherapy for OUD, including buprenorphine, naltrexone and methadone.

3.2.2 **Primary Endpoint 2:** Proportion of patients in each arm who attend at least one OUD-related care visit within 30 days of hospital discharge.

### 3.2.3 Secondary Endpoints

3.2.4 **Secondary Endpoint 1:** Proportion of patients in each arm with an after-hospital care plan that specifies a date and time for a post-discharge addiction care appointment.

3.2.5 **Secondary Endpoint 2:** Proportion of patients in each arm who initiate MOUD or continue

MOUD treatment within 30 days following hospital discharge.

3.2.6 **Secondary Endpoint 3:** Proportion of patients in each arm who complete at least one visit to an outpatient medical provider within 30 days of hospital discharge.

3.2.7 **Secondary Endpoint 4:** Days of opioid use in the past 30 days.

3.2.8 **Exploratory Endpoints**

# 4 Study Methods

## 4.1 General Study Design and Plan

This is a multisite, pragmatic randomized controlled trial (RCT) assessing the effectiveness of the START intervention (an interdisciplinary collaborative care team) for hospitalized patients with OUD as compared to usual care. Participants are inpatients at three medical hospitals in California, Massachusetts, and New Mexico. They must be 18 years or older, are admitted for any reason, and screen positive for moderate to severe OUD using the ASSIST. They are identified through provider referral and through a daily report produced from electronic medical records (EMR) at each site, pre-screened for potential eligibility, and then approached for screening by research coordinators. If eligible and interested, the participant is consented, completes the baseline interview, and is randomized to either the START intervention or usual care. Between 30- and 60-days following discharge, the participant is contacted by phone to complete a follow-up interview. See Figure 1 for the study CONSORT diagram. Primary and secondary outcomes measures are obtained from the EMR and from follow-up interviews.

Each site has research coordinators who screen participants for eligibility, perform the consent and baseline interview, and randomize them to one of the two intervention arms. The RAND Survey Research Group (SRG) performs the 1-month phone follow-up interviews. All eligibility, baseline, and 1-month follow-up interview data are entered by the described site staff into the UNM instance of REDCap. The UNM Statistics and Data Coordinating Center (SDCC) is responsible for developing the REDCap databases, providing technical support to staff, and managing the data to ensure the quality of the data, and creating analytical data sets. Each site is responsible for obtaining EMR-based outcomes data from their EMR and sending it via approved, secure methods to the UNM SDCC. Enrollment was originally expected to last approximately 11 months, but has been extended to approximately 32 months due to the impact of the COVID-19 global pandemic and slower than expected enrollment.

## 4.2 Intervention Arms

4.2.1 **The START intervention arm** consists of an addiction medicine specialist (AMS) and a care manager (CM) who use evidence-based tools such as motivational interviewing and addiction-focused discharge planning to decrease barriers to MOUD and engage patients with post-discharge OUD care.

4.2.2 **UC** consists of each hospital’s current practices for managing patients identified with OUD along with each patient enrolled in the study receiving MOUD education and referral information. We use UC as the comparator because there are no other evidence-based interventions for achieving our proposed outcomes. At CSMC, patients randomized to the UC study condition may receive a referral to the existing consultation liaison (CL) psychiatry service if the patient’s medical team determines the need for a consult, or they will be treated and provided discharge planning directly by the medical team. At UNM and BMC hospitals, patients randomized to the UC study condition can be treated directly with MOUD and provided discharge planning by the medical team. At BMC Hospital, the referring physician has the option of contacting the standard psychiatric CL or addiction consult service for patients in the UC study condition, which will not include an AMS or CM. If the START AMS or CM at any hospital is approached by a member of the medical team for consultation on an OUD patient, they will refer them to the California Bridge

Figure 1. START CONSORT Diagram

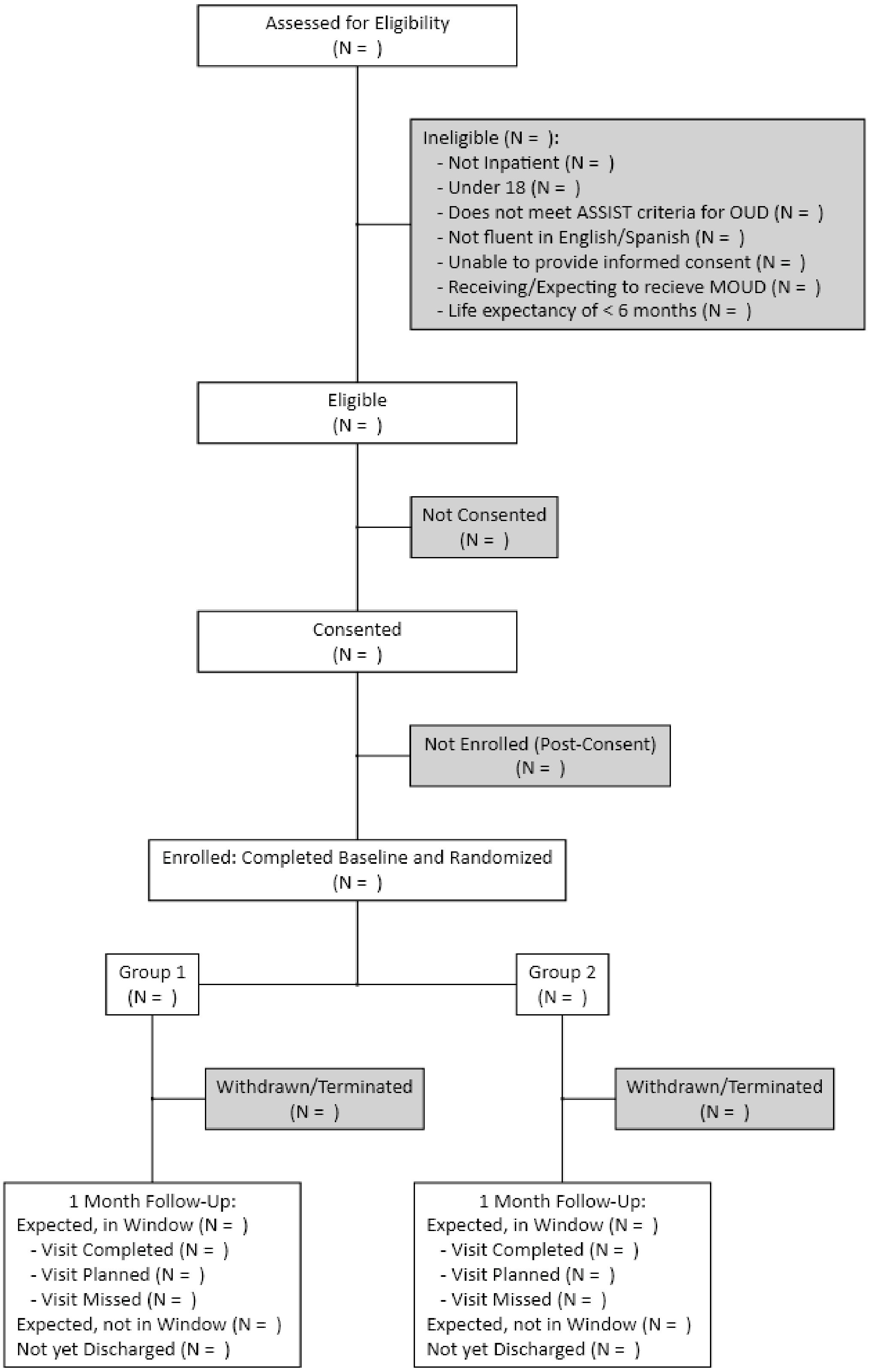

Program Tools and Resources website None of the hospitals in this study currently employs a collaborative care team that consists of an AMS – CM team that uses a set of principles based on collaborative care along with evidence-based tools to support the medical team in intervening with patients with OUD and delivering for OUD treatment in the hospital and after discharge.

## 4.3 Study Population

### 4.3.1 Inclusion Criteria

In order to be eligible to participate in this study, an individual must meet all of the following criteria:

- Admitted to an inpatient bed at Cedars-Sinai Medical Center (CSMC), University of New Mexico Hospital (UNM), or Baystate Health (BH)
- Age 18 and older
- Have a probable OUD diagnosis, defined by scores of >3 on the opioid section of the Alcohol, Smoking, and Substance Involvement Screening test (ASSIST)
- Speaks English or Spanish as primary language
- Able to provide informed consent

### 4.3.2 Exclusion Criteria

An individual who meets any of the following criteria is excluded from participation in this study:

- Currently receiving FDA-approved medication treatment for an opioid use disorder*
- <6 months life expectancy

*"Currently receiving medication" is defined as medications received by patient while in the hospital, as indicated by the EMR, medical team, or by patient self-report on the eligibility screener of taking the medication since their admission.

## 4.4 Randomization and Blinding

### 4.4.1 Randomization

Participants are randomized to either the START intervention or usual care. A stratified, block randomization design is used to stratify by site and prior MOUD exposure (yes/no), with randomly permuted block sizes of 2, 4, and 8. The intervention arm allocation was programmed in R 4.1 using the package blockrand[1] and outputted as a .CSV file for upload into REDCap's randomization module. Two allocation schedules have been generated: one for testing and a separate one for production. Research staff access their site-specific randomization module and enter which MOUD stratum the patient is in and the intervention arm assignment is displayed. Enrollment will be continuous with the goal of reaching the target sample size. Some sites may enroll more or less than the target for each arm. Prior MOUD exposure is defined as ever taken medications to treat an opioid use problem (specific medications are buprenorphine, buprenorphine/naloxone, methadone, naltrexone).

### 4.4.2 Blinding

The baseline interview occurs prior to randomization so research staff conducting baseline interviews are blind to study condition. Follow-up interviews are performed by RAND SRG research staff who initially will be blinded to study condition at the beginning of the interview, although blinding is broken during the course of the follow-up interview due to branching questions for START participants only.

## 4.5 Study Assessments

### 4.5.1 Schedule of Study Assessments

Table 2 displays the scheduled study assessments.

### 4.5.2 Visit Windows

Participants are randomized, and hence considered enrolled, following completion of consent

and the baseline interview. The 1-month follow-up assessment occurs within a 60-day window starting at 30 days post-discharge from hospital (i.e., between Day 30 to Day 90, with Baseline being Day 0).

Study staff will not attempt to contact a participant after their visit window has closed. If the 1-month follow-up visit is not completed by Day 90, they will be considered lost to follow-up.

**Table 2. RCT Schedule of Assessments**

| Instrument/Questionnaire | Screening | Consent and Baseline visit | 1-month post-discharge |
|---|---|---|---|
| ASSIST | X | | |
| Current MOUD Utilization | X | | |
| Informed Consent | X | | |
| Sociodemographic Data | X | X | |
| Pain Intensity and Frequency (PEG) | | X | X |
| Depression (PHQ-9) | | X | X |
| Anxiety (GAD-7) | | X | X |
| 30-Day Opioid (and other substance) Use (adapted from NSDUH) | | X | X |
| SUD Treatment Utilization (adapted from NSDUH) | | X | X |
| SUD Healthcare and Mental Health Utilization (adapted from GAIN) | | X | X |
| Severity of Substance Use (PROMIS) | | X | X |
| Overdoses | | X | X |
| Experience of Stigma | | X | |
| Social Support (MSPSS) | | X | |
| Opinions about MAT(MOUD) | | X | |
| Significant Other with OUD | | X | |
| Criminal Justice Involvement | | X | |
| MOUD Utilization | | | X |
| Patient Experience of Chronic Illness Care (PACIC) | | | X |
| Therapeutic Alliance (CAHPS) [a] | | | X |
| Satisfaction with START [a] | | | X |

[a] Only for participants randomized to the START intervention arm of the study

## 4.6 Description of Variables

### 4.6.1 Description of Outcome Variables

Table 3 describes the outcome variables and data sources.

**Table 3. Outcome variables and sources**

| Outcome | Endpoint | Data Source |
|---|---|---|
| **Primary Outcomes** | | |
| In-hospital initiation of MOUD therapy | Proportion of patients in each arm who initiate MOUD prior to discharge, defined as use* of any FDA-approved pharmacotherapy for OUD, including buprenorphine, naltrexone and methadone. *Use means the MOUD was noted as ordered or administered in the EMR. | EMR (See Appendices A and B) |

| | | |
|---|---|---|
| Linkage to follow-up OUD care | Proportion of patients in each arm who attend at least one OUD-related care visit within 30 days of hospital discharge | 1-month interview |
| **Secondary Outcomes** | | |
| OUD-specific discharge plan | Proportion of patients in each arm with an after-hospital care plan that specifies a date and time for a post-discharge addiction care appointment | EMR chart review for non-START patients and the registry for START patients (See Appendices A and B) |
| Any post-discharge MOUD utilization | Proportion of patients in each arm who initiate MOUD or continue MOUD treatment within 30 days following hospital discharge | 1-month interview |
| Post-discharge outpatient medical care | Proportion of patients in each arm who complete at least one visit to an outpatient medical provider within 30 days of hospital discharge. Visit must be specifically related to opioid use and may include an emergency department visit. | 1-month interview |
| Past 30-day number of days with any opioid use (each substance separately and substance days (sum of substances) | Mean (or median, depending on distribution) days of use in the past 30 days after hospital discharge– Adapted National Survey of Drug Use and Health (NSDUH).[2] "Use-days" range from 0 to 120 days with up to 30 days of use reportable for each of four opioid categories: pain medications excluding fentanyl, fentanyl, heroin/opium alone, heroin/opium mixed with another drug | Baseline interview<br>1-month interview |

The following EMR data elements will be obtained to derive the outcome variables and/or to describe the sample:

- Hospital encounter data: Type; Dates; Disposition; Attending provider; PCP; Psychiatry consult;
- Reason for admission
- Diagnoses
- Inpatient medication (listed by generic names): Buprenorphine; Buprenorphine/Naloxone; Methadone; Naltrexone; Naloxone.
- Hospital utilization metrics: Length of stay; Inpatient admissions in prior 12 months; ED admissions in prior 12 months; Number of 30-day readmissions
- Insurance type
- Presence of an OUD-specific discharge plan in the record

### 4.6.2 Description of Other Measures

#### 4.6.2.1 Table 4 lists other outcomes, covariates, potential mediators and moderators.

**Table 4. Description of other outcomes, covariates, potential mediators and moderators**

| Variable | Measure | Data Source | Response Values/Scales |
|---|---|---|---|
| **Sociodemographics** | ***(Covariate; Potential Moderator)*** | | |
| • Age | | Eligibility Screener | Continuous |
| • Sex (Assigned at Birth) | | Eligibility Screener | Binary |
| • Gender Identity | | Eligibility Screener | Categorical (1-4) |
| • Hispanic | | Eligibility Screener | Binary |
| • Race | | Eligibility Screener | Categorical (1-5) |

**Table 4. Description of other outcomes, covariates, potential mediators and moderators**

| Variable | Measure | Data Source | Response Values/Scales |
|---|---|---|---|
| • Current homeless status | | Eligibility Screener | Binary |
| • Marital status | | Baseline Interview | Categorical (1-6) |
| • Income | | Baseline Interview | Continuous |
| • Education | | Baseline Interview | Categorical (1-20) |
| • Insurance type | Payer name | EMR | Text (code to numeric) |
| **Mental Health Symptoms** | ***(Covariate; Potential Moderator/ Mediator)*** | | |
| • Depression (9 items) | PHQ-9[3,4] | Baseline Interview 1-month Follow-up | Likert-type (1-4) |
| • Anxiety (7 items) | GAD-7[5-7] | Baseline Interview 1-month Follow-up | Likert-type (1-4) |
| **Social Support Scale** | ***(Covariate; Potential Moderator)*** | | |
| • Social support: Family, Friends, Significant Other (6 items; 2 each scale) | Modified Multidimensional Scale of Perceived Social Support[8] | Baseline Interview 1-month Follow-up | Likert-type (1-7) |
| **Medical Symptoms/Treatment** | ***(Covariates; potential mediator/moderator)*** | | |
| • Overdoses (lifetime, past 3 mos) | N/A | Baseline Interview 1-month Follow-up | Continuous |
| • Primary and secondary diagnosis (inpatient stay) | Medical or mental health conditions as determined by the inpatient physician | EMR | Text (ICD codes) |
| • Pain intensity and duration | PEG[9] | Baseline Interview 1-month Follow-up | 0-10 scale |
| • Length of hospital stay | Days in hospital | EMR | Continuous |
| **Substance Use Treatment History** | ***(Covariates; potential moderator)*** | | |
| • Ever used an MOUD<br>• Times started an MOUD | N/A | Eligibility Screener | • Binary<br>• Continuous |
| • Type of MOUD medication | N/A | Eligibility Screener | Categorical (1-4) |
| • Treatment other than MOUD<br>• Times had treatment other than MOUD | N/A | Eligibility Screener | • Binary<br>• Continuous |

**Table 4. Description of other outcomes, covariates, potential mediators and moderators**

| Variable | Measure | Data Source | Response Values/Scales |
|---|---|---|---|
| **Recent Substance Use Treatment Utilization; Opinions; Consequences Stigma** | ***(Outcomes*; Covariates)*** | | |
| SUD Treatment Utilization* (5 items)<br>• Past 90 days baseline<br>• Past 30 days from discharge follow-up*<br>***Linkage outcome** | Adapted from National Survey on Drug Use and Health (NSDUH)[2] | Baseline Interview<br>1-month Follow-up | Binary |
| Healthcare Utilization (ER, Inpatient, Outpatient) Related to SUD (5 items)<br>• Past 90 days baseline<br>• Past 30 days follow-up | Adapted from Global Appraisal of Individual Needs (GAIN)[10] | Baseline Interview<br>1-month Follow-up | Continuous |
| • Familiar with MOUD<br>• Opinions about MOUD (3 items) | Opinions about MAT (OAMAT)[11] | Baseline Interview | Likert-type (1-5) |
| Severity of Substance Use (7-items) | PROMIS | Baseline Interview<br>1-month follow-up | Likert-type (1-5) |
| Patient Experience of Stigma (5 items) | Adapted from Grosso et al. 2019.[12] | Baseline Interview | Binary |
| Patient Experience of Chronic Illness Care (11 items) | Patient Assessment of Chronic Illness Care (PACIC)[13] | 1-month Follow-up | Binary |
| Criminal Justice Involvement<br>• Ever arrested<br>• Times arrested past 90 days | Locally developed | Baseline Interview | • Binary<br>• Continuous |

# 5 Sample Size

## 5.1 Original Sample Size Determination

In-patient MOUD initiation: A sample size of n=432 (allowing for 20% attrition) and adjusted type I error rate of 2.5% provides 84% power to detect an OR=2.3 comparing the in-patient MOUD initiation rates in the CCT and UC arms, stratified on prior MOUD use. Based on literature, 14% of UC patients who are MOUD-naïve initiate MOUD in hospital.[14] Assuming the average of MOUD-naïve and MOUD-experienced in-patient MOUD initiation rates is 20%, we have an adequate sample size and power to detect this increase of in-patient MOUD initiation in the CCT arm (37%) compared to UC.[14-16]

Linkage to OUD Care: We base the sample size estimate on the linkage to care measure since the probabilities of successful linkage are lower than for in-patient MOUD initiation. Linkage to care rates reported in the literature range between 10%-17% in usual care settings. To err on the side of caution, we estimate linkage to care in UC for MOUD-naïve and MOUD-experienced to be 5% and 10%,[14-17] respectively, yielding an average of 7.5%. We hypothesize that at least 20% of patients randomized to the START arm will link to OUD care (attend at least one OUD-related visit) within 30 days following discharge. Assuming a Bonferroni-corrected, two-sided type I error rate of 2.5% to adjust for two primary endpoints, we will enroll a minimum of 432 patients (216 in each intervention arm) to have 80% power to detect this difference. This estimate includes an adjustment for up to 20% attrition. This effect size corresponds to a clinically meaningful odds ratio of 3.0. Prior studies in different settings have found larger effects,[16-18] supporting our ability to conduct this test.

Sample size calculations for the primary endpoints were performed in PASS 14 using stratified Mantel-Haenszel tests for two proportions between two groups,[19] with strata defined as 50% MOUD-naïve and 50% MOUD-experienced.[16-18,20,21] Due to the short 1-month duration of participation, subject withdrawal from the study is not anticipated to be significant.

### 5.2 Revised Sample Size Determination

Our original sample size estimates were based on an assumption for the stratification variable, prior MOUD exposure, that equal proportions would be observed (50% with prior MOUD exposure and 50% without). As of February 2023, we are presently observing 76% with prior MOUD exposure and 24% without. Based on this new information about our randomization strata, we recalculated the sample size needed to analyze our primary outcomes MOUD, linkage to OUD treatment). We determined that the sample sizes needed to analyze both primary outcome effect sizes originally proposed with 80% power and type I error = 2.5% (Bonferroni-corrected for two primary outcomes) are:

- MOUD initiation: n = 288. Given that this outcome is observed on every enrolled participant, there is no need to inflate the target sample size for attrition.
- Linkage to care: n = 299. With an observed attrition of 30%, we require enrolling 426 participants, still requiring enrollment through the end of the study period.

## 6 General Analysis Considerations

### 6.1 Timing of Analyses

The study databases will be locked to data entry 90 days following the last enrolled participant's discharge from hospital. This allows for one month following study completion of the last participant for completion of standard quality control queries. Any additional queries identified following the data lock will be addressed and any final query resolutions to the data will be hard-coded into the data management programs and documented in the code.

Analyses described in this SAP will commence once the locked data set is created and will be completed during the final year of the study.

### 6.2 Analysis Populations

#### 6.2.1 Intention to Treat (ITT) Population

All subjects who consented, enrolled, and were randomized into either arm. Participants who complete the informed consent or part of the baseline interview but are not randomized will be excluded from analysis.

#### 6.2.2 Per Protocol (PP) Population

All subjects who consented, enrolled, and were randomized into either arm and who completed the 1-month follow-up interview.

### 6.3 Covariates and Subgroups

#### 6.3.1 Covariates

Potential covariates are listed in Table 4.

#### 6.3.1 Subgroups

We will conduct exploratory analyses to see if patient sex or gender, or race/ethnicity has an effect on primary outcomes or retention. Adjusted odds ratios and their 95% confidence intervals will be calculated from interaction effects between treatment group and sex or gender from the specified linear models for the primary and secondary outcome measures.

#### 6.3.2 Multi-Center Studies

This is a multisite study consisting of three sites. The data will be pooled across sites to assess the primary

and secondary outcomes. Randomization is stratified by site and thus site will be included in all analyses as an influential covariate and interaction effects between site and treatment arm will be assessed.

### 6.4 Missing Data

Study endpoints are cross-sectional in time. Every effort will be made to obtain all necessary outcome and covariate data. We will use inverse probability weighting and multiple imputation (IPW-MI) to adjust for missing covariate data.[22] Specifically, we will examine whether observable baseline characteristics differ by attrition status, and if so we will adjust our comparisons using weights. MI will be used to impute intermittently missing data for study completers. We will not impute outcomes, but only covariates.

### 6.5 Summary of Study Data

Data will be summarized with descriptive statistics including frequencies, means, standard deviation, medians, quartiles, minima, and maxima for continuous data, as appropriate. Frequencies and percentages will be used to summarize categorical data. Categorization of continuous variables for frequency tables will be predetermined by logical cutoffs, e.g., 5-year age groups, or tertiles and quartiles.

### 6.6 Subject Disposition

Study status of subjects will be summarized with descriptive statistics, as described above, throughout the study. For the one-month follow-up visit, we will summarize the number and proportion of subjects whose interview occurred, how many dropped out, were terminated from the study and for what reasons, and how many were lost-to-follow-up. Early termination reasons include:

1. Found to be ineligible after randomization
2. Participant withdrew consent
3. Participant death
4. Other

### 6.7 Protocol Deviations

Reported protocol deviations are missed visits and visits that occurred beyond the defined window period (+ 60 days from target date). We will report the total number of deviations and the frequency and percentage of each reason. Individual listings will also be produced.

The following reasons are being collected:
1. Incomplete visit
2. Missed visit
3. Visit out of window
4. Informed consent deviation
5. Missing or incorrect documentation
6. Eligibility deviation
7. HIPAA violation
8. Single subject protocol exception
9. Other (describe)

### 6.8 Demographic and Baseline Variables

Intervention arms will be compared on baseline characteristics (e.g., demographics, mental health characteristics) using descriptive statistics. Planned comparisons are below.

- Site
- Age
- Sex at birth
- Gender identity
- Ethnicity (Hispanic/Latino)
- Race
    - American Indian/Alaska Native

- Asian/Pacific Islander
- Black/African American
  White
- Multiple Races
- Other Race

- Insurance Status
- Income
- Housing Status
- Depression (PHQ-9)
- Anxiety (GAD-7)
- Severity of Substance Use (PROMIS)
- Sever to moderate substance use of other substances (ASSIST)
- Substance use treatment prior to hospitalization (eligibility screener)
- Pain severity (PEG) and duration (survey question)

## 6.9 Outcome Analyses

Analyses comparing demographic and clinical characteristics of the treatment arms will be assessed as follows:
Continuous variables:
- ANOVA or Mann Whitney tests will be used to compare across 3 or more groups
- *t* tests or Wilcoxon rank sum tests for comparing 2 groups

Categorical variables:
- Tests of proportions for comparing 2 groups
- $\chi 2$ tests, or Fisher exact tests, as appropriate for the data type for 2 or more categories
- Logistic regression for interaction effects between group and given variable.

Such analyses will be used to assess baseline homogeneity of the treatment groups as well as to help us identify potential covariates to be included in linear models for assessment of outcome measures; however, clinically relevant covariates will also be included regardless of the outcomes of these analyses. All analyses will be performed in SAS 9.4[23] or higher, R 4.2[24] or higher, and/or Stata 17[25] or higher.

## 6.10 Baseline Descriptive Analyses

Intervention arms will be compared on baseline characteristics (e.g., demographics, mental health characteristics) using descriptive statistics. Planned comparisons are for the variables described in section 6.8.

## 6.11 Primary Outcome Analysis

### 6.11.1 Primary Hypotheses

#### 6.11.1.1 Primary Hypothesis 1

We hypothesize that, compared to patients who receive usual care, patients in the START condition will be more likely to initiate MOUD while hospitalized. Alternatively, our null hypothesis is that there will be no difference in likelihood of in-hospital MOUD initiation between patients in the START and UC conditions.

#### 6.11.1.2 Primary Hypothesis 2

We hypothesize that, compared to patients who receive usual care, patients in the START condition will be more likely to receive linkage to post-discharge MOUD care (i.e., attend at least one OUD-related care visit within 30 days of hospital discharge). Alternatively, our null hypothesis is that there will be no difference in linkage to post-discharge MOUD care for patients in the START condition compared to those in UC condition.

### 6.11.2 Primary Analysis

#### 6.11.2.1 Original Primary Analytical Plan

Unadjusted point estimates and confidence intervals for proportions and means will be reported by arm and

by prior MOUD use for endpoints. Primary endpoints will be compared between arms by fitting a multivariable logistic regression model to each that includes as independent variables: intervention arm, prior MOUD exposure and site, as well as relevant baseline characteristics as covariates, including age, income or insurance status (as a marker for income), race, and ethnicity. Additional covariates that may be included are substance use severity, homelessness and length of index hospitalization, as well as any other variables also thought to be associated with outcomes that demonstrated imbalance between treatment arms.[18] Site will be included as a fixed effect to reflect the study design and to control for potential variability in CCT implementation. Odds ratios and their Bonferroni-adjusted 97.5% Wald confidence intervals will be reported for the two primary outcomes.

6.11.2.2 **Revised Primary Analytical Plan**

Upon initial descriptive analyses of the primary outcomes, we discovered that the proportion of patients in each treatment arms who initiated MOUD in the hospital and who linked to care post-discharge was much higher than hypothesized. Odds ratios (ORs) are frequently used to report effect sizes for dichotomous outcomes; however, when the outcome rates are not rare, ORs tend to overestimate the effect size which could lead to overinterpretation of the results. We determined that risk ratios (RRs) are a better estimate of the true effect and are less likely to lead to overinterpretation of effects. Therefore, multivariable Poisson regression models were fitted to each of the primary endpoints to compare treatment arms. These models will include the covariates described for the original analysis plan (age, insurance status, race, and ethnicity) and the independent variables previously stated (intervention arm, prior MOUD exposure, and site). Additional covariates as described previously may be considered. RRs and their 97.5% Wald confidence intervals will be reported for the two primary outcomes.

## 6.12 Secondary Outcome Analyses

### 6.12.1 Secondary Hypotheses

6.12.1.1 **Secondary Hypothesis 1**

We hypothesize that, compared to patients who receive usual care, patients in the START condition will be more likely to receive addiction-focused discharge planning. Alternatively, our null hypothesis is that there will be no difference in likelihood of receiving addiction-focused discharge planning between patients in the START and UC conditions.

6.12.1.2 **Secondary Hypothesis 2**

We hypothesize that, compared to patients who receive usual care, patients in the START condition will be more likely to receive MOUD treatment after discharge (i.e., initiate MOUD or continue MOUD treatment within 30 days following hospital discharge). Alternatively, our null hypothesis is that there will be no difference in likelihood of receiving MOUD treatment between patients in the START and UC conditions.

6.12.1.3 **Secondary Hypothesis 3**

We hypothesize that, compared to patients who receive usual care, patients in the START condition will be more likely to receive linkage to post-discharge medical care (i.e., complete at least one visit to an outpatient medical provider within 30 days of hospital discharge). Alternatively, our null hypothesis is that there will be no difference in linkage to post-discharge medical care for patients in the START condition compared to those in UC condition.

6.12.1.4 **Secondary Hypothesis 4**

We hypothesize that, compared to patients who receive usual care, patients in the START condition will be more likely to significantly reduce opioid use after discharge (i.e., days of opioid use in the 30 days between discharge and follow-up). Alternatively, our null hypothesis is that there will be no difference in likelihood of reducing opioid use between patients in the START and UC conditions.

### 6.12.2 Secondary Analyses

6.12.2.1 **Original Secondary Analytical Plan**

Similar analyses as described for the primary endpoints will be performed for these secondary proportions

outcomes, but instead reporting 95% confidence intervals. For Secondary Endpoint 4, a general linearized model to number of days of opioid use will be fitted along with the covariates described for the logistic regression models. An appropriate link function will be identified and used based on the distribution of the outcome data.

#### 6.12.2.2 **Revised Secondary Analytical Plan**

For the same reasons described in Section 6.11.2.2, we determined that multivariable Poisson regression models were more appropriate for the data than logistic regression models and, therefore, were applied to Secondary Endpoints 1-3 with independent variables as previously described. For these, we will report RRs and their 95% CIs. For Secondary Endpoint 4, we fit a multivariable negative binomial regression model with log link function to opioid use-days with the covariates previously described with the addition of baseline opioid use-days. Contrasts will be calculated to estimate the incident rate ratio (IRR) to compare treatment arms, along with its 95% CI.

## 6.13 Exploratory Outcome Analyses

To explore possible mechanisms of how START works, we may conduct the following exploratory analyses: (1) Assess the mediating effect of inpatient MOUD initiation on use of MOUD and linkage with OUD treatment post-discharge; (2) Assess the mediating effect of completion of an OUD-specific discharge plan on linkage with OUD treatment 30-days post-discharge; (3) Assess the moderating effects of patient characteristics (e.g., gender, race, ethnicity, insurance status) on post-discharge linkage. Analysis plans for these exploratory assessments will be written separately.

## 6.14 Interim Analyses

Given the additional information about the reduced sample size (from our original, pre-study calculations) needed for the medication initiation outcome, and the desire to minimize participant burden, an interim analysis will be conducted on the second primary outcome (linkage) when we reach n = 288 enrolled (the sample size required to estimate our primary outcome measure of MOUD initiation). This will provide approximately n = 288×0.70 = 202 participants for this analysis, or ~68% (202/299) of our final analytic sample size for this outcome. We will utilize an alpha-spending method[26,27] to ensure that, should we continue the study until full enrollment, we control the family-wise type I error rate to be 2.5% for each outcome. Using a two-sided test, this approach will allow us to assess superiority of the intervention over the control or the control over the intervention. If we reject the null hypothesis at the interim analysis, we will discontinue the study. Should we fail to reject the null hypothesis at the interim analysis, we will continue to full enrollment for final analysis. The interim analysis two-sided type I error level was calculated in the R package “rpact”, yielding a comparison of this outcome’s p-value to $\alpha$=0.0062.

## 6.15 Sub-Group Analyses

We will conduct exploratory analyses to see if patient sex or gender, or race/ethnicity has an effect on primary outcomes or retention. Adjusted odds ratios and their 95% confidence intervals will be calculated from interaction effects between treatment group and sex or gender from the specified linear models for the primary and secondary outcome measures.

## 6.16 Post-Hoc Analyses

The START leadership team (Ober, Danovitch, Page, Friedmann, and Murray-Krezan) will encourage collaboration across all sites and provide guidance to promote and support scientific research dissemination. Research “ideas” for abstracts, manuscripts and presentation will be generated on “Concept Sheets” and submitted for review to the START Leadership group. Concept sheets will each have detailed analysis plans for the proposed study question/s. All papers will include a biostatistician in the collaborative/authorship group. In general, we expect both descriptive and comparative analyses will be conducted on cross-sectional and longitudinal data collected in the START cohort study.

## 6.17 Safety Analyses

All adverse events (AEs) will be categorized and graded for severity as described above. (S)AEs will be

summarized via the same methods described in section 8.0, by site and overall. SAEs will be individually listed for DSMB review and will also be categorized and summarized similarly to AEs. We report AEs and SAEs by number of events (# AEs may be > N), as well as by the subject's most severe AE and its severity (#AEs ≤ N). These analyses will be performed on the ITT defined in Section 6.2.1.

#### 6.17.1 Adverse Events

The START protocol defines an adverse event (AE) as any unfavorable and unintended symptom or disease that an investigator or study staff learns about which occurs during a patient's enrollment in the study, if it is considered by the site study team to be possibly related to a study treatment or procedure ("possibly related" means there is a reasonable possibility that AE may have been caused by research procedures).

#### 6.17.2 Serious Adverse Events

##### 6.17.2.1 Definition of SAE

The START protocol defines a serious adverse event (SAE) as an AE that an investigator or study staff learns about that is fatal, life-threatening, prolongs initial hospitalization, requires inpatient rehospitalization, or is medically significant and which the investigators and/or clinicians regard as serious based on appropriate medical judgment. With the exception of fatalities, other SAEs documented this study are considered those is possibly related to the study; other events occurring during the normal of the hospital stay will be numerous and thus not documented unless possibly related to the study.

#### 6.17.3 Relationship to Study Intervention and Severity of (S)AEs

All (S)AEs will be rated as Mild, Moderate, or Severe and will be used as a factor in determining expectedness of an event. All (serious) adverse events will have their relationships to the study intervention assessed and rated as either Definitely Related, Probably Related, Unlikely to be Related, or Not related.

#### 6.17.4 Pregnancies

Pregnant people are not excluded from participation in this study. We do not follow pregnancy outcomes given the short duration of the study period. Nevertheless, any adverse events for pregnant participants will be documented in accordance with our AE/SAE reporting protocol.

## 7 Reporting Conventions

The following reporting conventions will be applied to all reports:

- P-values ≥0.001 will be reported to 3 decimal places; p-values less than 0.001 will be reported as "p<0.001".
- The mean, standard deviation, and any other statistics other than quantiles, will be reported to one decimal place greater than the original data. Quantiles, such as median, or minimum and maximum will use the same number of decimal places as the original data.
- Estimated parameters, not on the same scale as raw observations (e.g., regression coefficients) will be reported to 3 significant figures.

## 8 Summary of Changes to the SAP

- The SAP was updated in April 2023 to revise the sample sizes needed for the primary outcomes given observed rates of the prior exposure to MOUD stratification variable. Additionally, we added a proposed an interim analysis to determine whether would be adequately powered to detect hypothesized primary effects with a smaller sample size given updated information about prior MOUD exposure and the attrition rate.

- The SAP was updated in March 2024 to describe the revised regression modeling for the Primary and Secondary Endpoints.

- The SAP was updated in December 2024 to clarify definitions of Secondary Endpoints 3 and 4.

## 9 References


1. Snow G. *Randomization for Block Random Clinical Trials*. 2020. Accessed June 30, 2023. https://cran.r-project.org/web/packages/blockrand/blockrand.pdf
2. Center for Behavioral Health Statistics and Quality. *2019 National Survey on Drug Use and Health (NSDUH): CAI Specifications for Programming (English Version).* 2018.
3. Gelaye B, Tadesse MG, Williams MA, Fann JR, Vander Stoep A, Andrew Zhou XH. Assessing validity of a depression screening instrument in the absence of a gold standard. *Ann Epidemiol*. Jul 2014;24(7):527-31. doi:10.1016/j.annepidem.2014.04.009
4. Kroenke K, Spitzer RL, Williams J. The PHQ-9: Validity of a brief depression severity measure. *J Gen Intern Med*. Sep 2001;16(9):606-13.
5. Löwe B, Decker O, Müller S, et al. Validation and standardization of the Generalized Anxiety Disorder Screener (GAD-7) in the general population. *Med Care*. 2008;46(3):266-74.
6. Ruiz MA, Zamorano E, García-Campayo J, Pardo A, Freire O, Rejas J. Validity of the GAD-7 scale as an outcome measure of disability in patients with generalized anxiety disorders in primary care. *J Affect Disord*. 2011;128(3):277-86.
7. Spitzer RL, Kroenke K, Williams JB, Löwe B. A brief measure for assessing generalized anxiety disorder: The GAD-7. *Arch Intern Med*. 2006;166(10):1092-7.
8. Zimet GD, Powell SS, Farley GK, Werkman S, Berkoff KA. Psychometric characteristics of the Multidimensional Scale of Perceived Social Support. *J Pers Assess*. Winter 1990;55(3-4):610-7. doi:10.1080/00223891.1990.9674095
9. Krebs EE, Lorenz KA, Bair MJ, et al. Development and initial validation of the PEG, a three-item scale assessing pain intensity and interference. *J Gen Intern Med*. Jun 2009;24(6):733-8. doi:10.1007/s11606-009-0981-1
10. M. D. *Global Appraisal of Individual Needs (GAIN): Administration Guide for the GAIN and Related Measures*. 2003. Accessed June 30, 2023. https://citeseerx.ist.psu.edu/document?repid=rep1&type=pdf&doi=b1f82c4e3f2db4218ff2b6bbbb9404ca1bc98e9e
11. Friedmann PD, Wilson D, Knudsen HK, et al. Effect of an organizational linkage intervention on staff perceptions of medication-assisted treatment and referral intentions in community corrections. *J Subst Abuse Treat*. Mar 2015;50:50-8. doi:10.1016/j.jsat.2014.10.001
12. Grosso AL, Ketende SC, Stahlman S, et al. Development and reliability of metrics to characterize types and sources of stigma among men who have sex with men and female sex workers in Togo and Burkina Faso. *BMC Infect Dis*. 2019/03/05 2019;19(1):208. doi:10.1186/s12879-019-3693-0
13. Glasgow RE, Wagner EH, Schaefer J, Mahoney LD, Reid RJ, Greene SM. Development and validation of the Patient Assessment of Chronic Illness Care (PACIC). *Med Care*. May 2005;43(5):436-44.
14. Nordeck CD, Welsh C, Schwartz RP, et al. Rehospitalization and substance use disorder (SUD) treatment entry among patients seen by a hospital SUD consultation-liaison service. *Drug and alcohol dependence*. May 1 2018;186:23-28. doi:10.1016/j.drugalcdep.2017.12.043
15. Trowbridge P, Weinstein ZM, Kerensky T, et al. Addiction consultation services - Linking hospitalized patients to outpatient addiction treatment. *Journal of substance abuse treatment*. Aug 2017;79:1-5. doi:10.1016/j.jsat.2017.05.007
16. Liebschutz JM, Crooks D, Herman D, et al. Buprenorphine treatment for hospitalized, opioid-dependent patients: a randomized clinical trial. *JAMA internal medicine*. Aug 2014;174(8):1369-76. doi:10.1001/jamainternmed.2014.2556
17. Cushman PA, Liebschutz JM, Anderson BJ, Moreau MR, Stein MD. Buprenorphine initiation and linkage to outpatient buprenorphine do not reduce frequency of injection opiate use following hospitalization. *Journal of substance abuse treatment*. Sep 2016;68:68-73. doi:10.1016/j.jsat.2016.06.003
18. Lee CS, Liebschutz JM, Anderson BJ, Stein MD. Hospitalized opioid-dependent patients: Exploring predictors of buprenorphine treatment entry and retention after discharge. *The American journal on addictions*. Oct 2017;26(7):667-672. doi:10.1111/ajad.12533
19. Champely S. Power analysis functions along the lines of Cohen (1988). The R Project. Accessed July 9, 2018, https://CRAN.R-project.org/package=pwr
20. NCSS Statistical Software. PASS 2023: Power analysis and sample size software. NCSS, LLC. https://www.ncss.com/software/pass/
21. The R Project. *The R Project for statistical computing*. 2017. Accessed July 9, 2018. https://www.R-

project.org/
22. Seaman SR, White IR, Copas AJ, Li L. Combining multiple imputation and inverse-probability weighting. *Biometrics*. Mar 2012;68(1):129-37. doi:10.1111/j.1541-0420.2011.01666.x

23. *SAS 9.4* SAS Institute Inc.; 2012. www.sas.com
24. *R version 4.2.0 (2022)*. The R Foundation for Statistical Computing; 2022. www.r-project.org
25. *Stata17.0*. Version Release 17. StataCorp; 2022. Accessed 2022. www.stata.com
26. DeMets DL, Lan K. K. . Interim analysis: the alpha spending function approach. *Stat Med*. July 15-30 1994;13(13-14):1341-1356; discussion 1353-6. doi:10.1002/sim.4780131308
27. Lan KK, DeMets DL. Further comments on the alpha-spending function. *Stat Biosci*. May 2009;1:95-111.

## APPENDIX A: Electronic Health Record Outcome Variables

| Description | Expected data type | Time Frame | Notes |
|---|---|---|---|
| Visit type | Inpatient or Observation | Visit FIN provided | |
| Visit admit date | Date | Visit FIN provided | |
| Visit discharge date | Date | Visit FIN provided | |
| Disposition | Categorical | Visit FIN provided | |
| Reason for admission | Free response | Visit FIN provided | |
| In-hospital ordering of MOUD therapy medications (listed on Medications tab). | List of medications *ordered* (Date/time) | Visit FIN provided | First order of MOUD med within hospital visit is sufficient |
| Date/time of medication administered | Date/time | Visit FIN provided | First administration of MOUD med within hospital visit is sufficient |
| Insurance type (Primary and Secondary) | Payer name | Visit FIN provided | |
| Primary and secondary diagnoses (problem list for that visit) | ICD codes | Visit FIN provided | Problem list translated to comorbidity score - ICD10 codes vs text? |
| Length of stay | Number | Visit FIN provided | |
| Inpatient admissions in prior 12 months | Number | 12 months prior to visit | |
| 30-day readmissions | Indicator | 30 days since visit FIN provided | |
| Reason for admission | Free response | 30 days since visit FIN provided | |
| Readmission admit date | Date | 30 days since visit FIN provided | |
| Readmission FIN | ID | 30 days since visit FIN provided | |

## APPENDIX B: Medications Prescribed for Treatment Initiation from Electronic Health Record

| **Prescribed MOUD** |
|---|
| Belbuca |
| Bunavail |
| Buprenex |
| buprenorphine |
| Butrans |
| Naltrexone* |
| Probuphine |
| Suboxone |
| Subutex |
| Sublocade |
| Vivitrol* |
| Zubsolv |
| buprenorphine-naloxone (could be written differently depending on the EHR, e.g., buprenorphine/ naloxone; use whatever convention applies for your EHR) |
| Methadone |
| |
| *must be associated w/OUD dx |
