# Supplementary material for: Hospital Addiction Consultation Service and Opioid Use Disorder Treatment: The START Randomized Clinical Trial
Source: JAMA Intern Med. 2025 Apr 7;185(6):624–33. doi: 10.1001/jamainternmed.2024.8586 (PMC11976642; doi:10.1001/jamainternmed.2024.8586)
Supplement: Supplement 2. — Data Sharing Statement [file jamainternmed-e248586-s002.pdf]

Data Sharing Statement

Ober. Hospital Addiction Consultation Service and Opioid Use Disorder Treatment. *JAMA Intern Med.* Published April 07, 2025. doi:10.1001/jamainternmed.2024.8586

Data
Additional Information: ClinicalTrials.gov: NCT05086796.
Data available: Yes
Data types: Deidentified participant data
How to access data: Please send request to pagek@salud.unm.edu
When available: beginning date: 05-31-2025

Supporting Documents
Document types: Statistical/analytic code, Informed consent form
How to access documents: pagek@salud.unm.edu
When available: beginning date: 05-31-2025

Additional Information
Who can access the data: Anyone requesting the data.
Types of analyses: For any purpose.
Mechanisms of data availability: Without investigator support.
